# 3′-*O*-β-Glucosylation of nucleoside analogues using a promiscuous bacterial glycosyltransferase†

**DOI:** 10.1039/d5cb00026b

**Published:** 2025-03-25

**Authors:** Jonathan P. Dolan, Tessa Keenan, Aisling Ní Cheallaigh, Martin A. Fascione, Gavin J. Miller

**Affiliations:** a School of Chemical & Physical Sciences and Centre for Glycoscience, Keele University Keele Staffordshire ST5 5BG UK j.dolan@keele.ac.uk g.j.miller@keele.ac.uk; b Department of Chemistry, University of York York YO10 5DD UK

## Abstract

Nucleoside analogue therapeutics have a proven capability within drug discovery as antiviral and antineoplastic agents. However, their efficacy can be limited by poor cellular uptake, off target toxicity and low bioavailability. Glycosylation of pharmaceutical agents/natural products represents a strategically simple method to modulate pharmacological profiles. Herein, we explore biocatalytic glycosylation of nucleoside analogues. The activity of the nucleoside-specific 3′-*O*-glycosyltransferase AvpGT from *Streptomyces* sp. AVP053U2 is investigated toward a panel of both natural and clinically relevant purine and pyrimidine nucleoside analogues. AvpGT demonstrates broad substrate promiscuity, with glycosylation observed by HILIC-MS for 15 of 21 nucleosides tested. Of these, 12 nucleosides were successfully glycosylated on ≥25 μmol scale in 39–91% isolated yields, including four current therapeutics.

## Abstract 2

Biocatalytic glycosylation of nucleoside analogues using a promiscuous nucleoside-specific 3′-*O*-glycosyltransferase, AvpGT.

## Graphical abstract

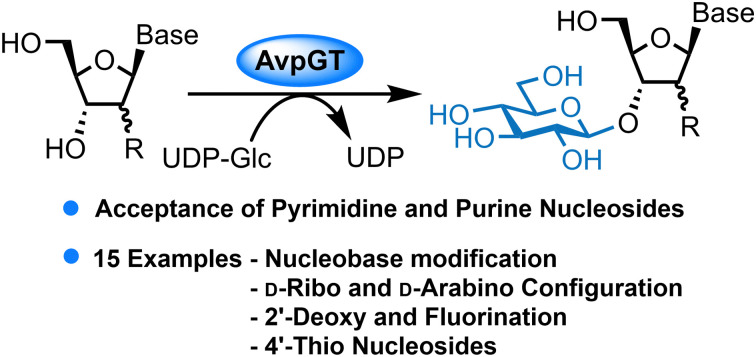

## Introduction

Nucleoside analogues, both synthetic and natural in origin, represent an essential class of small molecule pharmaceutical with broad ranging antiviral and antitumour properties.^1–4^ However, therapeutic intervention using nucleoside analogues can be limited by poor cellular uptake, down regulation of nucleoside transporters, low oral bioavailability, rapid degradation or clearance, development of resistance profiles and limited conversion to the active metabolite.^5,6^ The conjugation of ‘sugars’ to natural products is a rapidly emerging method for tuning pharmacokinetic and pharmacodynamic profiles of a given parent compound. This is also known as glycorandomization, mimicking methods used in nature.^7^ Whilst complex carbohydrates and glycosides fall outside Lipinski's rule of five,^8^ the use of glycosylation in drug discovery is a rapidly emerging area of interest.^9^

The identity of the sugar attached to the aglycone can play a major role in tuning solubility, toxicity, mechanism of action and target recognition.^7,10^ The wide assortment of sugar donors and diversity of glycosylated natural products is exemplified by the more than fifty different sugar nucleotides discovered to date in bacteria, viruses, plants and other organisms.^10,11^ In nature, glycosylation is one of the most common and important methods used to improve solubility and potency of natural products, such that one fifth of bacterial natural products are glycosylated.^12,13^ As such, strategies which mimic this process have proven effective. For example, in preclinical Parkinson′s disease models for the transport of l-DOPA and dopamine, where the resulting prodrugs demonstrated enhanced solubility (more than two orders of magnitude), improved plasma stability, and improved uptake in both human erythrocytes and mouse models.^14,15^ Similar effects have been reported for glycosylated cardiac and flavonoid compounds and their roles as potential anti-cancer agents.^16^ Relatedly, a 3′-*O*-β-glucosyl ribavirin conjugate showed improved antiviral activity in targeting the influenza A RNA-dependent RNA polymerase.^17^ We also recently reported the synthesis of a GLUT1-targeting gemcitabine-glucose prodrug which demonstrated greater efficacy in hormone-resistant PC3 cells over hormone-sensitive LNCaP cells (which have lower levels of GLUT1).^18^

Chemical synthesis approaches to achieve full regio- and stereochemical control for glycorandomisation are non-trivial, exemplified by a synthesis of 3′-*O*-β-glucosyl adenosine requiring eleven steps.^19^ Biocatalysis is a powerful ally to traditional chemical conjugation methods, and its prospect for nucleoside modification is burgeoning.^20^ In nature both 3′-*O*-glycosyl and 5′-*O*-glycosyl nucleoside derivatives are commonly observed. Examples include 5′-*O*-α-glycosyl toyocamycin and tubercidin produced by *Tolypothrix tenuis* and *Plectonema radiosum*, respectively, and 3′-*O*-β-glycosyl-nucleocidin and tubercidin produced by *Streptomyces calvus* and *Streptomyces* sp. AVP053U2, respectively.^19,21,22^ Towards effecting biocatalytic nucleoside glycosylation, we explore herein the substrate profile of the 3′-*O*-β-glycosyltransferase (GT) from *Streptomyces* sp. AVP053U2 (AvpGT) against natural purine and pyrimidine nucleosides alongside a series of clinically relevant nucleoside analogues.

## Results & discussion

Three nucleoside specific GTs have been identified and examined in detail: NucGT from *Streptomyces calvus*,^19,22^ ScaGT from *Streptomyces carminius*,^17^ and AvpGT from *Streptomyces* sp. AVP053U2.^22^ All are members of the GT2 family,^23^ whereby they catalyse glycosyl transfer through an inverting mechanism. Both NucGT and AvpGT share a high degree of amino acid sequence similarity, 66%.^22^ Herein, AvpGT was selected, expressed and purified based on previously published protocols yielding 57 mg_AvpGT_ L_media_^−1^ using standard techniques.^22^ In the host species, AvpGT catalyses the 3′-*O*-glucosylation of tubercidin (7-deazadenosine, 2), a potent antimycobacterial and antineoplastic agent produced by various species of *Streptomyces*.^24^ Previously, work by Pasternak *et al.* showed that AvpGT displayed a promiscuity towards 5′-modification and changes of purine nucleobase (limited to adenosine & guanosine).^22^

### Exploring AvpGT activity towards purine & pyrimidine nucleosides

The specific activity of AvpGT reactions was determined across a range of purine and pyrimidine nucleoside analogues (Table 1). Reaction analysis was performed by monitoring the release of uridine diphosphate from the glucose donor, UDP-glucose, using strong anion exchange HPLC. The formation of glycosylated nucleosides was confirmed by HILIC-MS (see ESI,† Fig. S2–S18).

**Table 1:** Specific activity of AvpGT with purine and pyrimidine nucleosides 1–14. Conventional numbering system for purine and pyrimidine nucleosides is shown for 1 and 11

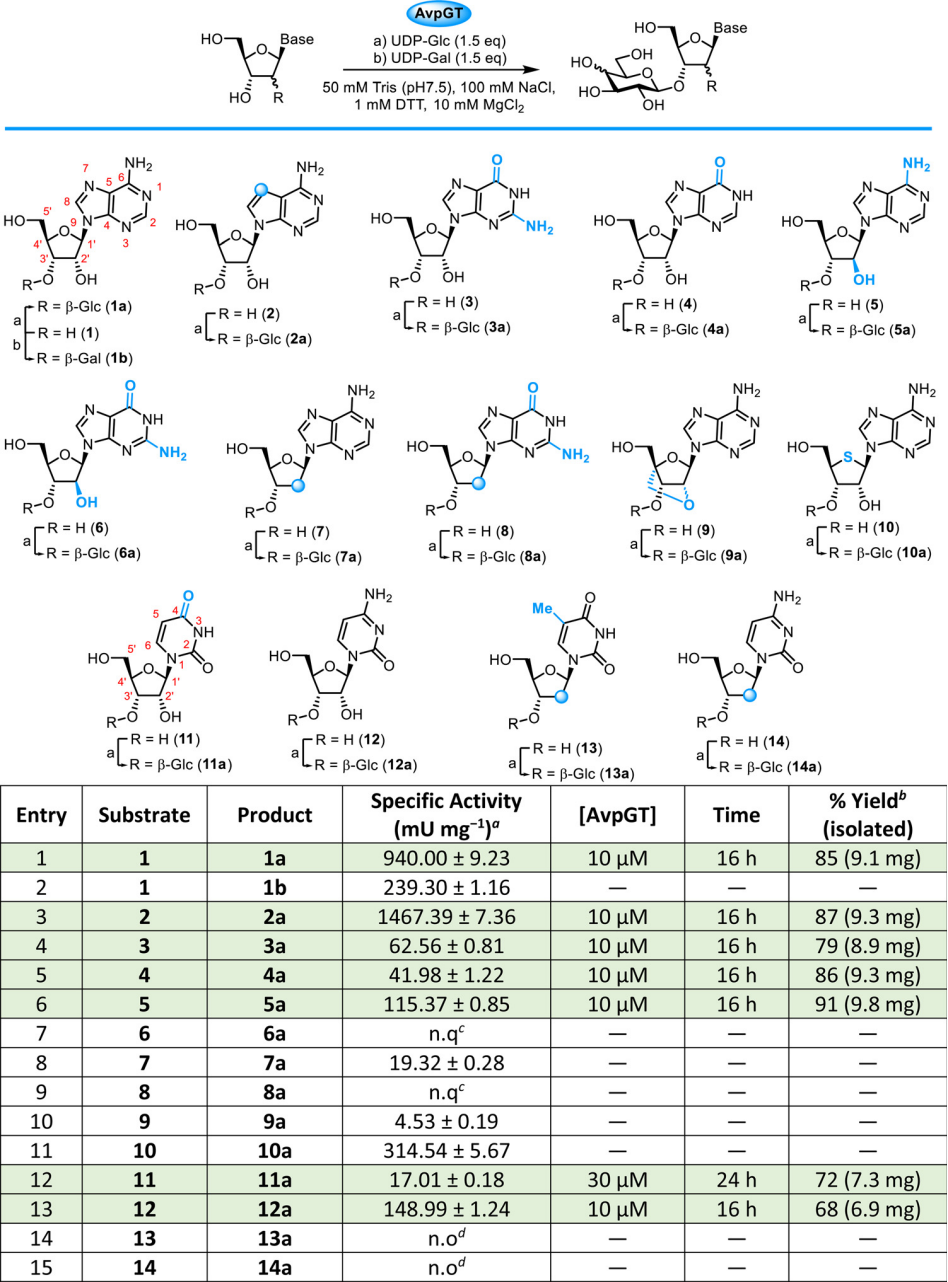

a Assay conditions: substrate (1 mM), UDP-Glc or UDP-Gal (1.5 mM), AvpGT (10 μM), Tris (50 mM, pH 7.5), 100 mM NaCl, 10 mM DTT, 10 mM MgCl_2_, 30 °C, 100 rpm, 1–60 min.

b Isolated yield following purification by semi-prep. HPLC.

c n.q – not quantifiable: UDP release observed by SAX-HPLC (after 60 min), and product formation <5% as observed by HILIC-MS (after 22 h).

d n.o – no product formation observed by HILIC-MS (after 22 h). Blue circles on structures denote structural modification is 2′-deoxy or 7-deaza. Rows highlighted in green were selected for synthesis on a preparative scale using concentration of AvpGT and time indicated followed by HPLC purification.

Based on the kinetic parameters reported previously,^22^ adenosine 1 and tubercidin 2 were superior substrates for AvpGT (Table 1, entries 1 and 3), with activity towards guanosine 3 reduced by ∼15-fold compared to 1 (Table 1, entry 4). Switching the sugar nucleotide donor to UDP-galactose (UDP-Gal) was possible, with ∼3-fold reduction in activity in forming 1b (Table 1, entry 2), compared to the ∼4-fold reduction in activity reported previously.^22^ The enzyme demonstrated no turnover of UDP-*N*-acetyl-glucosamine (UDP-GlcNAc) or UDP-glucuronic acid (UDP-GlcA, see ESI,† Fig. S2–S5). Given the considerable sequence similarity of AvpGT to the recently reported ScaGT (90%),^17^ it is somewhat surprising that AvpGT was incapable of using UDP-GlcA as a donor.

Changing purine 6-position substitution from –NH_2_ in 1 to C=O for inosine 4 showed a reduced activity (Table 1, entry 5), comparable to that observed for 3. Next, we explored altered ribose ring substitution at the 2′-position first investigating the effect of stereochemistry at this position changing from the canonical d-ribo configuration to d-arabino configuration. Using *arabino*adenosine 5 resulted in an ∼8-fold reduction in activity (Table 1, entry 6) compared to adenosine 1 and no quantifiable activity could be detected for *arabino*guanosine 6 (Table 1, entry 7). Furthermore, removal of 2′-OH (2′-deoxy analogues, Table 1) resulted in a ∼50-fold reduction compared to 1 for 2′-deoxyadenosine 7 and no quantifiable activity was evident for guanosine variant 8 (Table 1, entries 8 and 9).

**Fig. 1:**
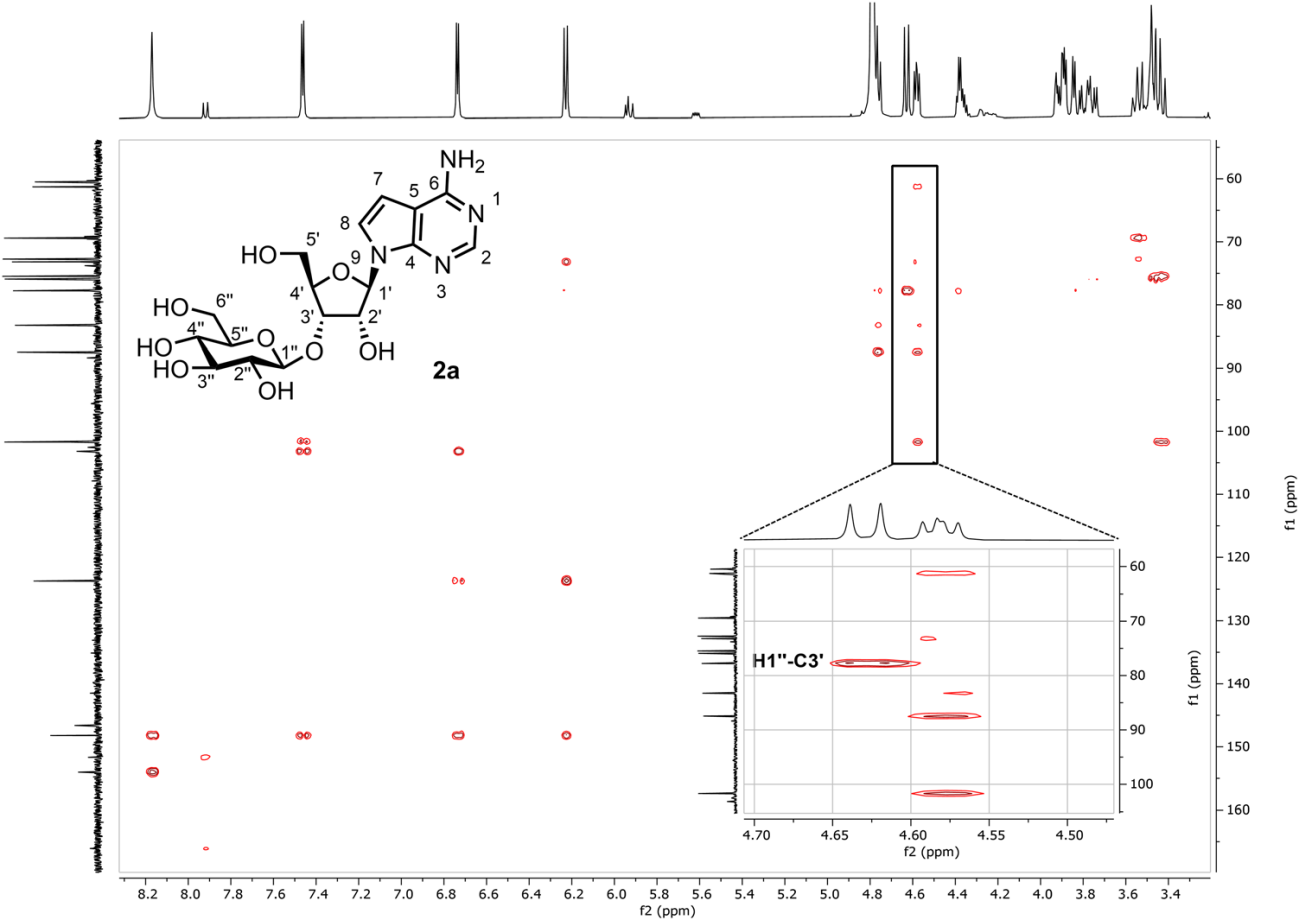
^1^H–^13^C HMBC of 3′-O-β-glucosyl-tubercidin (2a) in D_2_O at 400 MHz showing correlation between the H1′′ on glucose and C3′ on ribose.

Overall, these initial results for purine analogues hint at an importance in maintaining the canonical d-ribo configuration. 2-Position purine base substitution appears unfavourable (substrates 3, 6 and 8), whilst changes at the 6-position are better tolerated. Finally, for purines, we evaluated a locked adenosine analogue 9 (Table 1, entry 10) which showed low activity, alongside 4′-thioadenosine 10,^25^ which was active (Table 1, entry 11), albeit reduced ∼3-fold, compared to 1. In addition, AvpGT demonstrated acceptance of both uridine 11 and cytidine 12 as substrates, with the latter showing ∼9-fold higher activity over uridine (Table 1, entries 12 and 13). This, combined with higher activity observed towards tubercidin 2 and adenosine 1 compared to guanosine 3 and inosine 4, suggests the presence of a hydrogen bond donor within the nucleobase (at C6 in purines and C4 in pyrimidines) may be key for activity. Lastly, and in alignment to results observed for the purine series, 2′-deoxygneation of d-ribose was not tolerated for thymidine 13 or cytidine 14 (Table 1, entries 14 and 15). Substrates 1–5, 11 & 12 were selected for preparative-scale synthesis and purified by semi-preparative RP-HPLC, delivering multi-milligram quantities of glycosylated nucleosides 1a–5a, 11a & 12a in isolated yields of 68–91% (Table 1, green rows). The regioselectivity and stereoselectivity of AvpGT-mediated glycosylation was confirmed as 3′-*O*-β- for each glycoconjugate using a combination of ^1^H–^1^H-decoupled HSQC and ^1^H–^13^C HMBC NMR, illustrated for 2a in Fig. 1.

### AvpGT glycosylates nucleoside analogue therapeutics

We sought next to access 3′-*O*-glycosylated analogues of known nucleoside therapeutics. This included nelarabine 17,^26^ fludarabine 18,^27^ clofarabine 19,^28,29^ and cladribine 20^30^ which act as antimetabolites and are approved treatments for lymphoblastic leukaemia, acute myeloid leukaemia (AML), lymphocytic leukaemia and hairy cell leukaemia. In the case of nelarabine 17, the compound is demethylated to Ara-G 6 by adenosine deaminase (ADA).^26^ Cytarabine (Ara-C) 21, an analogue of cytidine bearing C2′-*arabino* modification, is a frontline treatment for acute leukaemia with high dose Ara-C therapy having the highest antileukemic efficiency of all therapies used to treat AML.^31^ These nucleoside analogue therapeutics were examined using AvpGT and the previously established HILIC-MS and strong anion exchange-HPLC methods (Table 2).

**Table 2:** Specific activity of AvpGT with clinically relevant nucleosides 15–21

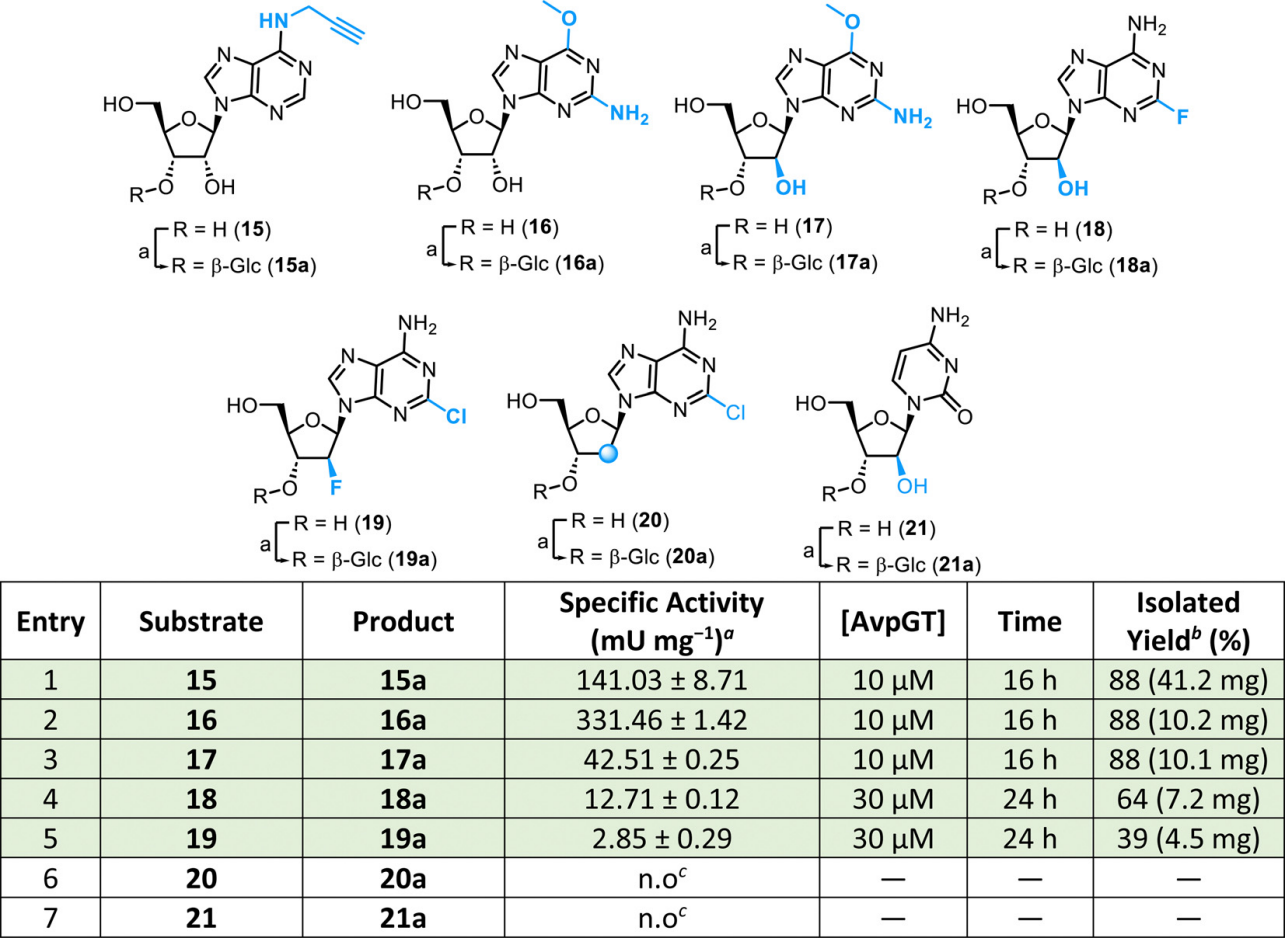

a Assay conditions: substrate (1 mM), UDP-Glc (1.5 mM), AvpGT (10 μM), Tris (50 mM, pH 7.5), 100 mM NaCl, 10 mM DTT, 10 mM MgCl_2_, 30 °C, 100 rpm, 1–60 min.

b Isolated yield following reaction for 16 h and purification by semi-prep HPLC.

c n.o – no product formation observed by HILIC-MS (after 22 h). Rows highlighted in green were selected for synthesis on a preparative scale using concentration of AvpGT and time indicated followed by HPLC purification.

*N*-Propargylation at the 6-position of adenosine 15 was tolerated, but resulted in a moderate loss in activity compared to adenosine 1 (∼7-fold, Table 2, entry 1). However, this result does support a prospect to utilise 15a as a glycosylated nucleoside analogue probe for related nucleic acid biosynthesis, as has been accomplished previously for 15.^32^ C6-*O*-methylation of guanosine 16 interestingly resulted in a ∼5-fold restoration of activity, compared to guanosine 3 (Table 2, entry 2). Acceptance of modifications at this position are unsurprising given the recently reported crystal structure of AvpGT in complex with Ara-A (PDB: 9JN3) showing the presence of a pocket adjacent to the C6-amino group.^17^ Inverting the 2′-position stereochemistry in substrate 16 to give nelarabine 17 showed an ∼8-fold reduction in activity (Table 2, entry 3). Retaining this d-arabino configuration but switching to a C2-halogenated adenosine derivative 18 saw a further ∼4-fold reduction in activity (Table 2, entry 4). A C2 *arabino*fluoro analogue 19, with purine C2 halogenation was the lowest performing analogue tested (Table 2, entry 5). Finally, in this series, cladribine 20 was not active with AvpGT (Table 2, entry 6). Taken together, these purine substrates indicate an exciting activity profile for AvpGT towards glycosylation of nucleoside therapeutics with C2 and C6 nucleobase modifications beyond those observed in canonical systems, alongside accepting a C2′-arabino configuration, but noting that C2′-fluorination or deoxygenation is not well tolerated. Switching to pyrimidines, cytarabine 21 was not active (Table 2, entry 7). With the exception of inactive analogues 20 and 21, *N*^6^-propargyladenosine 15 was scaled to 100 μmol, and all other substrates were scaled to 25 μmol and purified by semi-preparative RP-HPLC providing a small library of glycosylated nucleoside therapeutics (Table 2, green rows).

## Conclusion

In summary, we have established capability for the bacterial glycosyltransferase AvpGT to glycosylate a range of nucleosides, including both natural systems and analogues that are currently used clinically. We exemplify the utility of this enzymatic approach through scalable milligram synthesis of twelve glucosylated conjugates. Deciphering the substrate promiscuity of this enzyme in addition to recently reported capabilities of ScaGT^17^ and NucGT^19^ will open the door to wider exploration of biocatalytic glycoconjugations and scalable nucleoside glycosylation using *in situ* UDP-glucose recycling systems such as sucrose synthase.^33^

## Author contributions

CRediT: Jonathan P. Dolan conceptualization, methodology, investigation, formal analysis, data curation, visualisation, writing – original draft, writing – review & editing; Tessa Keenan methodology, investigation, formal analysis, data curation; Aisling Ní Cheallaigh methodology, resources, supervision; Martin A. Fascione conceptualization, methodology, writing – review & editing, supervision, project administration, funding acquisition; Gavin J. Miller conceptualization, methodology, writing – original draft, writing – review & editing, visualisation, supervision, project administration, funding acquisition.

## Data availability

The data that support the findings of this study have been included as part of the ESI,† and are available from the corresponding author upon reasonable request.

## Conflicts of interest

There are no conflicts to declare.

## Supplementary Material

CB-006-D5CB00026B-s001
