# Supplementary material for: 3′-O-β-Glucosylation of nucleoside analogues using a promiscuous bacterial glycosyltransferase
Source: RSC Chem Biol. 2025 Mar 25;6(6):845–50. doi: 10.1039/d5cb00026b (PMC11959488; doi:10.1039/d5cb00026b)
Supplement: CB-006-D5CB00026B-s001 [file CB-006-D5CB00026B-s001.pdf]

Supplementary Information (SI) for RSC Chemical Biology.
This journal is © The Royal Society of Chemistry 2025

Supporting Information

3'-*O*-β-Glucosylation of nucleoside analogues using a promiscuous bacterial glycosyltransferase

Jonathan P. Dolan,[1*] Tessa Keenan,[2] Aisling Ní Cheallaigh,[1] Martin A. Fascione[2] and Gavin J. Miller[1*]

[1]School of Chemical & Physical Sciences and Centre for Glycoscience, Keele University, Keele, Staffordshire, ST5 5BG, UK.
[2]Department of Chemistry, University of York, York, YO10 5DD, UK.
*Authors for correspondence: j.dolan@keele.ac.uk; g.j.miller@keele.ac.uk

General Experimental 2
- Biochemistry 2
- Chemistry 2

Expression of AvpGT from *Streptomyces* sp. AVP053U2 2
- AvpGT Gene Sequence 3
- AvpGT Expressed Amino Acid Sequence 3
- SDS-PAGE Gel 4

Enzyme Kinetics 4
- Specific Activity Measurements - AvpGT 4

UPLC analysis of AvpGT glycosylation 5

Preparative-scale Biotransformations 19
- General Procedure 19
- Semi-Preparative HPLC Method 20

References 26

NMR Spectra 27

General Experimental

Biochemistry

Sterilisation of media and equipment was carried out in a Prestige Medical bench top autoclave at 121 °C for 15 minutes. Bacterial cultures were incubated in a SciQuip Benchtop INCU-Shake MIDI/MAXI or SciQuip Floor Standing INCU-Shake TL6-5R. LB-agar plates, 96-well assay plates and enzymatic reactions were incubated in a Grant-Bio Orbital Shaker Incubator ES-20, IKA KS4000i Control Incubator or Wolf Laboratories Galaxy R CO_2 Incubator. Centrifugation was performed using either a Sorvall RC5Cplus, SciQuip Sigma 2-16P, Eppendorf 5810 or Beckman Coulter™ Microfuge20. SDS-PAGE was carried out using Bio-Rad mini protean 3 apparatus using Bio-Rad mini-PROTEAN TGX precast gels. Proteins were concentrated either using 10k or 30k MWCO Amicon® Ultra-15 centrifugal filter device. Analytical grade reagents were used as supplied by commercial suppliers. Bacterial plasmid DNA extraction & purification performed using GeneJET plasmid miniprep kit (ThermoFisher) following the manufactures recommended protocol.

Chemistry

All reagents and solvents which were available commercially were purchased from Biosynth, Acros, Alfa Aesar, Fisher Scientific, Sigma Aldrich or TCI. All reactions in non-aqueous solvents were conducted with oven-dried glassware with a magnetic stirring device under an inert atmosphere of nitrogen passed through a drying column using a vacuum manifold. Solvents were purified by passing through activated alumina columns and transferred under nitrogen unless otherwise stated. Reactions were followed by thin layer chromatography (TLC) using Merck silica gel 60 F_{254} analytical plates (aluminium support) and were developed using short wave UV radiation (245 nm), 10% H_2SO_4 in methanol/Δ or *p*-anisaldehyde/Δ. Purification *via* flash column chromatography was conducted manually using Sigma Aldrich silica gel 60 (0.040-0.063 mm) under a positive pressure of compressed air. Analytical HPLC was performed using Agilent 1220 Infinity LC. Preparative purification by HPLC was conducted on Agilent 1260 Infinity II.

^{1}H NMR spectra were recorded at 400 MHz, ^{13}C NMR spectra at 100 MHz and ^{19}F NMR spectra at 376 MHz respectively using Bruker Magnet system 400'54 Ascend (400 MHz). ^{1}H NMR resonances were assigned with the aid of gDQCOSY. ^{13}C NMR resonances were assigned with the aid of gHSQCAD. Coupling constants are reported in Hertz. Chemical shifts (δ, in ppm) are standardised against the deuterated solvent peak. NMR data were analyzed using Mestrenova. ^{1}H NMR splitting patterns were assigned as follows: br.s (broad singlet), s (singlet), d (doublet), dd (doublet of doublets), ddd (doublet of doublet of doublets), app. t (apparent triplet), t (triplet), quartet (q) or m (multiplet and/or multiple resonances). HRMS were obtained on Agilent LC-QToF (UPLC 2390 Infinity II & QToF 6530B) employing electrospray (ES+) ionization.

Expression of AvpGT from *Streptomyces* sp. AVP053U2

The pET28a-*avpGT* plasmid encoding AvpGT with an N-terminal His_6 tag was kindly received as a gift from the group of David L. Zechel (Queen's University, Ontario, Canada). The transformant was grown according to the literature.[1]

Briefly, the plasmid was transformed into BL21(DE3) star chemically competent cells (ThermoFisher), according to the manufacturer's protocol, and cultivated on LB-Agar (+Kanamycin (50 µg/mL)) at 37 °C. A single colony was used to inoculate 5 mL of 2YT medium containing 30 µg/mL Kanamycin and incubated overnight at 37 °C and 200 rpm. The overnight culture was used to inoculate 2 × 1 L of LB medium containing 30 µg/mL and grown at 37 °C with shaking at 200 rpm in baffled flasks until an OD_{600} of ~0.8 was reached. The cultures were incubated on ice and the incubator temperature reduced to 15 °C. After 30 minutes on ice, the cultures were induced with 1 mM IPTG and returned to the cooled incubator. Induction was allowed to continue overnight (21 hrs) at 15 °C, shaking at 200 rpm. The bacterial cell pellet was harvested by centrifugation (10,000 *x g*, 15 mins, 4 °C). The wet cell pellet was resuspended in Lysis buffer (50 mM Tris (pH 7.8), 250 mM NaCl, 0.3 mM TCEP and $5\%_{v/v}$ glycerol to $20\%_{w/v}$ then supplemented with DNaseA (10 µg/mL, Roche) and 10 mM imidazole. The bacterial suspension was lysed by sonication (On: 2 seconds; Off: 3 seconds; 5 minutes; max temp: 15 °C; 40% amplitude) on ice. The insoluble cell debris was cleared *via* centrifugation (30,000 *x g*, 45 mins, 4 °C), then filtered through a 0.45 µm filter and loaded onto a 5 mL pre-equilibrated Cytiva Ni-NTA column. The protein was eluted following a stepwise gradient elution in which 5 CV of lysis buffer containing 50, 100, 150, 200, 300, 500 mM imidazole were flowed through the column and collected as a single fraction. These fractions were combined, concentrated and exchanged into Lysis buffer using a 10 MWCO Amicon centrifugal filter. Aliquots were flash-frozen in liquid N_2 and stored at –80 °C.

AvpGT Gene Sequence

ATGGGCAGCAGCCATCATCATCATCATCACAGCAGCGGCCTGGTGCCGCGCGGCAGCCATGTGCTGGTCAGTGTCATCAT
CCCCACCTACAACCGTCCGGAACGCCTGGCCGTCGCGCTGCAGAGCGTGCAGACCCTGGACTTCGACAGTGAGCAGCTCG
AGGTCATCGTGGTCAACGACCACGGCACCCCGGTCGATGACGTGGTCGAGGCTGCCGGCCGGTCCCTGAACGTACGCCT
CATCGACCAGCCGTCCCAGAGCGGGCCGTCGGGGGCGCGCAACGCCGGGCTGGAGGTGGCCCGCGGAGAGTACGTCGC
GTTCCTCGACGACGACGACGTCTTCAGCCCACAGCACCTGAGCGGCACCCTTCCCCTGCTGAAGGGCGGGGCCGACTTCG
TCTACGTCAACATCAACATCGCGCGGACGCGGGTCACCGGTACGACGATCGCCGACGCCGAGGTCCTCGTTCGGCTGGA
GTTCCCCTACGACCGCGGGCTGCTGGACGTCACCAACCACTTCGCCCCGTCGGCCGTGGTGTGCCGTTCCCCCGGTCCGC
CGGGGCGTTCTTCGACACGGCGCTGGGCGTCGAGGAGGACTGGGACTTCTTCCTGAGGCTGGCACACGGACACAAGTAC
CGGGTGGTGCACCAGCCGGAGGTCGCCATCGCCCTGCACCGCATCCCCGGCGTCGAGAGCCTGACGACACCGACCTCCG
ACGACATCGCGGCCCTGAAGGTCTACGAGGACAACTGGCACCTGATCTGCGAGCGCTGGCCGGCCGCGACCGAGCGCGC
CGAGCAGGTGCGCCGGTTCATGCCGGTGATGTACCAGATGGCGTACGCCAGCTTCGAAGCGGGCGTTCCGCTGGACCAC
CACTACTACGAGCGCACGCTCCAGGTCCTCTACCGCGCGCTCGGCGACCCGCAGCCCTCCCCCGCGCAGGTCGAGGACGA
GCTGCGGGCGGCTCTGGAGGGCCGCTGA

AvpGT Expressed Amino Acid Sequence

MGSSHHHHHHSSGLVPRGSHVLVSVIIPTYNRPERLAVALQSVQTLDFDSEQLEVIVVNDHGTPVDDVVEAAGRSLNVRLIDQ
PSQSGPSGARNAGLEVARGEYVAFLDDDDVFSPQHLSGTLPLLKGGADFVYVNINIARTRVTGTTIADAEVLVRLEFPYDRGLLD
VTNHFAPSAVVCRSPRSAGAFFDTALGVEEDWDFFLRLAHGHKYRVVHQPEVAIALHRIPGVESLTTPTSDDIAALKVYEDNW
HLICERWPAATERAEQVRRFMPVMYQMAYASFEAGVPLDHHYYERTLQVLYRALGDPQPSPAQVEDELRAALEGR*

SDS-PAGE Gel

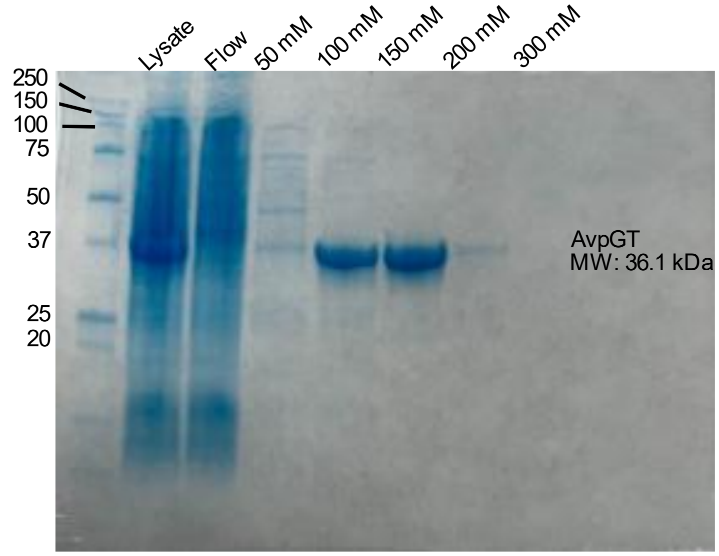

Figure S1: *Purification of AvpGT by Ni-NTA affinity chromatography. Elution's performed following a stepwise imidazole gradient using 5 CV for each elution. The concentration of imidazole used for elution is shown above each lane.*

Enzyme Kinetics

Specific Activity Measurements - AvpGT

All specific activity measurements were performed using analytical SAX-HPLC monitoring the consumption of UDP-Glc and production of UDP at a single substrate concentration. Reactions were prepared using 1 mM nucleoside substrate, 1.5 mM UDP-Glc, and 10 µM AvpGT (361 µg mL^{-1}), 50 mM Tris (pH 7.5), 100 mM NaCl, 10 mM $MgCl_2$ and 1 mM DTT at 30 °C. Each reaction was repeated in triplicate at a final volume of 50 µL. After 1, 5 or 15 minutes, 50 µL of ethanol was added to each reaction (1:1 ratio of ethanol to reaction volume) and incubation completed at −20 °C for 4 hours before clarification by centrifugation (17,800 *x g*, 10 mins). Samples were analyzed by analytical SAX-HPLC using an Agilent PL-SAX (8µm 150 × 4.6mm) (PL1551-3802) at a flow rate of 0.5 mL/min. Eluents: A) 0.01M NaCl, B) 1M NaCl; Method: hold at 0%B for 3 mins, gradient to 25%B over 10 mins, gradient to 100%B over 0.5 min, hold at 100%B for 3 mins, gradient to 0%B over 0.5 min, hold at 0%B for 5 mins; Oven Temperature: 32 °C; Injection volume: 10 µL; UV detection: 260 nm.

Specific activity was calculated using the following equation:

$$Specific\ Activity = \frac{\left(\frac{([\mathrm{UDP}] \times V)}{T}\right)}{\mathrm{AvpGT\ (mg)}} \times 10^3$$

Where [UDP] is calculated from a standard curve based on the area under the peak for UDP, V is the volume of the reaction in µL, T is the time of reaction completion in minutes. Values were multiplied by 10^3 to give specific activity in mU mg^{-1}.

UPLC analysis of AvpGT glycosylation

Method

AvpGT reactions were performed in reaction buffer [50 mM Tris (pH7.5), 100 mM NaCl, 10 mM $MgCl_2$, 1 mM DTT] plus 1 mM nucleoside acceptor substrate, 1.5 mM UDP-sugar donor (1.5 equivalents), and 10 µM AvpGT on a 100 µL scale. Reactions were incubated at 30 °C for 21 hrs. The reactions were prepared for HILIC-MS analysis by first precipitating the AvpGT with the addition of ethanol (1:1 ratio of ethanol to reaction volume) and incubation at −20 °C for 4 hours before clarification by centrifugation (17,800 x g, 10 mins) followed by lyophilisation and storage at −20 °C. Prior to analysis samples were dissolved in 3:2 HPLC water/acetonitrile (50 µL).

Reaction products were identified using: Method A: Accucore HILIC HPLC column (2.6 µm particle size, 50 × 2.1 mm) using a mixture of Milli-Q water + 0.1% formic acid (A) and acetonitrile + 0.1% formic acid (B) according to the following scheme: hold at 95%B for 1 min; Linear gradient to 5%B over 9.5 mins; hold at 5%B for 0.5 mins; linear gradient to 95%B over 1 min; hold at 95%B for 3 mins. The flow rate was 0.3 mL/min and the absorbance of the eluent was monitored at 260 nm. Method B: Avantor ACE HILIC-N HPLC column (3 µm particle size, 250 × 3.0 mm) using a mixture of Milli-Q water + 0.1% formic acid (A) and acetonitrile + 0.1% formic acid (B) according to the following scheme: hold at 95%B for 3 min; Linear gradient to 5%B over 10 mins; hold at 5%B for 3 mins; linear gradient to 95%B over 1 min; hold at 95%B for 5 mins. The flow rate was 1.0 mL/min and the absorbance of the eluent was monitored at 260 nm.

For both methods sample concentration was maintained at 1 mM and injection volume was 5 µL. Reactions are compared to nucleoside standards which are run using the same HILIC-MS conditions. Reaction quantified based on area under the peak; where >95% defined as quantitative.

Adenosine (1) + UDP-Glucose

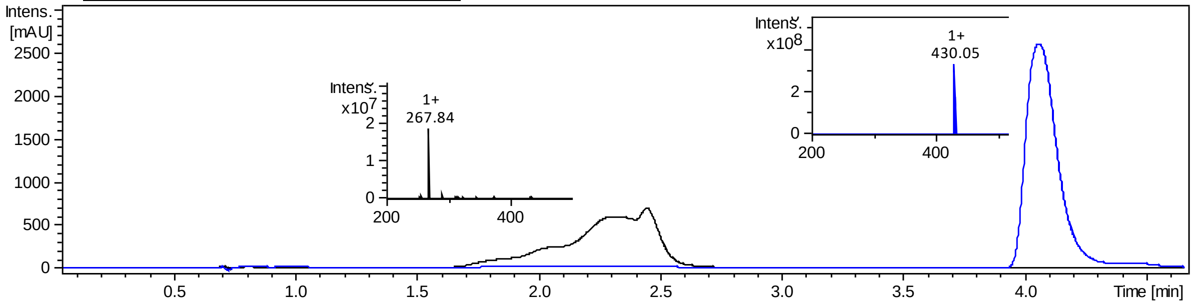

Figure S2: *UPLC-HILIC-MS (method A) analysis of an overnight AvpGT reaction with adenosine (blue) & adenosine standard (black). Inset mass spectra for indicated peaks; Theoretical [M+H]$^+$ for adenosine m/z = 268.10, 3'-O-β-glucosyl-adenosine m/z = 430.16.*

Quantitative product formation (product signals & mass spectrum (blue) for signal between 3.9-4.3 mins) following 22hr incubation with AvpGT.

Adenosine (1) + UDP-Galactose

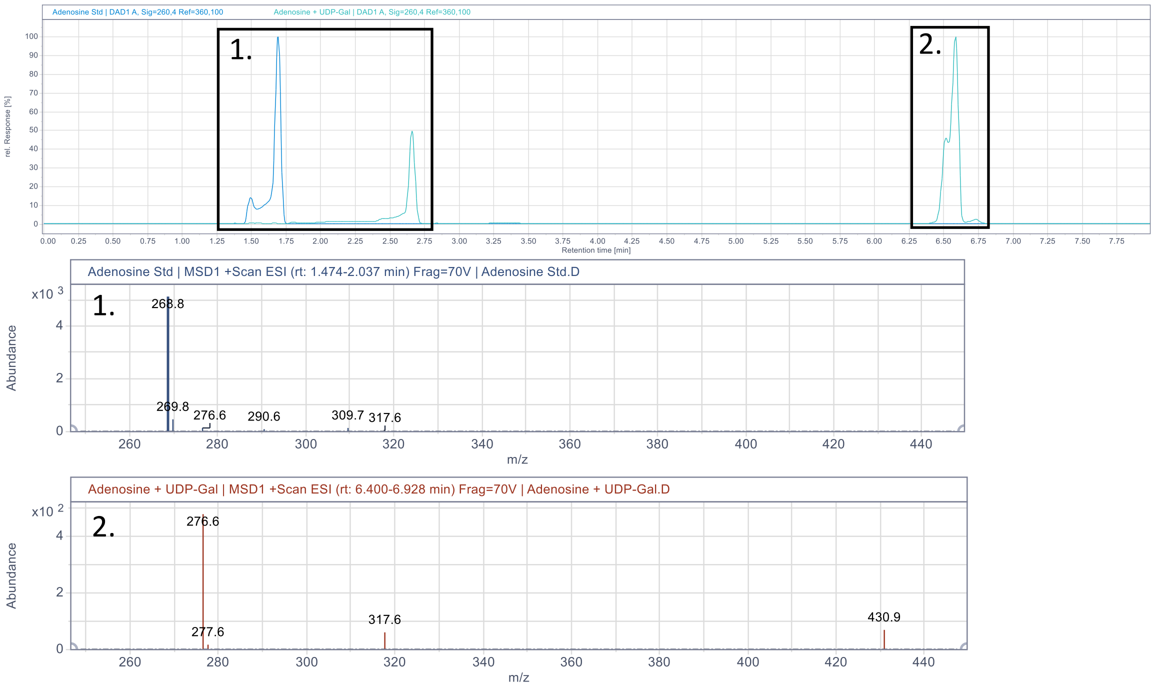

Figure S3: *UPLC-HILIC-MS (method B) analysis of an overnight AvpGT reaction with adenosine (teal) & adenosine standard (blue). Underneath: mass spectra for indicated peaks; Theoretical* $[M+H]^+$ *for adenosine m/z = 268.10, 3'-O-β-galactosyl-adenosine m/z = 430.16.*

70% product formation (product signals & mass spectrum (teal, peak 2) for signal between 6.4-6.9 mins) following 22hr incubation with AvpGT.

Adenosine (1) + UDP-N-Acetyl-glucosamine

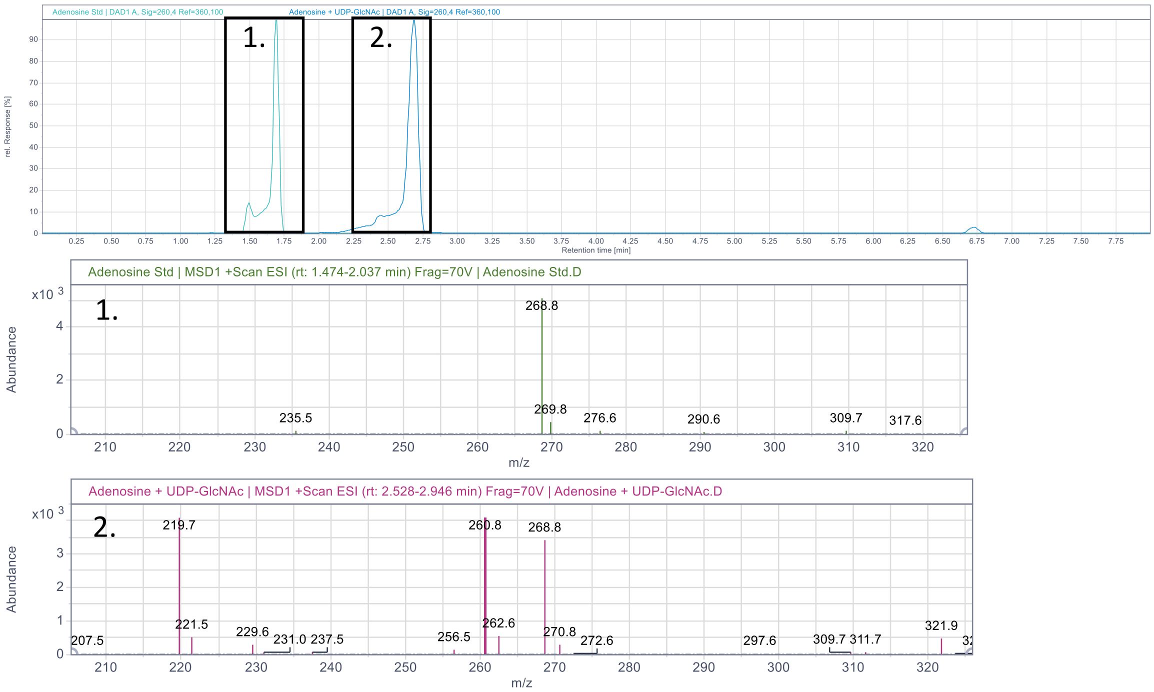

Figure S4: *UPLC-HILIC-MS (method B) analysis of an overnight AvpGT reaction with adenosine (teal) & adenosine standard (blue). Underneath: mass spectra for indicated peaks; Theoretical $[M+H]^+$ for adenosine m/z = 268.10, 3'-O-β-GlcNAc-adenosine m/z = 444.16.*

No product formation observed. Mass spectrometry deconvolution of peaks 2 shows no signals which correspond with that of the expected m/z for the N-acetyl-glucosamine glycosylated adenosine following 22hr incubation with AvpGT. Spectrum of peak 2 only shows adenosine (268.8).

Adenosine (1) + UDP-glucuronic acid

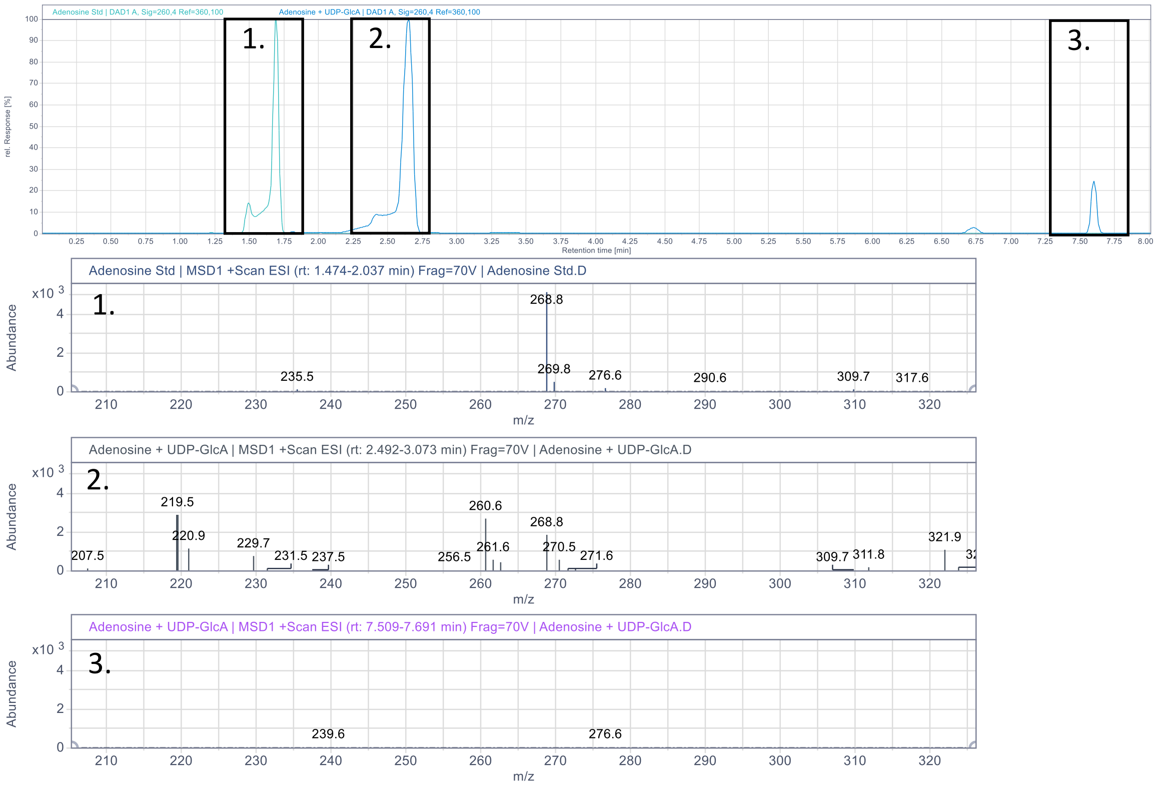

Figure S5: *UPLC-HILIC-MS (method B) analysis of an overnight AvpGT reaction with adenosine (blue) & adenosine standard (teal). Underneath: mass spectra for indicated peaks; Theoretical $[M+H]^+$ for adenosine m/z = 268.10, 3'-O-β-glucuronic-adenosine m/z = 444.16.*

No product formation observed. Mass spectrometry deconvolution of peaks 2 & 3 show no signals which correspond with that of the expected m/z for the glucuronic acid glycosylated adenosine following 22hr incubation with AvpGT. Spectrum of peak 2 only shows adenosine (268.8). Spectrum of peak 3 shows no masses larger than 276.6 Da and no peaks which correspond to expected fragments.

Tubercidin (7-deazaadenosine) (2)

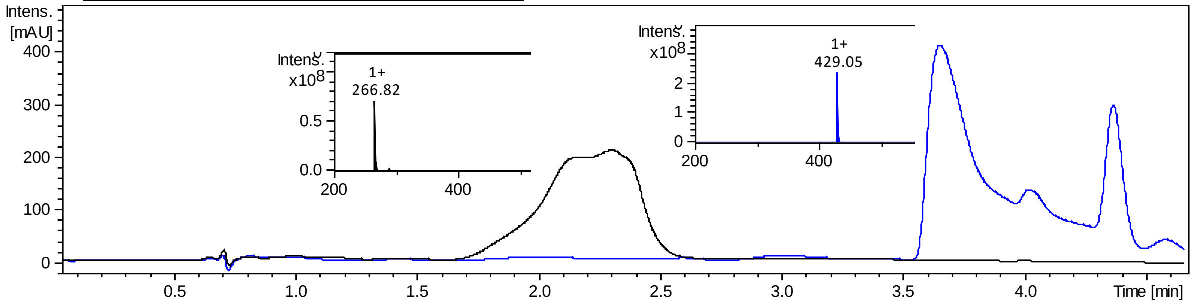

Figure S6: *UPLC-HILIC-MS (method A) analysis of an overnight AvpGT reaction with Tubercidin (blue) & Tubercidin standard (black). Inset mass spectra for indicated peaks; Theoretical $[M+H]^+$ for Tubercidin m/z = 267.11, 3'-O-β-glucosyl-tubercidin m/z = 429.16.*

Quantitative product formation (product signals & mass spectrum (blue) for signal between 3.55-4.65 mins) following 22hr incubation with AvpGT.

Guanosine (3)

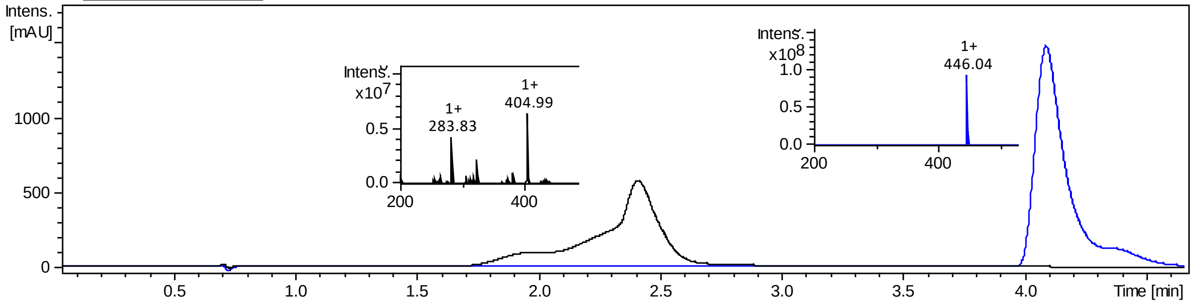

Figure S7: *UPLC-HILIC-MS (method A) analysis of an overnight AvpGT reaction with guanosine (blue) & guanosine standard (black). Inset mass spectra for indicated peaks; Theoretical $[M+H]^+$ for guanosine m/z = 284.10, 3'-O-β-glucosyl-guanosine m/z = 446.15.*

Quantitative product formation (product signals & mass spectrum (blue) for signal between 3.95-4.65 mins) following 22hr incubation with AvpGT.

Inosine (4)

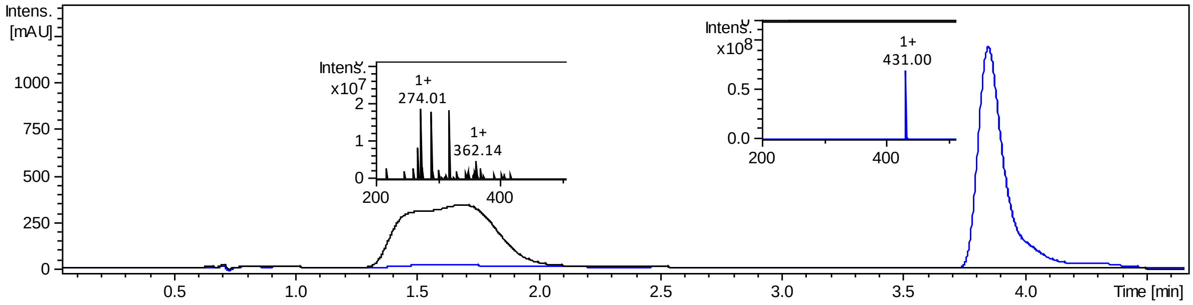

Figure S8: *UPLC-HILIC-MS (method A) analysis of an overnight AvpGT reaction with Inosine (blue) & Inosine standard (black). Inset mass spectra for indicated peaks; Theoretical $[M+H]^+$ for Inosine m/z = 269.09, 3'-O-β-glucosyl-Inosine m/z = 431.14.*

Quantitative product formation (product signals & mass spectrum (blue) for signal between 3.75-4.20 mins) following 22hr incubation with AvpGT.

9-(3'-O-Glucosyl-β-D-arabinofuranosyl)adenine (Ara-A, Vidarabine) (5)

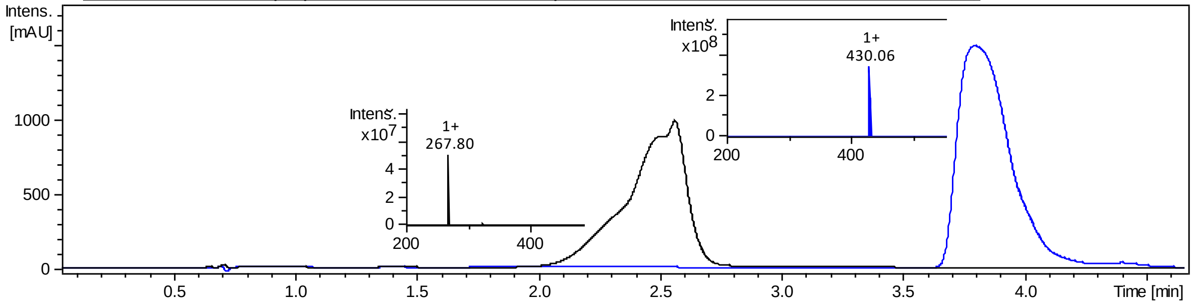

Figure S9: *UPLC-HILIC-MS (method A) analysis of an overnight AvpGT reaction with Ara-A (blue) & Ara-A standard (black). Inset mass spectra for indicated peaks; Theoretical $[M+H]^+$ for Ara-A m/z = 268.10, 3'-O-β-glucosyl-Ara-A m/z = 430.16.*

Quantitative product formation (product signals & mass spectrum (blue) for signal between 3.65-4.25 mins) following 22hr incubation with AvpGT.

9-(3'-O-Glucosyl-β-D-arabinofuranosyl)guanine (Ara-G) (6)

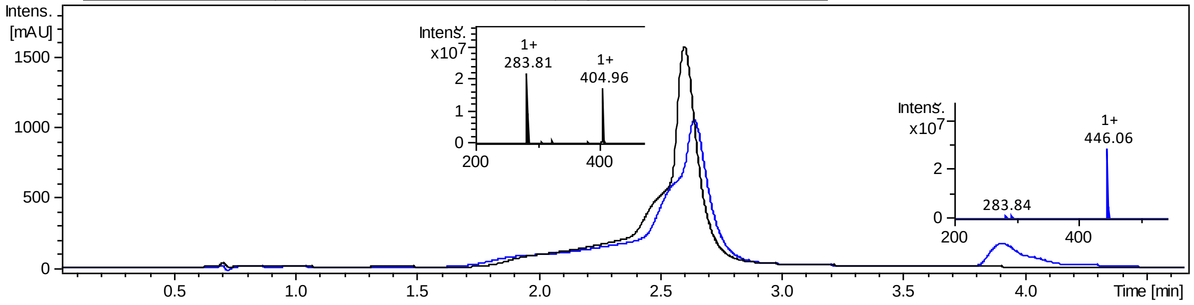

Figure S10: *UPLC-HILIC-MS (method A) analysis of an overnight AvpGT reaction with Ara-G (blue) & Ara-G standard (black). Inset mass spectra for indicated peaks; Theoretical $[M+H]^+$ for Ara-G m/z = 284.10, 3'-O-β-glucosyl-Ara-G m/z = 446.15.*

Minimal product formation (product signals & mass spectrum (blue) for signal between 3.90-4.30 mins) following 22hr incubation with AvpGT.

2'-Deoxyadenosine (7)

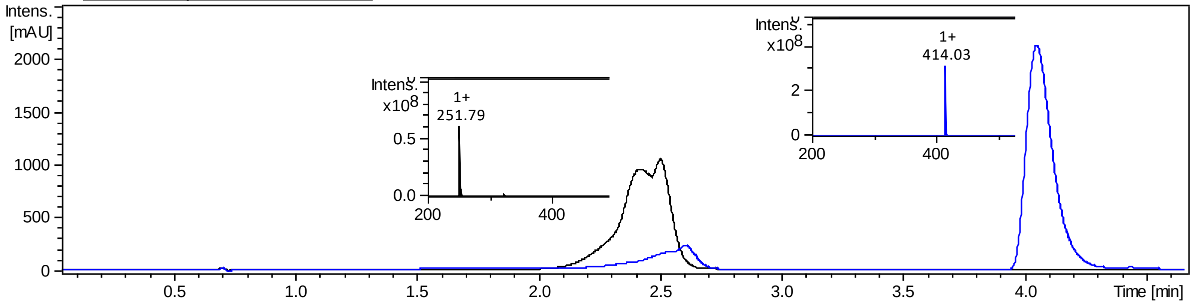

Figure S11: *UPLC-HILIC-MS (method A) analysis of an overnight AvpGT reaction with 2'-deoxy-adenosine (blue) & 2'-deoxy-adenosine standard (black). Inset mass spectra for indicated peaks; Theoretical $[M+H]^+$ for 2'-deoxy-adenosine m/z = 252.11, 3'-O-β-glucosyl-2'-deoxy-adenosine m/z = 414.16.*

92% conversion to product (product signals & mass spectrum (blue) for signal between 3.95-4.35 mins) following 22hr incubation with AvpGT.

2'-Deoxyguanosine (8)

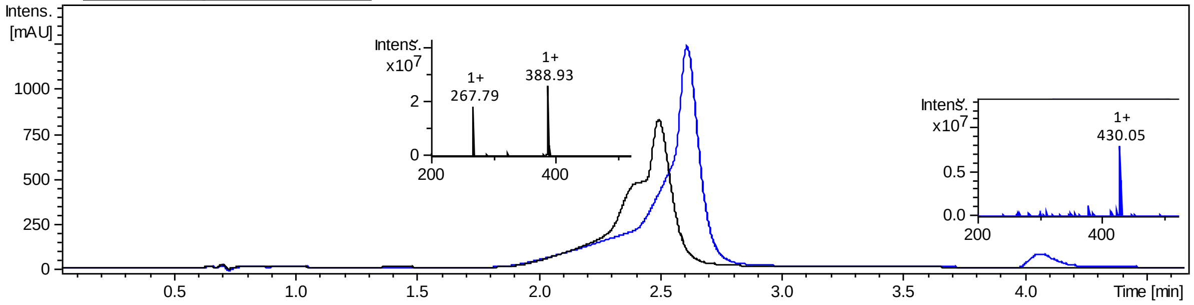

Figure S12: *UPLC-HILIC-MS (method A) analysis of an overnight AvpGT reaction with 2'-deoxy-guanosine (blue) & 2'-deoxy-guanosine standard (black). Inset mass spectra for indicated peaks; Theoretical $[M+H]^+$ for 2'-deoxy-guanosine m/z = 268.10, 3'-O-β-glucosyl-2'-deoxy-guanosine m/z = 430.16.*

Minimal product formation (product signals & mass spectrum (blue) for signal between 3.95-4.25 mins) following 22hr incubation with AvpGT.

LNA-Adenosine (9)

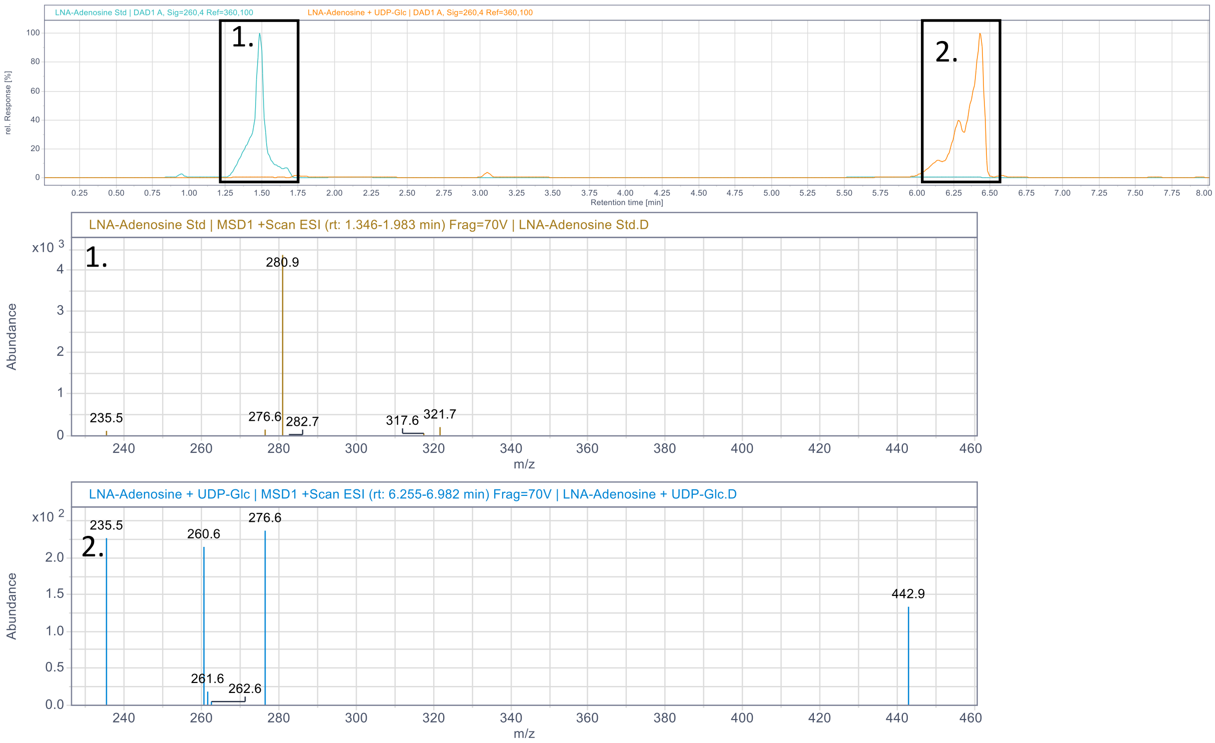

Figure S13: *UPLC-HILIC-MS (method B) analysis of an overnight AvpGT reaction with LNA-adenosine (orange) & LNA-adenosine standard (teal). Underneath: mass spectra for indicated peaks; Theoretical [M+H]$^+$ for LNA-adenosine m/z = 279.1, 3'-O-β-glucosyl-LNA-adenosine m/z = 442.16.*

Quantitative product formation (product signals & mass spectrum (orange Peak 2) for signal between 6.26-6.98 mins) following 22hr incubation with AvpGT.

4’-Thioadenosine (10)

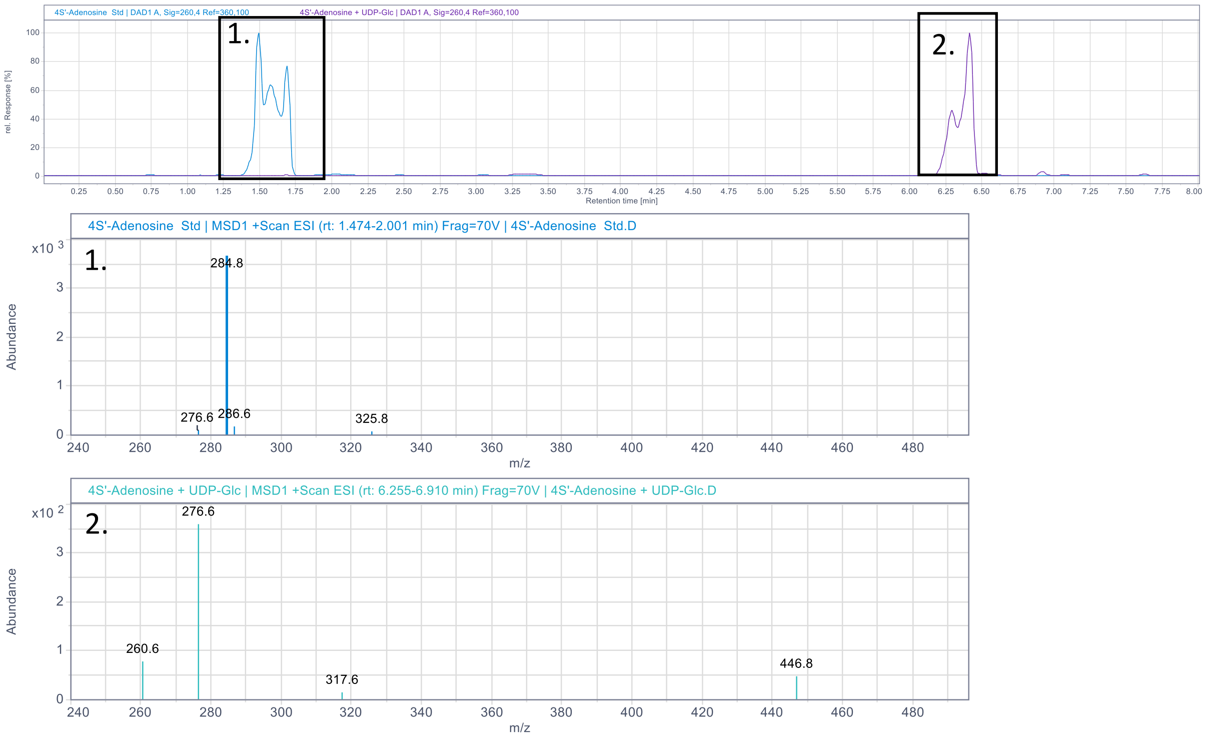

Figure S14: *UPLC-HILIC-MS (method B) analysis of an overnight AvpGT reaction with 4’-thio-adenosine (purple) & 4’-thio-adenosine standard (blue). Underneath: mass spectra for indicated peaks; Theoretical [M+H]$^+$ for 4’-thio-adenosine m/z = 284.1, 3’-O-β-glucosyl-4’-thio-adenosine m/z = 446.2.*

Quantitative product formation (product signals & mass spectrum (purple trace, Peak 2) for signal between 6.26-6.91 mins) following 22hr incubation with AvpGT.

Uridine (11)

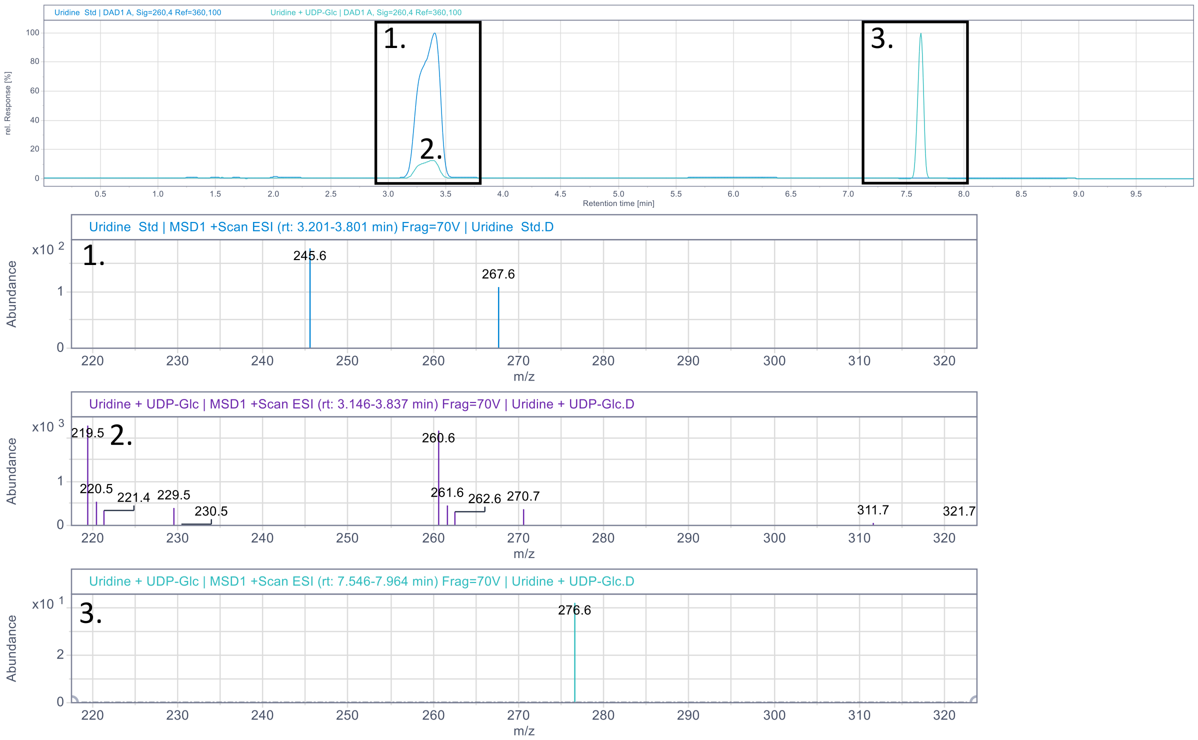

Figure S15: *UPLC-HILIC-MS (method B) analysis of an overnight AvpGT reaction with uridine (teal) & uridine standard (blue). Underneath: mass spectra for indicated peaks; Theoretical $[M+H]^+$ for uridine m/z = 245.1, 3'-O-β-glucosyl-uridine m/z = 407.2.*

Result unclear & further investigated by scale up & SAX-HPLC to detect UDP formation. Reaction appears to result in quantitative product formation (product signals (purple trace, Peak 3) for signal between 7.55-7.96 mins) following 22hr incubation with AvpGT. Mass spectrum of peak 2 shows no remaining uridine & spectrum of peak 3 results in low intensities. Compound hypothesised to poorly ionise.

Cytidine (12)

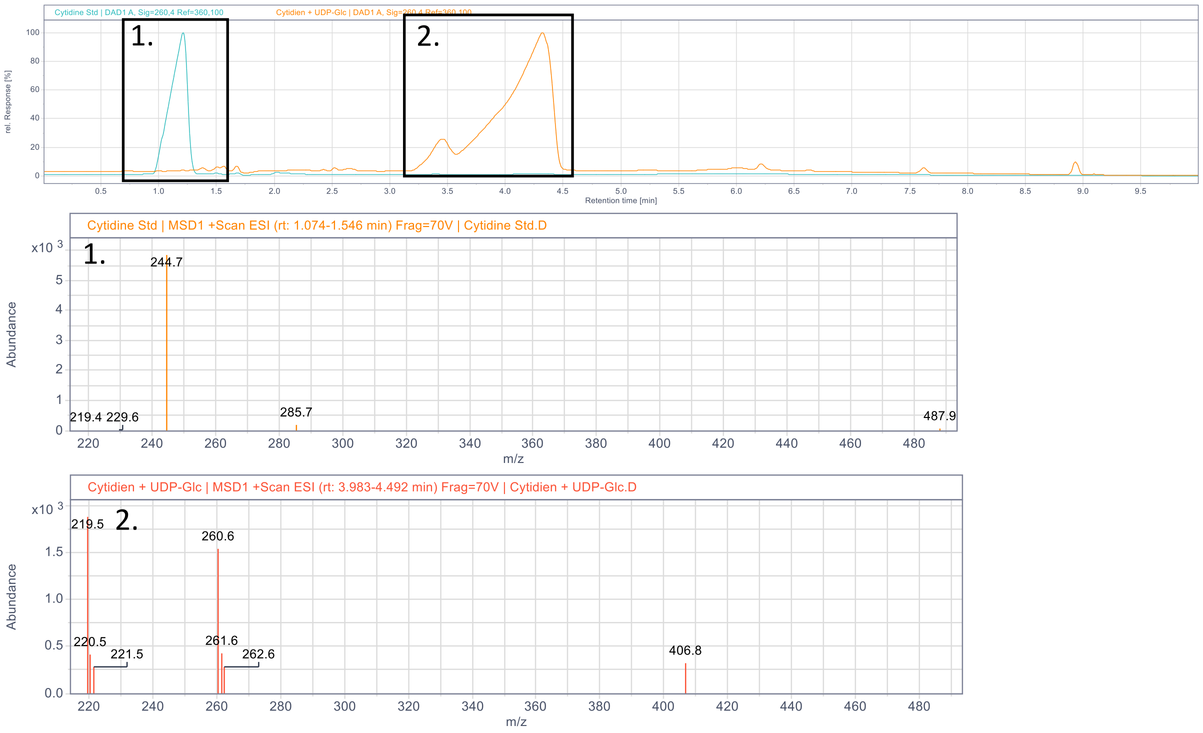

Figure S16: *UPLC-HILIC-MS (method B) analysis of an overnight AvpGT reaction with cytidine (orange) & cytidine standard (teal). Underneath: mass spectra for indicated peaks; Theoretical $[M+H]^+$ for cytidine m/z = 244.1, 3'-O-β-glucosyl-cytidine m/z = 406.3.*

Quantitative product formation (product signals & mass spectrum (orange trace, Peak 2) for signal between 3.98-4.49 mins) following 22hr incubation with AvpGT.

Thymidine (13)

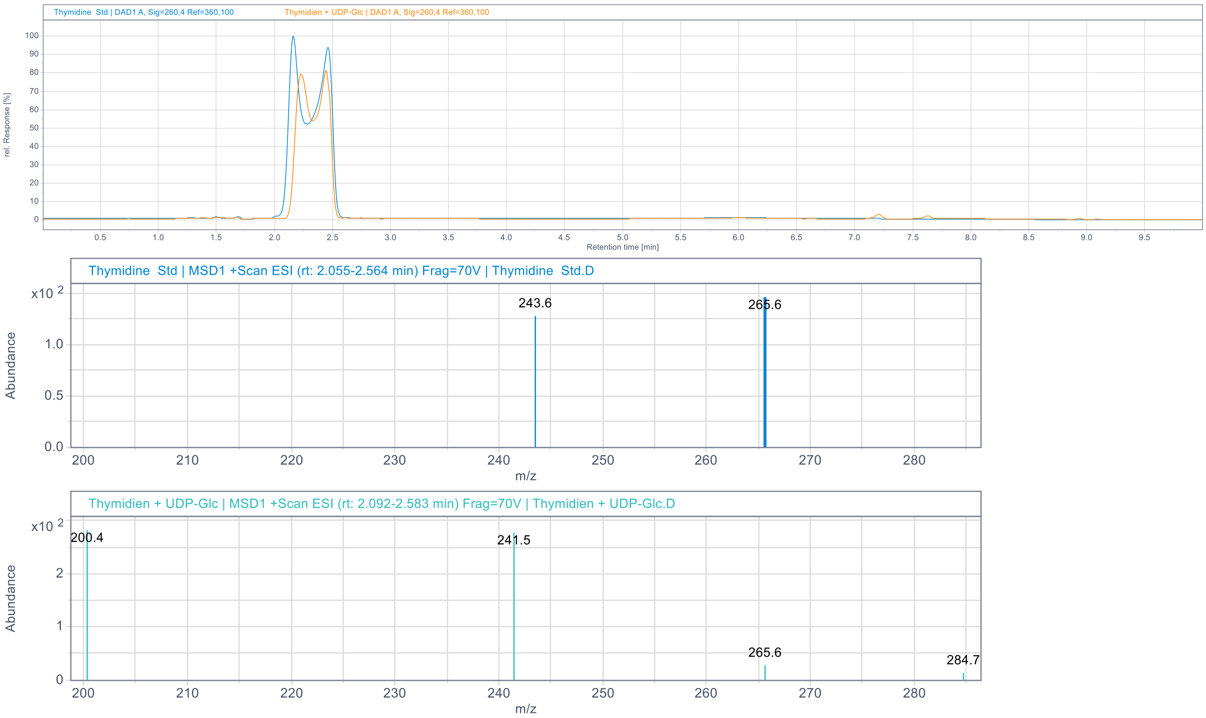

Figure S17: *UPLC-HILIC-MS (method B) analysis of an overnight AvpGT reaction with thymidine (orange) & thymidine standard (blue). Underneath: mass spectra for indicated peaks; Theoretical $[M+H]^+$ for thymidine m/z = 243.1, 3'-O-β-glucosyl-thymidine m/z = 405.3.*

No product formation observed. Mass spectrometry deconvolution of peaks show no signals which correspond with that of the expected m/z for the glycosylated thymidine & no peak shift is observed following 22hr incubation with AvpGT.

2-Deoxycytidine (14)

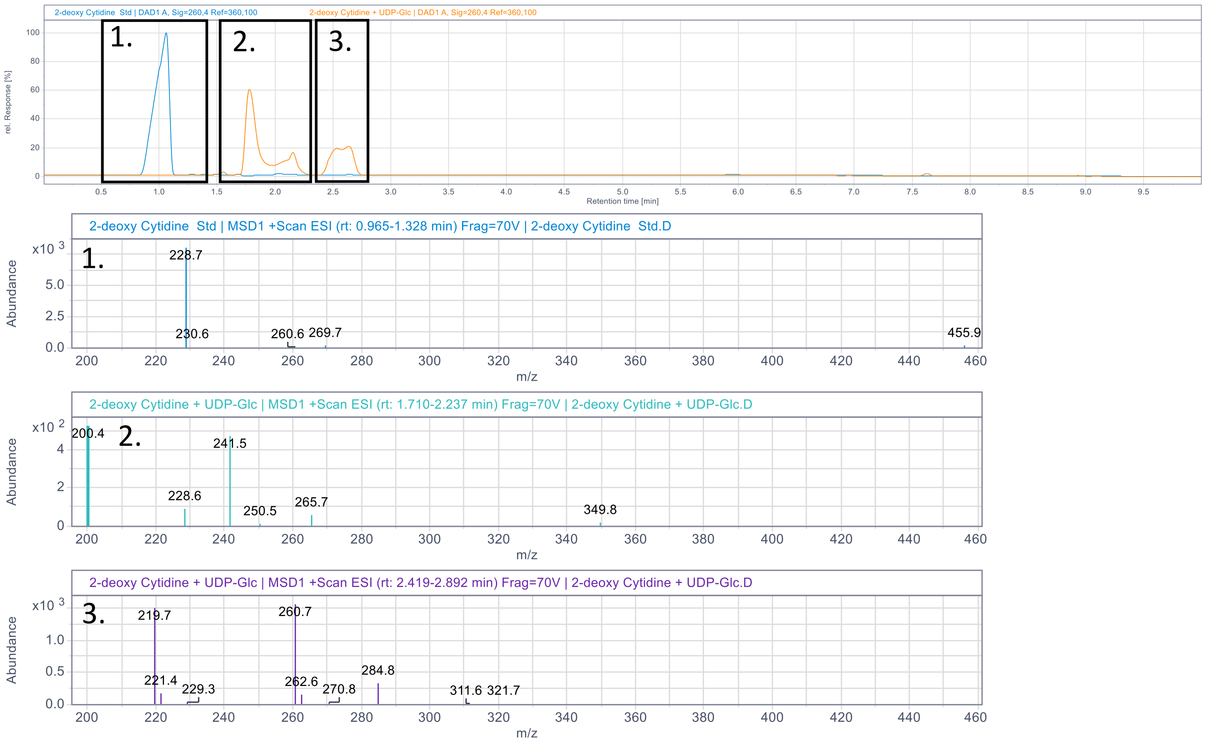

Figure S18: *UPLC-HILIC-MS (method B) analysis of an overnight AvpGT reaction with 2-deoxy-cytidine (orange) & 2-deoxy-cytidine standard (blue). Underneath: mass spectra for indicated peaks; Theoretical $[M+H]^+$ for 2-deoxy-cytidine m/z = 228.1, 3'-O-β-glucosyl-2-deoxy-cytidine m/z = 390.3.*

No product formation observed. Mass spectrometry deconvolution of peaks 2 & 3 show no signals which correspond with that of the expected m/z for the glycosylated 2-deoxy-cytidine following 22hr incubation with AvpGT. Spectrum of peak 2 only shows 2-deoxy-cytidine (228.6). Spectrum of peak 3 shows no masses larger than 321.7 Da and no peaks which correspond to expected fragments.

*N*⁶-Propargyladenosine (15)

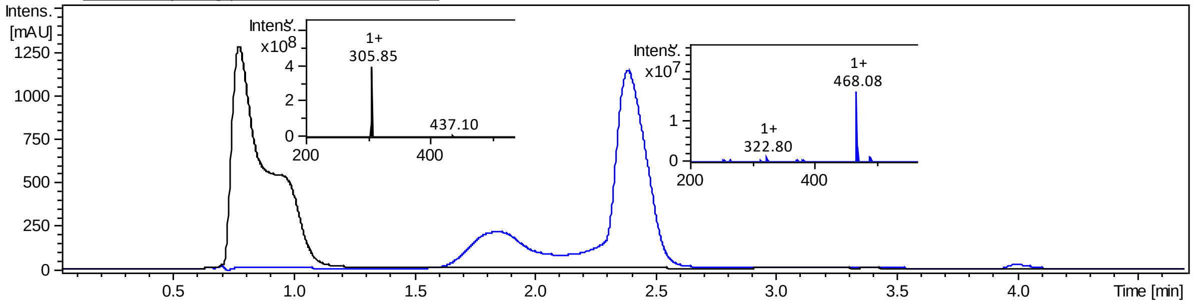

Figure S19: *UPLC-HILIC-MS (method A) analysis of an overnight AvpGT reaction with N^6-propargyl-adenosine (blue) & N^6-propargyl-adenosine standard (black). Inset mass spectra for indicated peaks; Theoretical $[M+H]^+$ for N^6-propargyl-adenosine m/z = 306.12, 3'-O-β-glucosyl-N^6-propargyl-adenosine m/z = 468.17.*

Quantitative conversion to product (product signals & mass spectrum (blue) for signal between 1.65-2.65 mins) following 22hr incubation with AvpGT.

6-*O*-Methylguanosine (16)

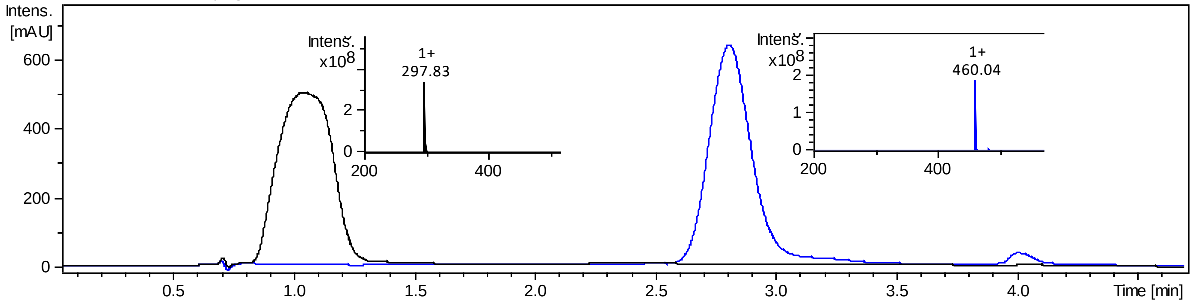

Figure S20: *UPLC-HILIC-MS (method A) analysis of an overnight AvpGT reaction with 6-O-methyl-guanosine (blue) & 6-O-methyl-guanosine standard (black). Inset mass spectra for indicated peaks; Theoretical [M+H]$^+$ for 6-O-methyl-guanosine m/z = 298.11, 3'-O-β-glucosyl-6-O-methyl-guanosine m/z = 460.17.*

Quantitative conversion to product (product signals & mass spectrum (blue) for signal between 2.60-3.40 mins) following 22hr incubation with AvpGT.

Nelarabine (17)

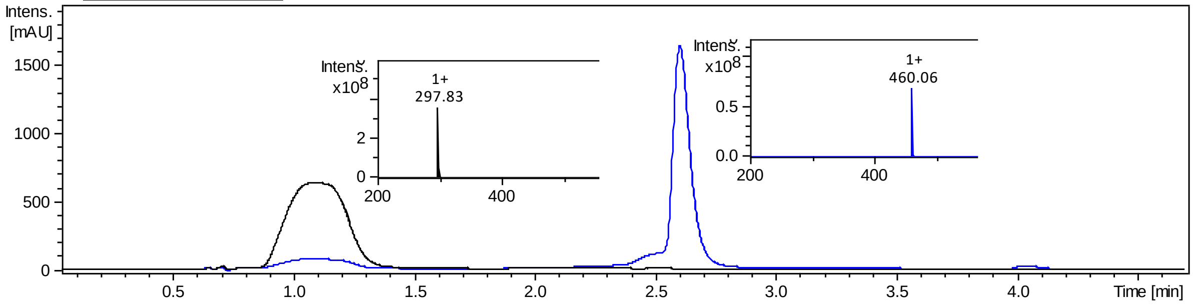

Figure S21: *UPLC-HILIC-MS (method A) analysis of an overnight AvpGT reaction with Nelarabine (blue) & Nelarabine standard (black). Inset mass spectra for indicated peaks; Theoretical [M+H]$^+$ for Nelarabine m/z = 298.11, 3'-O-β-glucosyl-nelarabine m/z = 460.17.*

91% conversion to product (product signals & mass spectrum (blue) for signal between 2.35-2.85 mins) following 22hr incubation with AvpGT.

Fludarabine (18)

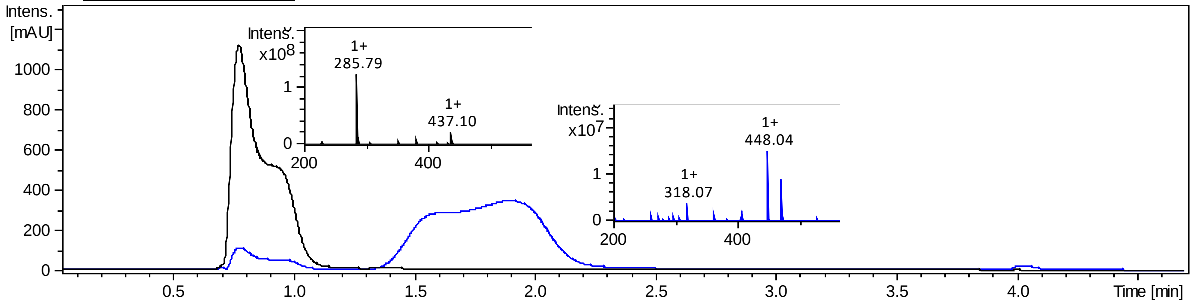

Figure S22: *UPLC-HILIC-MS (method A) analysis of an overnight AvpGT reaction with Fludarabine (blue) & Fludarabine standard (black). Inset mass spectra for indicated peaks; Theoretical [M+H]$^+$ for Fludarabine m/z = 286.09, 3'-O-β-glucosyl-fludarabine m/z = 448.15.*

96% conversion to product (product signals & mass spectrum (blue) for signal between 1.35-2.30 mins) following 22hr incubation with AvpGT.

Clofarabine (19)

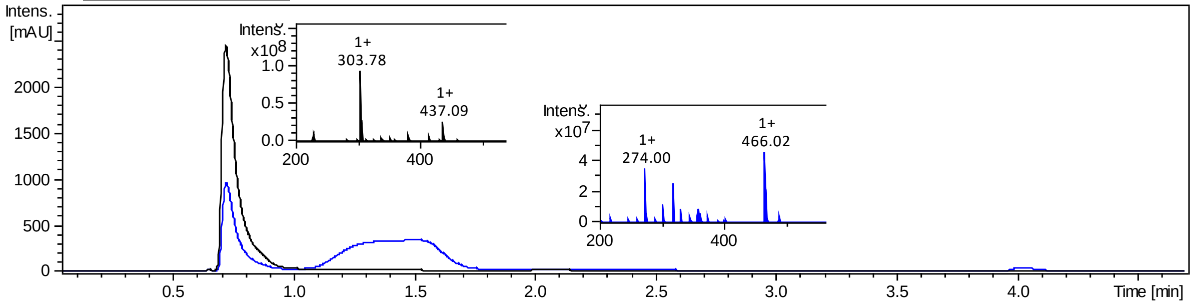

Figure S23: *UPLC-HILIC-MS (method A) analysis of an overnight AvpGT reaction with Clofarabine (blue) & Clofarabine standard (black). Inset mass spectra for indicated peaks; Theoretical $[M+H]^+$ for Clofarabine m/z = 304.06, 3'-O-β-glucosyl-clofarabine m/z = 466.11.*

50% conversion to product (product signals & mass spectrum (blue) for signal between 1.05-1.75 mins) following 22hr incubation with AvpGT.

Cladribine (20)

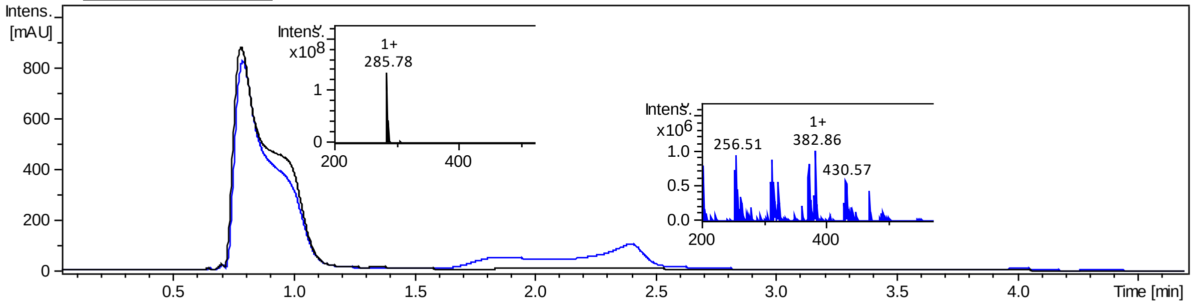

Figure S24: *UPLC-HILIC-MS (method A) analysis of an overnight AvpGT reaction with Cladribine (blue) & Cladribine standard (black). Inset mass spectra for indicated peaks; Theoretical $[M+H]^+$ for Cladribine m/z = 286.07, 3'-O-β-glucosyl-cladribine m/z = 448.12.*

No product formation observed. Mass spectrometry deconvolution of peaks at 1.7-2.5 mins show no signals which correspond with that of the expected m/z for the glycosylated cladribine following 22hr incubation with AvpGT.

Cytarabine (21)

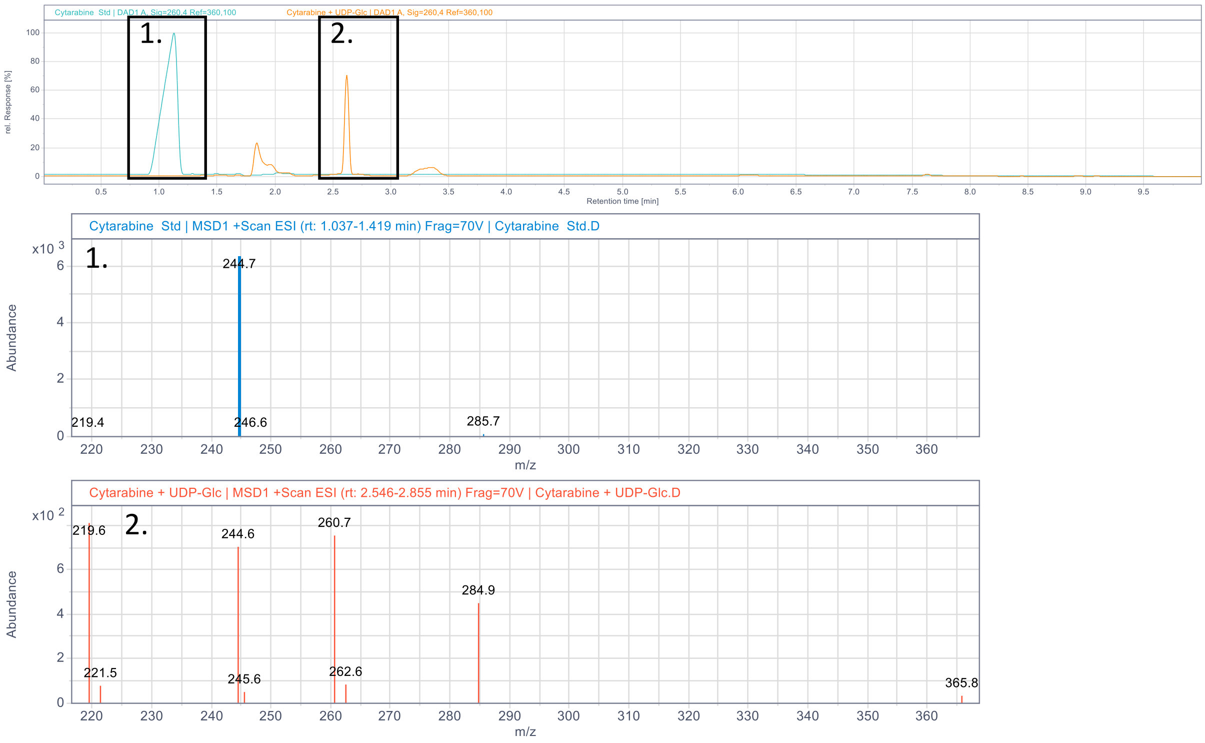

Figure S25: *UPLC-HILIC-MS (method B) analysis of an overnight AvpGT reaction with cytarabine (orange) & cytarabine standard (teal). Underneath: mass spectra for indicated peaks; Theoretical $[M+H]^+$ for cytarabine m/z = 244.1, 3'-O-β-glucosyl-cytidine m/z = 406.3.*

No product formation observed. Mass spectrometry deconvolution of peaks 2 show no signals which correspond with that of the expected m/z for the glycosylated cytarabine following 22hr incubation with AvpGT. Spectrum of peak 2 only shows cytarabine (268.8) and no peaks which correspond to expected fragments of glycosylated nucleoside.

Preparative-scale Biotransformations

General Procedure

Method A: 10 µM AvpGT

A 15 mL centrifuge tube was charged with the nucleoside substrate (25 µmol, 10 mM, 1.0 eq) and UDP-Glucose ($2Na^+$) (22.9 mg, 37.5 µmol, 15 mM, 1.5 eq) then suspended in 2.15 mL of d.H_2O. The reaction was made up to volume first with 250 µL of a 10× stock of the reaction buffer (Final concs.: 50 mM Tris (pH7.5), 100 mM NaCl, 1 mM DTT), $MgCl_2$ (25 µL, stock conc. 1 M, final conc. 10 mM) and finally AvpGT (71.4 µL, stock conc. 350 µM, final conc. 10 µM, 0.1 mol%). The reactions were incubated at 30 °C for 16 hrs with vigorous shaking. The reactions were monitored by TLC (3:2 IPA/35% aq. NH_4OH). Once the reaction had reached completion, ice cold ethanol (2.5 mL) was added, and the reaction placed in a –20 °C freezer for 4 hours to precipitate the enzyme. The supernatants were concentrated first *in vacuo*, then lyophilised before being purified by semi-preparative HPLC.

Method B: 30 µM AvpGT

Reaction performed as per method A with the following amendments:

1. Reactants suspended in 2.04 mL of d.H_2O.
2. AvpGT concentration increased (214.3 µL, stock conc. 350 µM, final conc. 30 µM, 0.3 mol%).

Semi-Preparative HPLC Method

Samples were purified on Polaris 5 C18-A column (5 µm particle size, 250 × 10mm) using a mixture of Milli-Q water + 0.1% formic acid (A) and methanol (B) according to the following scheme: hold at 4%B for 10 mins; Linear gradient from 4%B to 60%B over 10 mins; Linear gradient from 60%B to 96%B over 1 min; Hold at 96%B for 5 mins; Linear gradient from 96%B to 4%B over 1 min; Hold at 4%B for 5 mins. The flow rate was 5 mL/min and the absorbance of the eluent was monitored at 260 nm. Sample concentration 10 mg/mL, Injection volume 200 µL.

3′-*O*-β-Glucosyladenosine (1a)

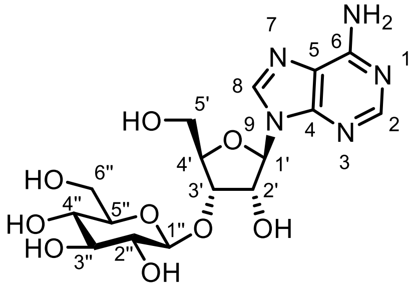

Chemical Formula: $C_{16}H_{23}N_5O_9$
Exact Mass: 429.1496
Molecular Weight: 429.3860

Method A; Yield: 9.1 mg, 85%; **R_f** 0.57 (3:2 IPA/35% aq. NH_4OH); **^{1}H NMR** (400 MHz; D_2O) δ 8.30 (1 H, s, **H_8**), 8.18 (1 H, s, **H_2**), 6.09 (1 H, d, *J* = 6.0 Hz, **$H_{1'}$**), 4.88 (1 H, t_{app}, *J* = 5.9 Hz, **$H_{2'}$**), 4.63 (1 H, d, *J* = 7.3 Hz, **$H_{1''}$**), 4.62 (1 H, d, *J* = 9.1 Hz, **$H_{3'}$**), 4.44 (1 H, q, *J* = 6.9, 3.3 Hz, **$H_{4'}$**), 3.95 (1 H, dd, *J* = 12.7, 2.7 Hz, **$H_{5a'}$**), 3.90 (1 H, dd, *J* = 12.3, 1.9 Hz, **$H_{6a''}$**), 3.84 (1 H, dd, *J* = 12.7, 3.7 Hz, **$H_{5b'}$**), 3.75 (1 H, dd, *J* = 12.3, 5.4 Hz, **$H_{6b''}$**), 3.57–3.40 (4 H, m, **$H_{2''}$**, **$H_{3''}$**, **$H_{4''}$**, **$H_{5''}$**); **^{13}C NMR** (100 MHz; D_2O) δ 155.4 (**C_6**), 152.3 (**C_2**), 148.4 (**C_4**), 140.5 (**C_8**), 119.0 (**C_5**), 101.8 (**$C_{1''}$**), 88.4 (**$C_{1'}$**), 83.9 (**$C_{4'}$**), 77.8 (**$C_{3'}$**), 75.9 (**$C_{4''}$**), 75.5 (**$C_{3''}$**), 73.2 (**$C_{2'}$**), 72.7 (**$C_{2''}$**), 69.4 (**$C_{5''}$**), 61.3 (**$C_{5'}$**), 60.5 (**$C_{6''}$**); **HRMS** [ES+] found $[M+H]^+$ 430.1560, $C_{16}H_{23}N_5O_9$+H requires 430.1569, error −2.09 ppm.

3′-*O*-β-Glucosyl-7-deazaadenosine (3′-O-β-glucosyltubercidin) (2a)

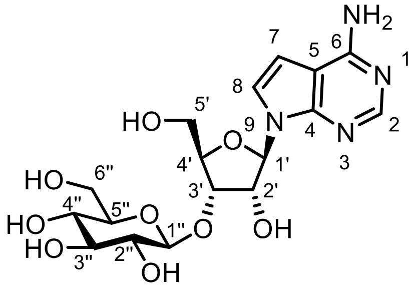

Chemical Formula: $C_{17}H_{24}N_4O_9$
Exact Mass: 428.1543
Molecular Weight: 428.3980

Method A; Yield: 9.3 mg, 87%; **R_f** 0.59 (3:2 IPA/35% aq. NH_4OH); **^{1}H NMR** (400 MHz; D_2O) δ 8.17 (1 H, s, **H_2**), 7.47 (1 H, d, *J* = 3.9 Hz, **H_8**), 6.74 (1 H, d, *J* = 3.8 Hz, **H_7**), 6.23 (1 H, d, *J* = 6.2 Hz, **$H_{1'}$**), 4.77 (1 H, t, *J* = 5.9 Hz, **$H_{2'}$**), 4.63 (1 H, d, *J* = 7.8 Hz, **$H_{1''}$**), 4.58 (1 H, dd, *J* = 5.4, 3.6 Hz, **$H_{3'}$**), 4.38 (1 H, q, *J* = 3.7 Hz, **$H_{4'}$**), 3.92 (1 H, dd, *J* = 12.3, 1.9 Hz, **$H_{6a''}$**), 3.90 (1 H, dd, *J* = 12.6, 3.4 Hz, **$H_{5a'}$**), 3.83 (1 H, dd, *J* = 12.6, 4.1 Hz, **$H_{5b'}$**), 3.76 (1 H, dd, *J* = 12.3, 5.1 Hz, **$H_{6b''}$**), 3.57–3.40 (4 H, m, **$H_{2''}$**, **$H_{3''}$**, **$H_{4''}$**, **$H_{5''}$**); **^{13}C NMR** (100 MHz; D_2O) δ 154.0 (**C_6**), 148.2 (**C_4**), 146.6 (**C_2**), 123.7 (**C_8**), 103.2 (**C_5**), 101.7 (**$C_{1''}$**), 101.6 (**C_7**), 81.7 (**$C_{1'}$**), 83.2 (**$C_{4'}$**), 77.8 (**$C_{3'}$**), 75.9 (**$C_{5''}$**), 75.5 (**$C_{2'}$**), 72.7 (**$C_{2''}$**), 69.4 (**$C_{4''}$**), 61.3 (**$C_{5'}$**), 60.5 (**$C_{6''}$**); **HRMS** [ES+] found $[M+H]^+$ 429.1605, $C_{17}H_{25}N_4O_9$ requires 429.1616, error −2.56 ppm.

3′-*O*-β-Glucosylguanosine (3a)

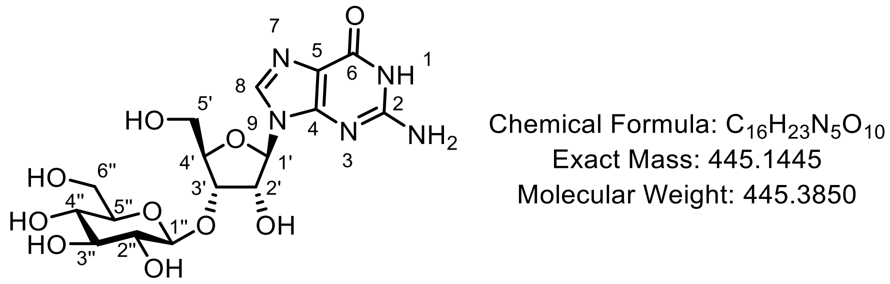

Method A; Yield: 8.9 mg, 79%; **R_f** 0.35 (3:2 IPA/35% aq. NH_4OH); **^{1}H NMR** (400 MHz; D_2O) δ 7.99 (1 H, s, **H_8**), 5.95 (1 H, d, *J* = 5.7 Hz, **$H_{1'}$**), 4.83 (1 H, t$_{app}$, *J* = 5.4 Hz, **$H_{2'}$**), 4.62 (1 H, d, *J* = 8.3 Hz, **$H_{1''}$**), 4.59 (1 H, d, *J* = 5.1 Hz, **$H_{3'}$**), 4.38 (1 H, q, *J* = 7.6, 4.0 Hz, **$H_{4'}$**), 3.95–3.88 (2 H, m, **$H_{5a'}$**, **$H_{6a''}$**), 3.83 (1 H, dd, *J* = 12.7, 4.0 Hz, **$H_{5b'}$**), 3.75 (1 H, dd, *J* = 12.3, 5.1 Hz, **$H_{6b''}$**), 3.57–3.39 (4H, m, **$H_{2''}$**, **$H_{3''}$**, **$H_{4''}$**, **$H_{5''}$**); **^{13}C NMR** (100 MHz; D_2O) δ 158.9 (**C_6**), 153.8 (**C_2**), 151.3 (**C_4**), 137.6 (**C_8**), 116.6 (**C_5**), 101.8 ($C_{1''}$), 87.9 (**$C_{1'}$**), 83.5 (**$C_{4'}$**), 77.7 (**$C_{3'}$**), 75.9 (**$C_{4''}$**), 75.5 (**$C_{3''}$**), 73.0 (**$C_{2'}$**), 72.7 (**$C_{2''}$**), 69.4 (**$C_{5''}$**), 61.1 (**$C_{5'}$**), 60.5 (**$C_{6''}$**); **HRMS** [ES+] found $[M+H]^+$ 446.1512, $C_{16}H_{24}N_5O_{10}$ requires 446.1518, error −1.34 ppm.

3′-*O*-β-Glucosylinosine (4a)

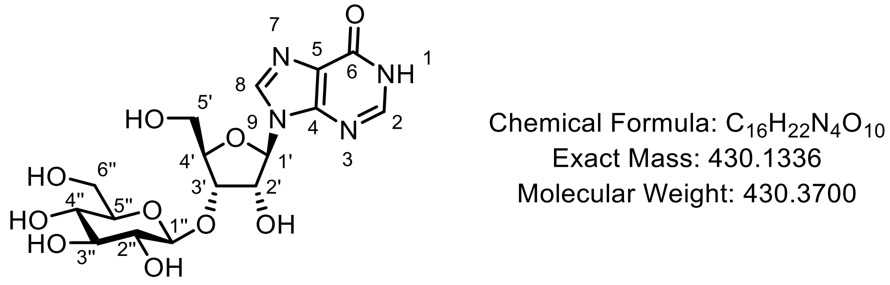

Method A; Yield: 9.3 mg, 86%; **R_f** 0.42 (3:2 IPA/35% aq. NH_4OH); **^{1}H NMR** (400 MHz; D_2O) δ 8.33 (1 H, s, **H_8**), 8.20 (1 H, s, **H_2**), 6.12 (1 H, d, *J* = 5.3 Hz, **$H_{1'}$**), 4.87 (1 H, t, *J* = 5.3 Hz, **$H_{2'}$**), 4.63 (1 H, d, *J* = 4.5 Hz, **$H_{3'}$**), 4.62 (1 H, d, *J* = 7.6 Hz, **$H_{1''}$**), 4.42 (1 H, q, *J* = 4.1 Hz, **$H_{4'}$**), 3.94 (1 H, dd, *J* = 12.8, 3.1 Hz, **$H_{6''}$**), 3.90 (1 H, dd, *J* = 12.4, 2.0 Hz, **$H_{5'}$**), 3.84 (1 H, dd, *J* = 12.9, 4.0 Hz, **$H_{6''}$**), 3.74 (1 H, dd, *J* = 12.4, 5.3 Hz, **$H_{5'}$**), 3.56–3.39 (4 H, m, **$H_{2''}$**, **$H_{3''}$**, **$H_{4''}$**, **$H_{5''}$**); **^{13}C NMR** (100 MHz; D_2O) δ 158.5 (**C_6**), 148.4 (**C_4**), 146.1 (**C_2**), 140.2 (**C_8**), 124.2 (**C_5**), 102.0 (**$C_{1''}$**), 88.5 (**$C_{1'}$**), 83.7 (**$C_{4'}$**), 77.7 (**$C_{3'}$**), 75.9 (**$C_{5''}$**), 75.5 (**$C_{3''}$**), 73.6 (**$C_{2'}$**), 72.8 (**$C_{2''}$**), 69.4 (**$C_{4''}$**), 61.0 (**$C_{6''}$**), 60.5 (**$C_{5'}$**); **HRMS** [ES+] found $[M+H]^+$ 431.1403, $C_{16}H_{23}N_4O_{10}$ requires 431.3775, error −1.39 ppm.

9-(3′-*O*-Glucosyl-β-ᴅ-arabinofuranosyl)adenine (5a)

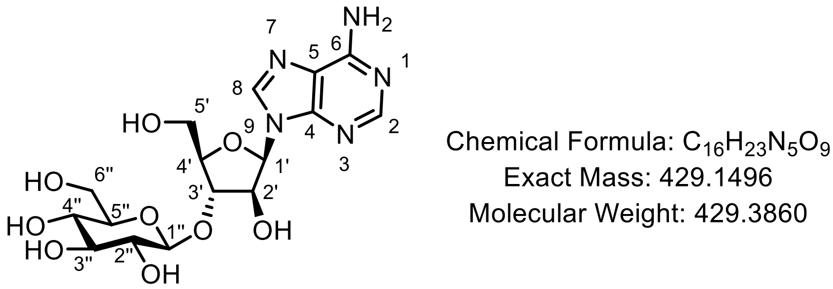

Method A; Yield: 9.8 mg, 91%; **R_f** 0.62 (3:2 IPA/35% aq. NH_4OH); **^{1}H NMR** (400 MHz; D_2O) δ 8.33 (1 H, s, **H_8**), 8.15 (1 H, s, **H_2**), 6.38 (1 H, d, *J* = 5.5 Hz, **$H_{1'}$**), 4.74 (1 H, t, *J* = 5.1 Hz, **$H_{2'}$**), 4.61 (1 H, d, *J* = 8.0 Hz, **$H_{1''}$**), 4.47 (1 H, dd, *J* = 5.8, 4.8 Hz, **$H_{3'}$**), 4.24 (1 H, dq, *J* = 5.8, 3.5 Hz, **$H_{4'}$**), 3.99 (1 H, dd, *J* = 12.5, 3.4 Hz, **$H_{5a'}$**), 3.94–3.88 (2 H, m, **$H_{5b'}$**, **$H_{6a''}$**), 3.70 (1 H, dd, *J* = 12.5, 6.3

Hz, **H6b″**), 3.53 (1 H, t, *J* = 9.0 Hz, **H3″**), 3.50 (1 H, ddd, *J* = 9.8, 6.3, 2.3 Hz, **H5″**), 3.41 (1 H, dd, *J* = 9.8, 8.9 Hz, **H4″**), 3.37 (1 H, dd, *J* = 9.4, 7.9 Hz, **H2″**); **^{13}C NMR** (100 MHz; D_2O) δ 155.1 (**C6**), 152.2 (**C2**), 148.5 (**C4**), 141.2 (**C8**), 118.1 (**C5**), 102.5 (**C1″**), 84.3 (**C1′**), 83.1 (**C3′**), 81.8 (**C4′**), 76.0 (**C5″**), 75.5 (**C3″**), 74.6 (**C2′**), 73.0 (**C2″**), 69.7 (**C4″**), 60.7 (**C6″**), 60.6 (**C5′**); **HRMS** [ES+] found $[M+H]^+$ 430.1559, $C_{16}H_{24}N_5O_9$ requires 430.1569, error −2.32 ppm.

3′-*O*-β-Glucosyluridine (11a)

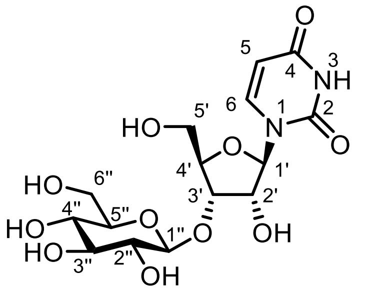

Method A; Yield: 7.3 mg, 72%; **R_f** 0.38 (3:2 IPA/35% aq. NH_4OH); **^{1}H NMR** (400 MHz; D_2O) δ 7.72 (1 H, d, *J* = 8.0 Hz, **H6**), 5.76 (1 H, d, *J* = 4.1 Hz, **H1′**), 5.73 (1 H, d, *J* = 8.1 Hz, **H5**), 4.40 (1 H, d, *J* = 7.8 Hz, **H1″**), 4.32 (1 H, dd, *J* = 5.2, 4.1 Hz, **H2′**), 4.22 (1 H, t, *J* = 5.7 Hz, **H3′**), 4.10 (1 H, ddd, *J* = 7.2, 4.1, 2.9 Hz, **H4′**), 3.78 (1 H, dd, *J* = 12.9, 3.0 Hz, **H5a′**), 3.74 (1 H, dd, *J* = 12.1, 1.5 Hz, **H6a″**), 3.66 (1 H, dd, *J* = 12.9, 4.1 Hz, **H5b′**), 3.58 (1 H, dd, *J* = 12.4, 5.1 Hz, **H6b″**), 3.38–3.26 (3 H, m, **H3″**, **H4″**, **H5″**), 3.22 (1 H, dd, *J* = 9.3, 7.8 Hz, **H2″**); **^{13}C NMR** (100 MHz; D_2O) δ 166.2 (**C4**), 151.6 (**C2**), 141.8 (**C6**), 102.3 (**C1″**), 102.3 (**C5**), 89.7 (**C1′**), 82.5 (**C4′**), 77.0 (**C3′**), 75.9 (**C5″**), 75.4 (**C3″**), 73.3 (**C2′**), 72.7 (**C2″**), 69.4 (**C4″**), 60.5 (**C6″**), 60.4 (**C5′**); **HRMS** [ES+] found $[M+Na]^+$ 429.1116, $C_{15}H_{22}N_2O_{11}Na$ requires 429.1116, error 0.00 ppm.

3′-*O*-β-Glucosylcytidine (12a)

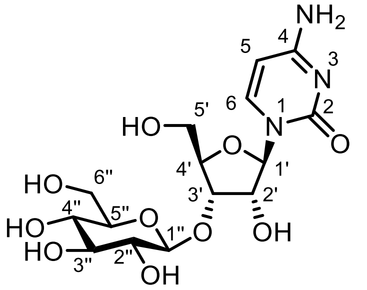

Method A; Yield: 6.9 mg, 68%; **R_f** 0.36 (3:2 IPA/35% aq. NH_4OH); **^{1}H NMR** (400 MHz; D_2O) δ 7.95 (1 H, d, *J* = 7.8 Hz, **H6**), 6.08 (1 H, d, *J* = 6.8 Hz, **H5**), 5.84 (1 H, d, *J* = 3.3 Hz, **H1′**), 4.47 (1 H, d, *J* = 7.8 Hz, **H1″**), 4.39 (1 H, dd, *J* = 5.1, 3.5 Hz, **H2′**), 4.27 (1 H, t, *J* = 6.3 Hz, **H3′**), 4.21 (1 H, dd, *J* = 6.7, 4.0 Hz, **H4′**), 3.90 (1 H, dd, *J* = 12.9, 3.0 Hz, **H5a′**), 3.82 (1 H, dd, *J* = 12.5, 1.8 Hz, **H6a″**), 3.77 (1 H, dd, *J* = 12.9, 3.8 Hz, **H5b′**), 3.67 (1 H, dd, *J* = 11.4, 5.1 Hz, **H6b″**), 3.45–3.34 (3 H, m, **H3″**, **H4″**, **H5″**), 3.30 (1 H, dd, *J* = 8.9, 7.8 Hz, **H2″**); **^{13}C NMR** (100 MHz; D_2O) δ 162.4 (**C4**), 152.5 (**C2**), 143.0 (**C6**), 102.5 (**C1″**), 95.5 (**C5**), 90.5 (**C1′**), 82.4 (**C4′**), 76.9 (**C3′**), 75.9 (**C5″**), 75.4 (**C3″**), 73.6 (**C2′**), 72.7 (**C2″**), 69.4 (**C4″**), 60.5 (**C6″**), 60.2 (**C5′**); **HRMS** [ES+] found $[M+H]^+$ 406.1458, $C_{15}H_{24}N_3O_{10}$ requires 406.1456, error +0.49 ppm.

3′-*O*-β-Glucosyl-6-*O*-methylguanosine (16a)

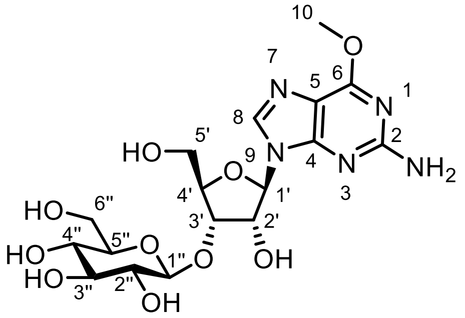

Chemical Formula: $C_{17}H_{25}N_5O_{10}$
Exact Mass: 459.1601
Molecular Weight: 459.4120

Method A; Yield: 10.2 mg, 88%; **R$_f$** 0.57 (3:2 IPA/35% aq. NH_4OH); **^{1}H NMR** (400 MHz; D_2O) δ 7.98 (1 H, s, **H_8**), 5.91 (1 H, d, *J* = 5.5 Hz, **$H_{1'}$**), 4.85 (1 H, t$_{app}$, *J* = 5.7 Hz, **$H_{2'}$**), 4.62 (1 H, d, *J* = 8.5 Hz, **$H_{1''}$**), 4.59 (1 H, d, *J* = 4.8 Hz, **$H_{3'}$**), 4.39 (1 H, q, *J* = 6.3, 3.5 Hz, **$H_{4'}$**), 3.99 (3 H, s, OMe), 3.94 (1 H, dd, *J* = 7.8, 2.9 Hz, **$H_{5a'}$**), 3.91 (1 H, dd, *J* = 7.2, 2.7 Hz, **$H_{6a''}$**), 3.83 (1 H, dd, *J* = 12.7, 3.5 Hz, **$H_{5b''}$**), 3.76 (1 H, dd, *J* = 12.3, 4.8 Hz, **$H_{6b''}$**), 3.57–3.39 (4 H, m, **$H_{2''}$**, **$H_{3''}$**, **$H_{4''}$**, **$H_{5''}$**); **^{13}C NMR** (100 MHz; D_2O) δ 161.2 (**C_2**), 159.6 (**C_4**), 152.2 (**C_5**), 138.9 (**C_8**), 114.4 (**C_6**), 101.7 (**$C_{1''}$**), 88.3 (**$C_{1'}$**), 83.8 (**$C_{4'}$**), 77.9 (**$C_{3'}$**), 75.9 (**$C_{5''}$**), 75.5 (**$C_{3''}$**), 72.8 (**$C_{2'}$**, **$C_{2''}$**), 69.4 (**$C_{4''}$**), 61.4 (**$C_{5'}$**), 60.5 (**$C_{6''}$**), 54.3 (**C_{10}**); **HRMS** [ES+] found $[M+H]^+$ 460.1663, $C_{17}H_{26}N_5O_{10}$ requires 460.1674, error −2.39 ppm.

6-Methoxy-9-(3′-*O*-β-glucosyl-β-D-arabinofuranosyl)guanine (3′-*O*-β-glucosylnelarabine) (17a)

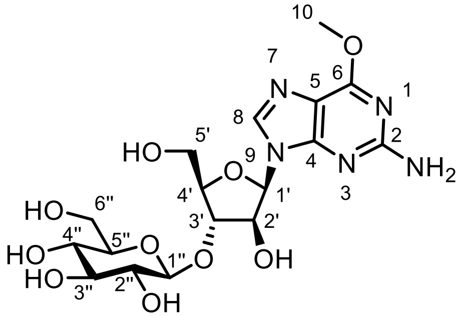

Chemical Formula: $C_{17}H_{25}N_5O_{10}$
Exact Mass: 459.1601
Molecular Weight: 459.4120

Method A; Yield: 10.1 mg, 88%; **R$_f$** 0.62 (3:2 IPA/35% aq. NH_4OH); **^{1}H NMR** (400 MHz; D_2O) δ 8.06 (1 H, s, **H_8**), 6.20 (1 H, d, *J* = 5.2 Hz, **$H_{1'}$**), 4.70 (1 H, t$_{app}$, *J* = 4.8 Hz, **$H_{2'}$**), 4.60 (1 H, d, *J* = 7.9 Hz, **$H_{1''}$**), 4.44 (1 H, t$_{app}$, *J* = 4.9 Hz, **$H_{3'}$**), 4.20 (1 H, q, *J* = 8.9, 4.7 Hz, **$H_{4'}$**), 4.01 (3 H, s, OMe), 3.96–3.87 (3 H, m, **$H_{5a,b'}$**, **$H_{6a''}$**), 3.71 (1 H, dd, *J* = 12.4, 6.3 Hz, **$H_{6b''}$**), 3.54 (1 H, t$_{app}$, *J* = 9.1 Hz, **$H_{3''}$**), 3.51 (1 H, ddd, *J* = 9.5, 6.2, 2.0 Hz, **$H_{5''}$**), 3.42 (1 H, d, *J* = 9.2 Hz, **$H_{4''}$**), 3.38 (1 H, d, *J* = 8.6 Hz, **$H_{2''}$**); **^{13}C NMR** (100 MHz; D_2O) δ 161.2 (**C_2**), 159.8 (**C_4, C_5**), 139.5 (**C_8**), 102.5 (**$C_{1''}$**), 84.0 (**$C_{1'}$**), 83.4 (**$C_{3'}$**), 81.6 (**$C_{4'}$**), 76.0 (**$C_{5''}$**), 75.5 (**$C_{3''}$**), 74.5 (**$C_{2'}$**), 73.0 (**$C_{2''}$**), 69.7 (**$C_{4''}$**), 60.7 (**$C_{5'}$**, **$C_{6''}$**), 54.3 (**C_{10}**); **HRMS** [ES+] found $[M+H]^+$ 460.1661, $C_{17}H_{27}N_5O_{10}$ requires 460.1674, error −2.83 ppm.

2-Fluoro-9-(3′-*O*-β-glucosyl-β-D-arabinofuranosyl)adenine (3′-*O*-β-glucosylfludarabine) (18a)

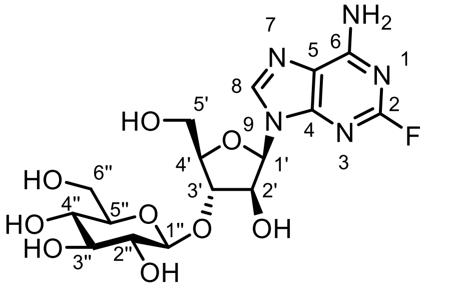

Chemical Formula: $C_{16}H_{22}FN_5O_9$
Exact Mass: 447.1402
Molecular Weight: 447.3764

Method B; Yield: 7.2 mg, 64%; $\mathbf{R_f}$ 0.60 (3:2 IPA/35% aq. NH_4OH); **^{1}H NMR** (400 MHz; D_2O) δ 8.27 (1 H, s, $\mathbf{H_8}$), 6.28 (1 H, d, *J* = 5.4 Hz, $\mathbf{H_{1'}}$), 4.74 (1 H, t, *J* = 5.2 Hz, $\mathbf{H_{2'}}$), 4.61 (1 H, d, *J* = 7.7 Hz, $\mathbf{H_{1''}}$), 4.48 (1 H, t, *J* = 5.4 Hz, $\mathbf{H_{3'}}$), 4.23 (1 H, q, *J* = 8.9, 5.2 Hz, $\mathbf{H_{4'}}$), 4.01–3.89 (3 H, m, $\mathbf{H_{5a,b'}}$, $\mathbf{H_{6a''}}$), 3.71 (1 H, dd, *J* = 12.5, 6.3 Hz, $\mathbf{H_{6b''}}$), 3.54 (1 H, t, *J* = 9.1 Hz, $\mathbf{H_{4''}}$), 3.50 (1 H, dt, *J* = 6.8 Hz, 2.3 Hz, $\mathbf{H_{5''}}$), 3.44–3.33 (2 H, m, $\mathbf{H_{2''}}$, $\mathbf{H_{3''}}$); **^{13}C NMR** (100 MHz; D_2O) δ 158.8 (d, $^1J_{C\text{-}F}$ = 211 Hz, $\mathbf{C_2}$), 157.1 ($\mathbf{C_6}$), 156.9 ($\mathbf{C_4}$), 141.2 ($\mathbf{C_8}$), 116.5 ($\mathbf{C_5}$), 102.5 ($\mathbf{C_{1''}}$), 84.3 ($\mathbf{C_{1'}}$), 83.0 ($\mathbf{C_{3'}}$), 81.6 ($\mathbf{C_{4'}}$), 76.0 ($\mathbf{C_{4''}}$), 75.5 ($\mathbf{C_{5''}}$), 74.5 ($\mathbf{C_{2'}}$), 73.0 ($\mathbf{C_{2''}}$), 69.7 ($\mathbf{C_{3''}}$), 60.7 ($\mathbf{C_{6''}}$), 60.5 ($\mathbf{C_{5'}}$); **^{19}F NMR** (376 MHz, D_2O) δ −53.1; **HRMS** [ES+] found $[M+H]^+$ 448.1471, $C_{16}H_{23}FN_5O_9$ requires 448.1474, error −0.67 ppm.

2-Chloro-9-(3′-*O*-β-glucosyl-2'-deoxy-2'-fluoro-β-D-arabinofuranosyl)adenine (3′-*O*-β-glucosyl-clofarabine) (19a)

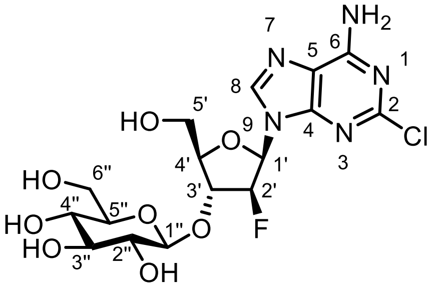

Chemical Formula: $C_{16}H_{21}ClFN_5O_8$
Exact Mass: 465.1063
Molecular Weight: 465.8194

Method B; Yield: 4.5 mg, 39%; $\mathbf{R_f}$ 0.63 (3:2 IPA/35% aq. NH_4OH); **^{1}H NMR** (400 MHz; D_2O) δ 8.26 (1 H, d, $^4J_{H8\text{-}F}$ = 2.0 Hz, $\mathbf{H_8}$), 6.37 (1 H, dd, $^3J_{H1'\text{-}F}$ = 18.4 Hz, $^3J_{H1'\text{-}H2'}$ = 4.0 Hz, $\mathbf{H_{1'}}$), 5.54 (1 H, dt, $^1J_{H2'\text{-}F}$ = 50.7 Hz, $^3J_{H2'\text{-}H1'}$ = 3.0 Hz, $^3J_{H2'\text{-}H3'}$ = 3.0 Hz, $\mathbf{H_{2'}}$), 4.71 (1 H, ddd, $^1J_{H3'\text{-}F}$ = 18.0 Hz, $^3J_{H3'\text{-}H4'}$ = 4.4 Hz, $^3J_{H3'\text{-}H2'}$ = 2.6 Hz, $\mathbf{H_{3'}}$), 4.67 (1 H, d, $^3J_{H1''\text{-}H2''}$ = 7.8 Hz, $\mathbf{H_{1''}}$), 4.30 (1 H, q, $^3J_{H4'\text{-}H3a'}$ = 4.4 Hz, $^3J_{H4'\text{-}H5a/b'}$ = 4.4 Hz, $\mathbf{H_{4'}}$), 4.00–3.87 (3 H, m, $\mathbf{H_{5a'}}$, $\mathbf{H_{5b'}}$, $\mathbf{H_{6a''}}$), 3.75 (1 H, dd, $^2J_{H6b''\text{-}H6a''}$ = 12.2 Hz, $^3J_{H6b''\text{-}H5''}$ = 5.2 Hz, $\mathbf{H_{6b''}}$), 3.58–3.44 (3 H, m, $\mathbf{H_{3''}}$, $\mathbf{H_{4''}}$, $\mathbf{H_{5''}}$), 3.38 (1 H, t, $^3J_{H2''\text{-}H1''}$ = 7.8 Hz, $^3J_{H2''\text{-}H3''}$ = 7.8 Hz, $\mathbf{H_{2''}}$); **^{13}C NMR** (100 MHz; D_2O) δ 156.1 ($\mathbf{C_2}$), 153.8 ($\mathbf{C_6}$), 149.7 ($\mathbf{C_4}$), 141.2 (d, $^4J_{C8\text{-}F}$ = 6.6 Hz, $\mathbf{C_8}$), 117.0 ($\mathbf{C_5}$), 102.6 ($\mathbf{C_{1''}}$), 93.7 (d, $^1J_{C2'\text{-}F}$ = 192.0 Hz, $\mathbf{C_{2'}}$), 83.0 (d, $^2J_{C1'\text{-}F}$ = 16.7 Hz, $\mathbf{C_{1'}}$), 82.4 (d, $^3J_{C4'\text{-}F}$ = 3.5 Hz, $\mathbf{C_{4'}}$), 81.3 (d, $^2J_{C3'\text{-}F}$ = 26.5 Hz, $\mathbf{C_{3'}}$),76.0 ($\mathbf{C_{4''}}$), 75.5 ($\mathbf{C_{5''}}$), 72.9 ($\mathbf{C_{2''}}$), 69.4 ($\mathbf{C_{3''}}$), 60.7 ($\mathbf{C_{5'}}$), 60.4 ($\mathbf{C_{6''}}$); **^{19}F NMR** (376 MHz, D_2O) δ −197.9 (dt, $^1J_{F\text{-}H2'}$ = 51.2 Hz, $^3J_{F\text{-}H1'}$ = 18.3 Hz, $^3J_{F\text{-}H3'}$ = 18.3 Hz); **HRMS** [ES+] found $[M+H]^+$ 466.1132, $C_{16}H_{22}ClFN_5O_8$ requires 466.1135, error −0.64 ppm.

N^6-Propargyladenosine (15)

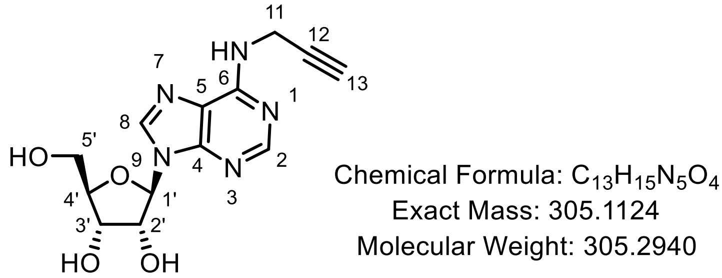

Chemical Formula: $C_{13}H_{15}N_5O_4$
Exact Mass: 305.1124
Molecular Weight: 305.2940

6-chloropurine riboside (429 mg, 1.5 mmol, 1 eq) and 2-propargylamine (288 µL, 4.5 mmol, 3 eq) were dissolved in absolute EtOH (10 mL) and Et_3N (627 µL, 4.5 mmol, 3 eq) and heated at reflux (80 °C) for 3 hours. The solution was concentrated *in vacuo* and the resulting residue purified by preparative RP-HPLC using Polaris 5 C18-A 250 × 21.2mm using water+0.1% formic acid (A) and MeOH (B) according to the following scheme: 5-60%B gradient over 16 mins, gradient to 100%B over 1 min, hold at 100%B for 6 minutes, 96-5%B gradient over 2 mins, hold at 5%B for 6 minutes. Flow rate 20 mL/min, Injection volume 500 µL, sample concentration 50 mg/mL, UV detection at 260 nm. Yield: 411 mg (90%).

R$_f$ 0.70 (3:2 IPA/35% aq. NH_4OH); **^{1}H NMR** (400 MHz; D_2O) δ 8.16 (1 H, s, **H$_8$**), 8.05 (1 H, s, **H$_2$**), 5.93 (1 H, d, *J* = 6.1 Hz, **H$_{1'}$**), 4.68 (1 H, t, *J* = 5.6 Hz, **H$_{2'}$**), 4.39 (1 H, t$_{app}$, *J* = 3.9 Hz, **H$_{3'}$**), 4.26 (1 H, q, *J* = 3.3 Hz, **H$_{4'}$**), 4.18 (2 H, br.s, **H$_{11}$**), 3.90 (1 H, dd, *J* = 12.8, 2.5 Hz, **H$_{5a'}$**), 3.81 (1 H, dd, *J* = 12.8, 3.4 Hz, **H$_{5b'}$**), 2.64 (1 H, t, *J* = 2.3 Hz, **H$_{13}$**); **^{13}C NMR** (100 MHz; D_2O) δ 153.3 (**C$_6$**), 151.9 (**C$_2$**), 147.4 (**C$_4$**), 140.2 (**C$_8$**), 119.1 (**C$_5$**), 88.4 (**C$_{1'}$**), 85.7 (**C$_{4'}$**), 80.0 (**C$_{12}$**), 73.8 (**C$_{2'}$**), 72.1 (**C$_{13}$**), 70.6 (**C$_{3'}$**), 61.5 (**C$_5$**), 30.1 (**C$_{11}$**); **HRMS** [ES+] found $[M+H]^+$ 306.1197, $C_{13}H_{16}N_5O_4$ requires 306.1197, error 0.00 ppm. Data were in agreement with published literature.[2]

3'-*O*-β-Glucosyl-*N*^{6}-propargyladenosine (15a)

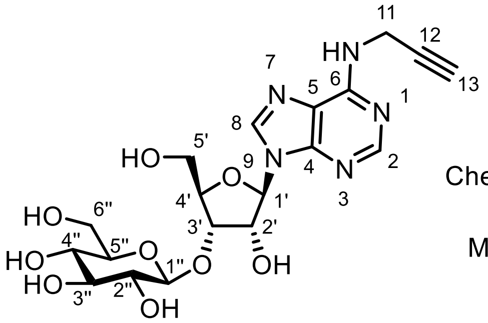

Chemical Formula: $C_{19}H_{25}N_5O_9$
Exact Mass: 467.1652
Molecular Weight: 467.4350

A 50 mL centrifuge tube was charged with *N*^{6}-propargyladenosine (30.5 mg, 100 µmol, 10 mM, 1.0 eq) and UDP-Glucose ($2Na^+$) (91.5 mg, 150 µmol, 15 mM, 1.5 eq) then suspended in 8.214 mL of deionised H_2O. The reaction was made up first with 1 mL of a 10× stock of the reaction buffer (stock conc.: 0.5 M Tris (pH7.5), 1 M NaCl, 10 mM DTT; final conc.: 50 mM Tris (pH7.5), 100 mM NaCl, 1 mM DTT), 100 µL of $MgCl_2$ (stock conc. 1 M; final conc. 10 mM) and finally 286 µL of AvpGT (stock conc. 350 µM; final conc. 10 µM, 0.1 mol%). The reactions were incubated at 30 °C for 16 hrs with gentle shaking (150 rpm). The reactions were monitored by TLC (3:2 IPA/35% aq. NH_4OH). Once they had reached completion, ice cold ethanol (10 mL) was added, and the reaction placed in a –20 °C freezer for 4 hours to precipitate the enzyme. The supernatants were first concentrated *in vacuo*, then lyophilised before being purified by preparative RP-HPLC using Polaris 5 C18-A 250 × 21.2mm using water+0.1% formic acid (A) and MeOH (B) according to the following scheme: 5-60%B gradient over 16 mins, gradient to

100%B over 1 min, hold at 100%B for 6 minutes, 96-5%B gradient over 2 mins, hold at 5%B for 6 minutes. Flow rate 20 mL/min, Injection volume 500 µL, sample concentration 50 mg/mL, UV detection at 260 nm.

Yield: 41.2 mg, 88%; $\mathbf{R_f}$ 0.59 (3:2 IPA/35% aq. NH_4OH); **^{1}H NMR** (400 MHz; D_2O) δ 8.17 (1 H, s, $\mathbf{H_8}$), 8.06 (1 H, s, $\mathbf{H_2}$), 5.98 (1 H, d, *J* = 5.7 Hz, $\mathbf{H_{1'}}$), 4.81 (1 H, d, *J* = 5.6 Hz, $\mathbf{H_{2'}}$), 4.62 (1 H, d, *J* = 7.8 Hz, $\mathbf{H_{1''}}$), 4.58 (1 H, dd, *J* = 5.2, 3.8 Hz, $\mathbf{H_{3'}}$), 4.41 (1 H, q, *J* = 3.4 Hz, $\mathbf{H_{4'}}$), 4.19 (1 H, br. s, $\mathbf{H_{11}}$), 3.94 (1 H, dd, *J* = 13.1, 3.0 Hz, $\mathbf{H_{5a'}}$), 3.90 (1 H, dd, *J* = 12.2, 1.8 Hz, $\mathbf{H_{6a''}}$), 3.83 (1 H, dd, *J* = 13.0, 3.4 Hz, $\mathbf{H_{5b'}}$), 3.74 (1 H, dd, *J* = 12.4, 5.0 Hz, $\mathbf{H_{6b''}}$), 3.56–3.39 (4 H, m, $\mathbf{H_{2''}}$, $\mathbf{H_{3''}}$, $\mathbf{H_{4''}}$, $\mathbf{H_{5''}}$,), 2.71 (1 H, s, $\mathbf{H_{13}}$); **^{13}C NMR** (100 MHz; D_2O) δ153.4 ($\mathbf{C_6}$), 152.1 ($\mathbf{C_2}$), 147.4 ($\mathbf{C_4}$), 140.1 ($\mathbf{C_8}$), 119.1 ($\mathbf{C_5}$), 101.8 ($\mathbf{C_{1''}}$), 88.4 ($\mathbf{C_{1'}}$), 83.9 ($\mathbf{C_{4'}}$), 79.6 ($\mathbf{C_{12}}$), 77.8 ($\mathbf{C_3}$), 75.9 ($\mathbf{C_{4''}}$), 75.5 ($\mathbf{C_{3''}}$), 73.2 ($\mathbf{C_{2'}}$), 72.8 ($\mathbf{C_{2''}}$), 69.4 ($\mathbf{C_{5''}}$),61.3 ($\mathbf{C_{5'}}$), 60.5 ($\mathbf{C_{6''}}$), 38.7 ($\mathbf{C_{13}}$), 30.1 ($\mathbf{C_{11}}$); **HRMS** [ES+] found $[M+H]^+$ 468.1719, $C_{19}H_{26}N_5O_9$ requires 468.1725, error −1.28 ppm.

References

(1) Pasternak, A. R. O.; Balunas, M. J.; Zechel, D. L. Discovery of 3′-O-β-Glucosyltubercidin and the Nucleoside Specific Glycosyltransferase AvpGT through Genome Mining. *ACS Chem Biol* **2022**, *17* (12), 3507–3514. https://doi.org/10.1021/acschembio.2c00707.

(2) Gao, X.; Shu, X.; Song, Y.; Cao, J.; Gao, M.; Wang, F.; Wang, Y.; Sun, J. Z.; Liu, J.; Tang, B. Z. Visualization and Quantification of Cellular RNA Production and Degradation Using a Combined Fluorescence and Mass Spectrometry Characterization Assay. *Chemical Communications* **2019**, *55* (57), 8321–8324. https://doi.org/10.1039/C9CC03923F.

NMR Spectra

3’-O-β-Glucosyladenosine (1a)

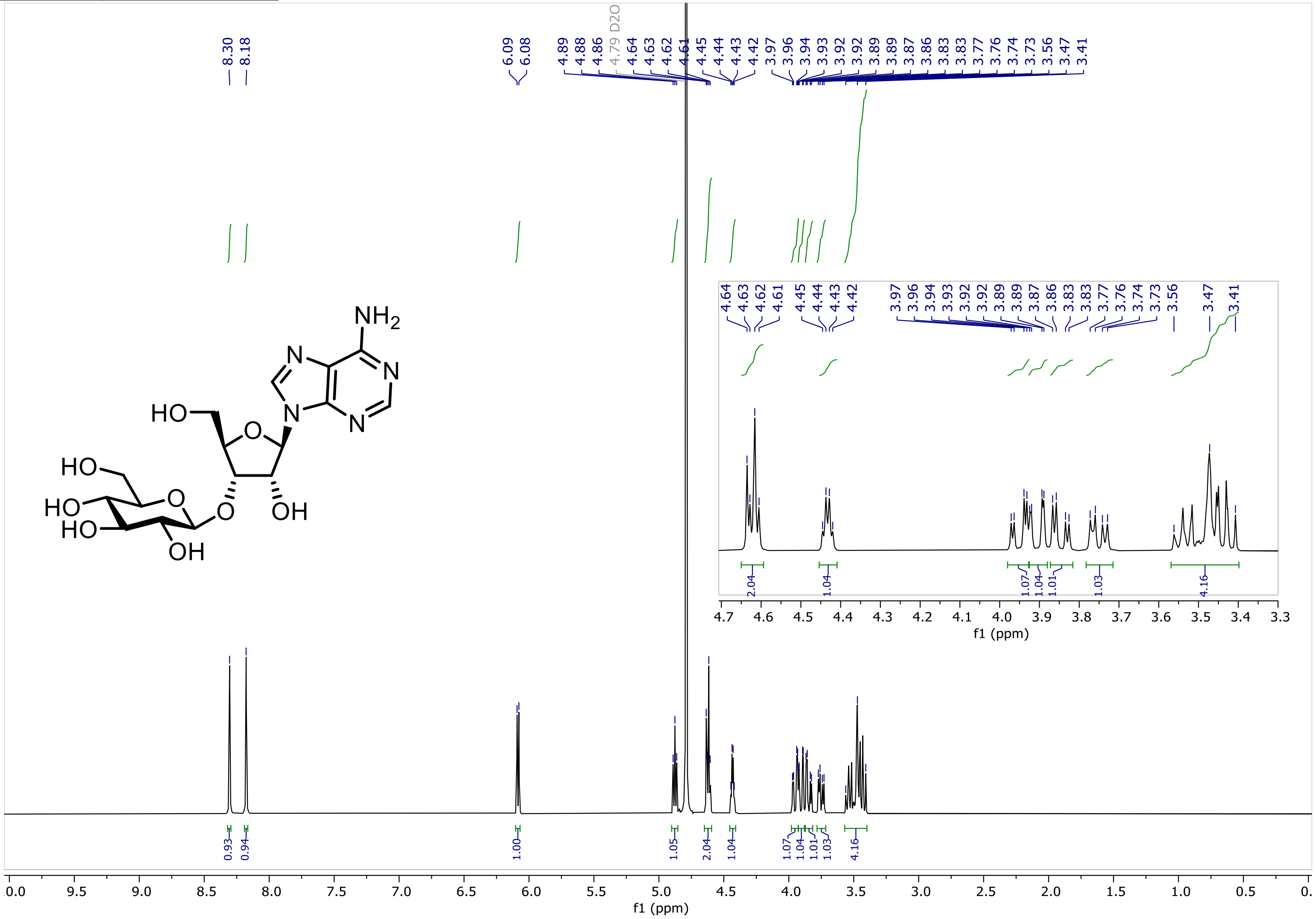

^{1}H NMR

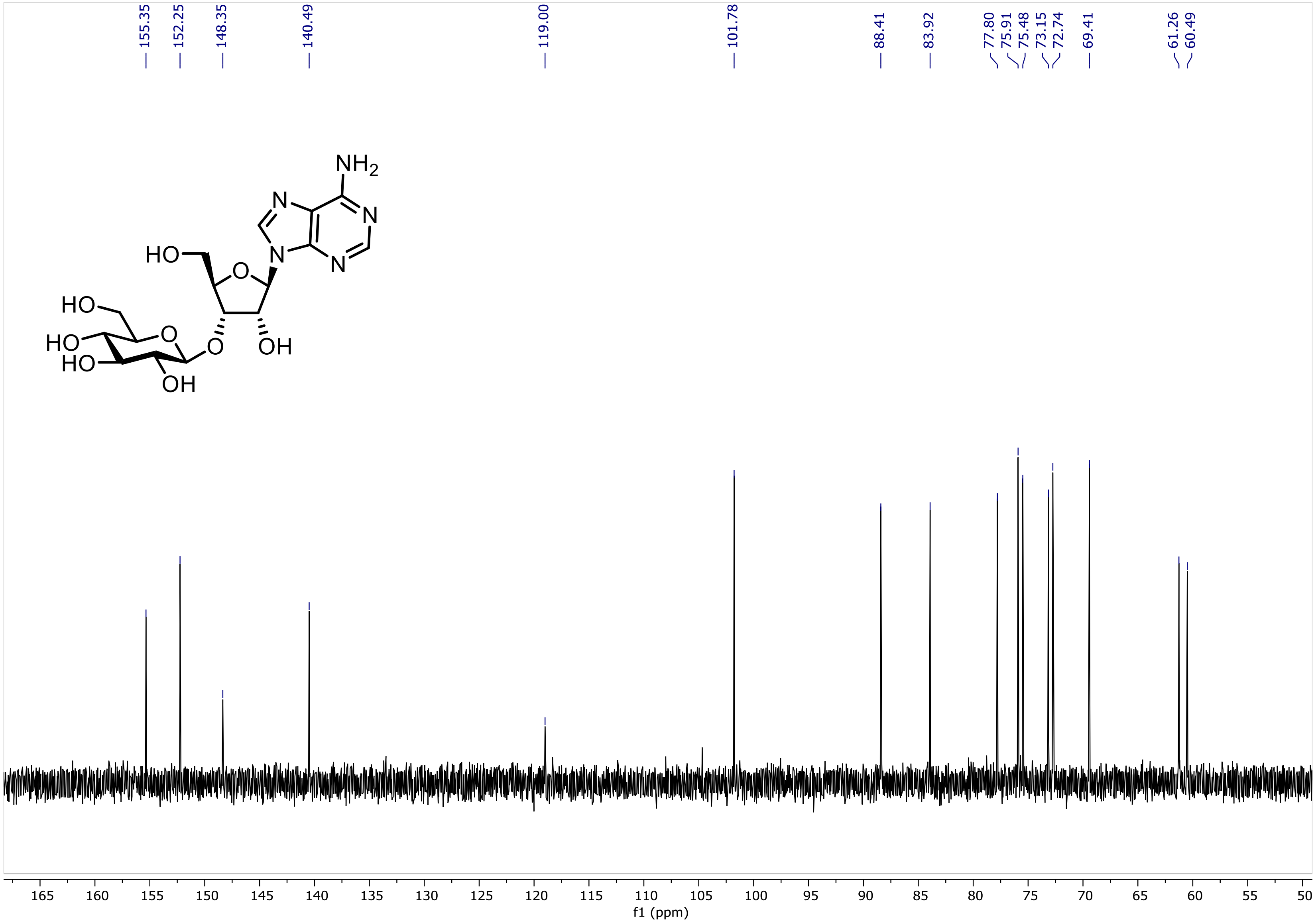

155.35
152.25
148.35
140.49
119.00
101.78
88.41
83.92
77.80
75.91
75.48
73.15
72.74
69.41
61.26
60.49
NH2
N
N
N
N
HO
O
HO
O
HO
O
HO
OH
OH
165 160 155 150 145 140 135 130 125 120 115 110 105 100 95 90 85 80 75 70 65 60 55 50
f1 (ppm)

^{13}C NMR

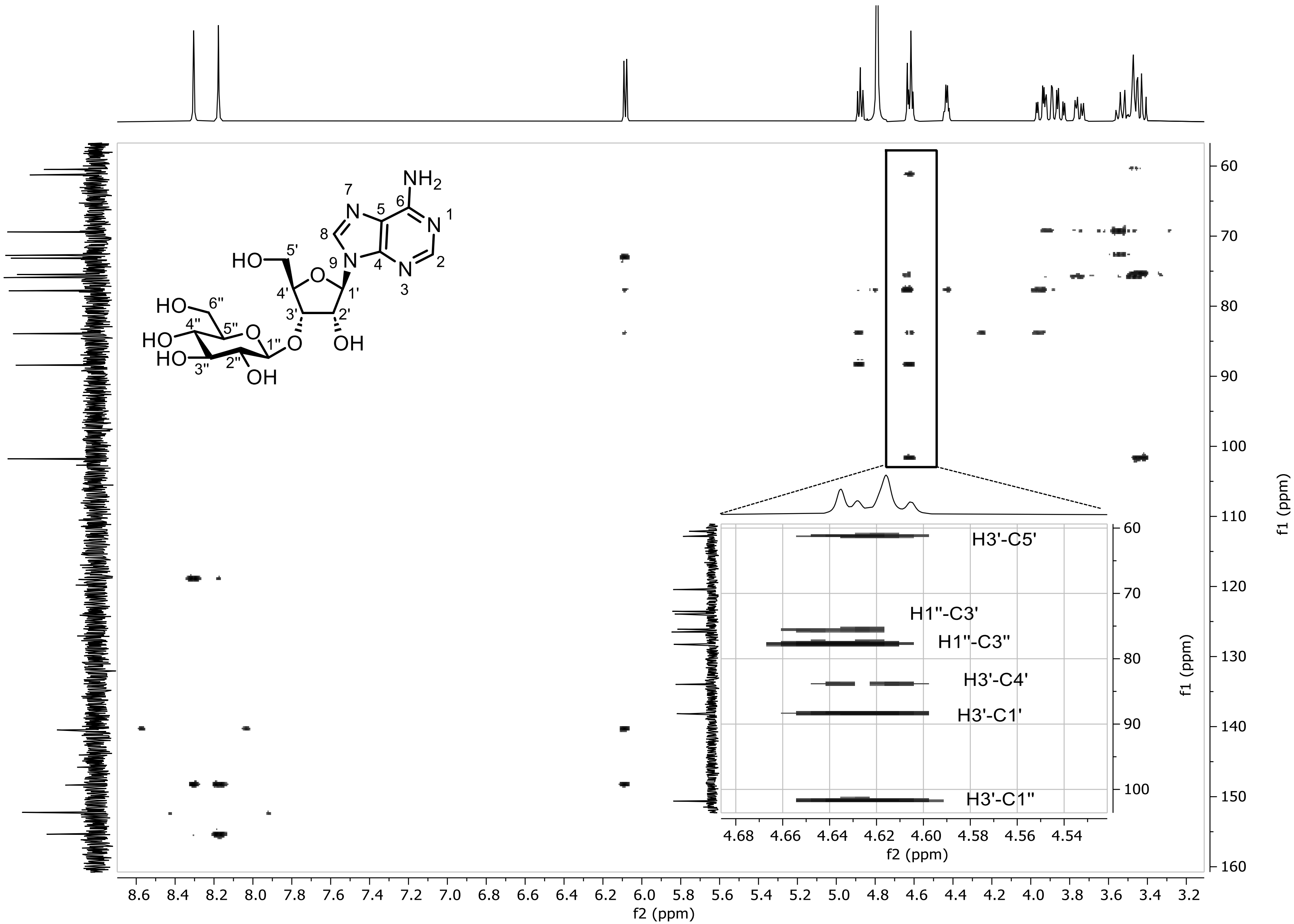
NH2
N
N
N
N
1
2
3
4
5
6
7
8
9
HO
5'
O
1'
2'
3'
4'
OH
O
HO
6''
4''
5''
1''
2''
3''
HO
HO
OH
H3'-C5'
H1''-C3'
H1''-C3''
H3'-C4'
H3'-C1'
H3'-C1''
f1 (ppm)
f2 (ppm)
f2 (ppm)
f1 (ppm)

^{1}H-^{13}C HMBC

3’-O-β-Glucosyltubercidin (2a)

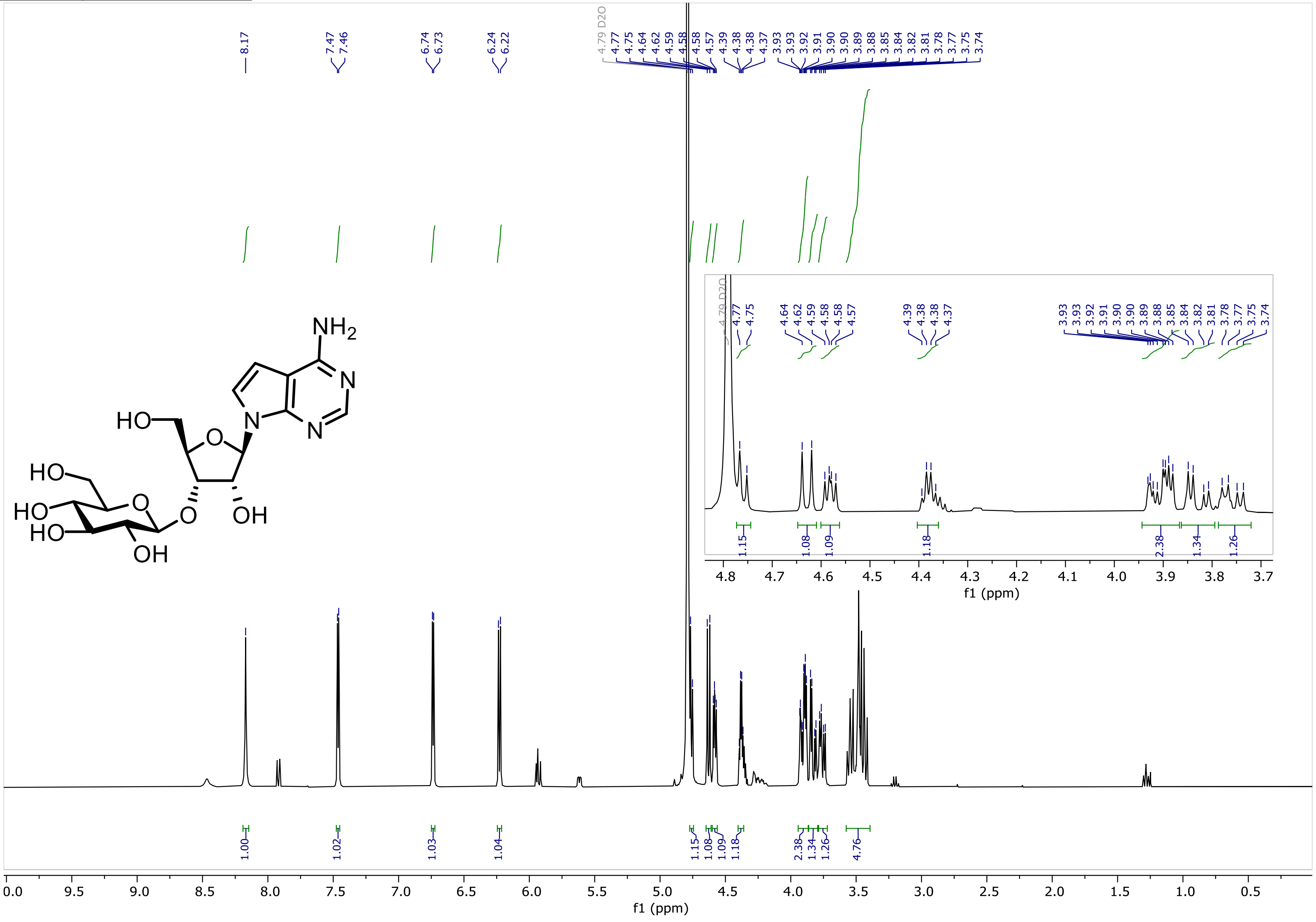

^{1}H NMR

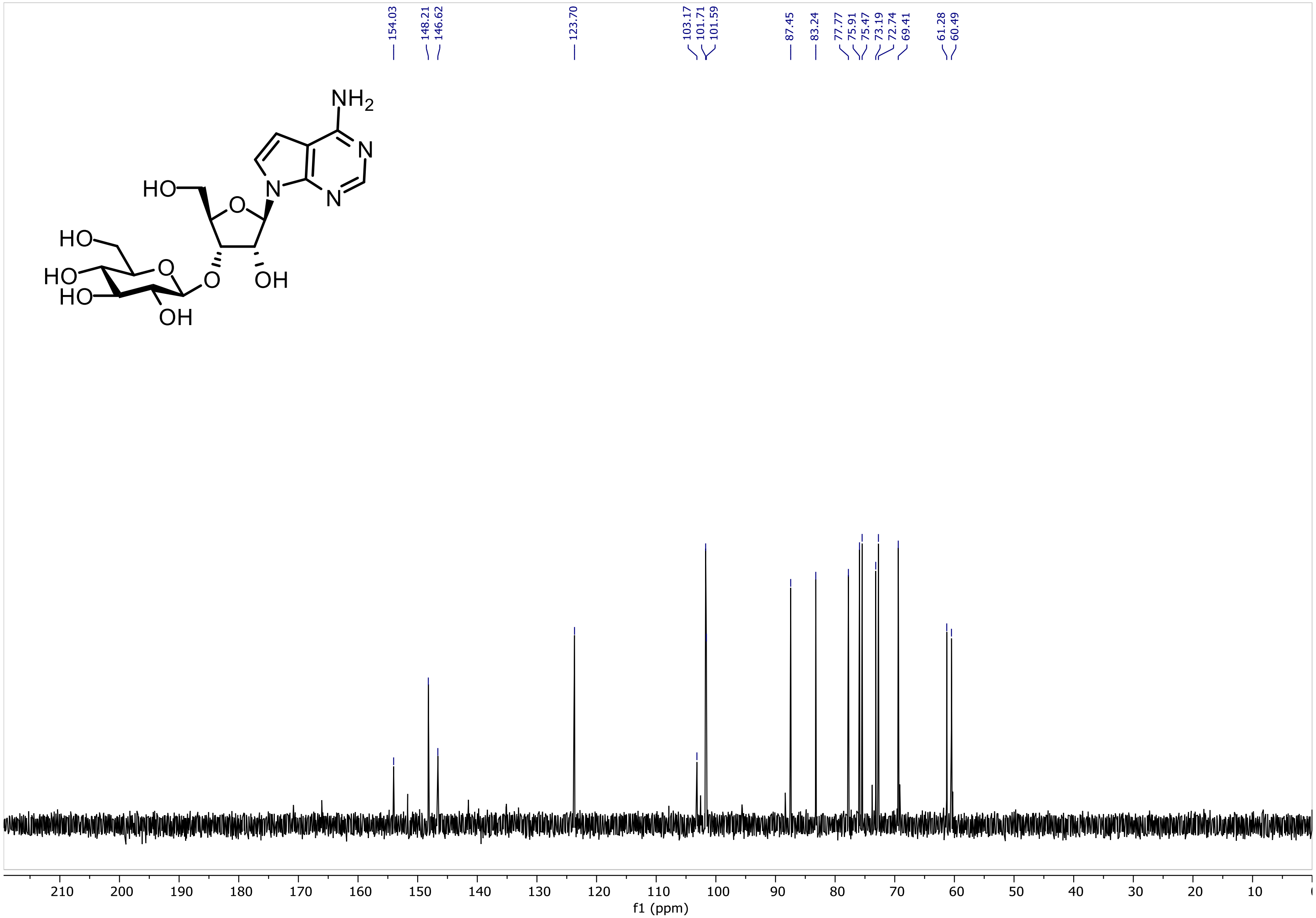

154.03
148.21
146.62
123.70
103.17
101.71
101.59
87.45
83.24
77.77
75.91
75.47
73.19
72.74
69.41
61.28
60.49
NH2
N
N
N
O
HO
HO
O
HO
HO
O
OH
OH
210 200 190 180 170 160 150 140 130 120 110 100 90 80 70 60 50 40 30 20 10
f1 (ppm)

^{13}C NMR

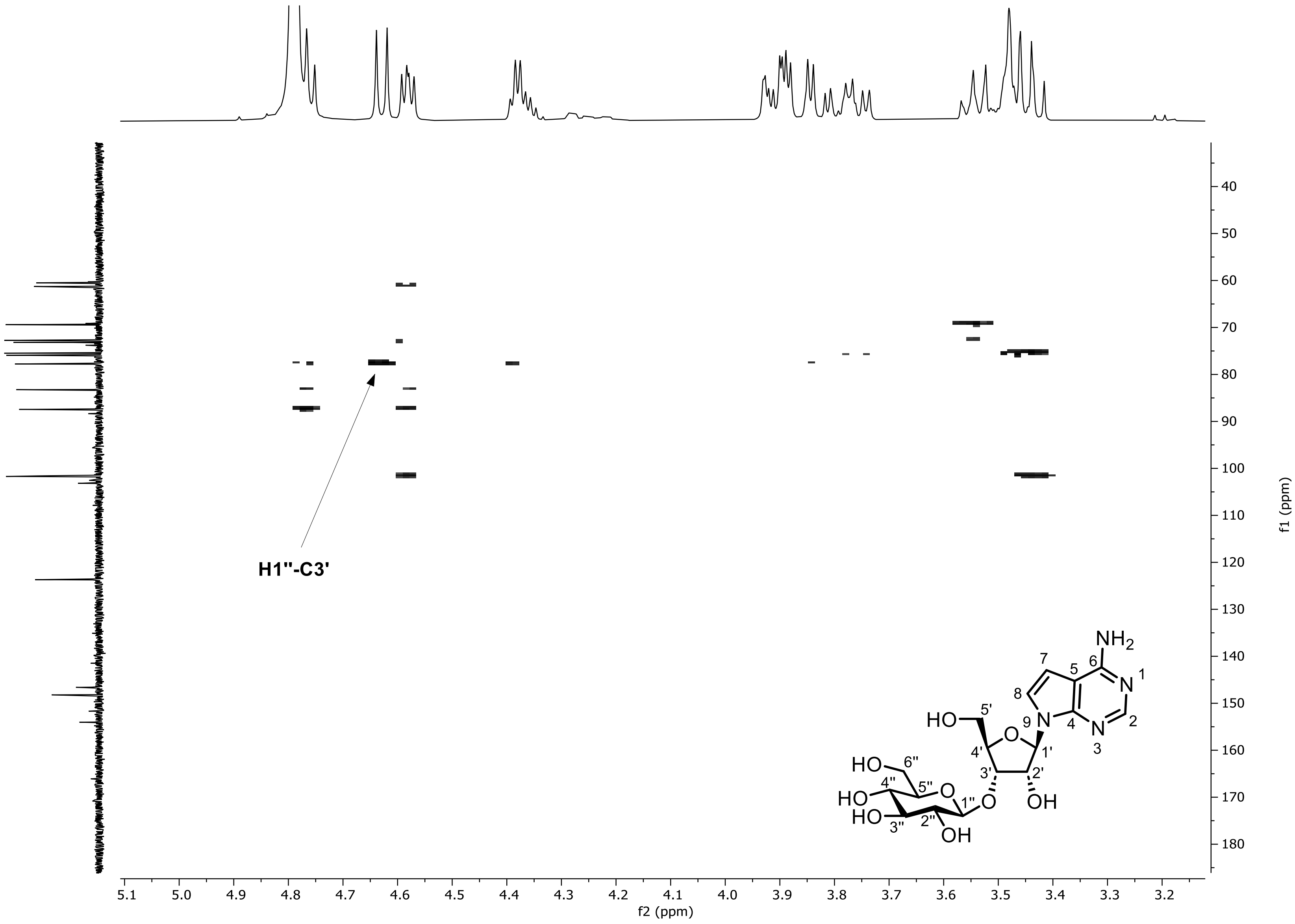

^{1}H-^{13}C HMBC

3’-O-β-Glucosylguanosine (3a)

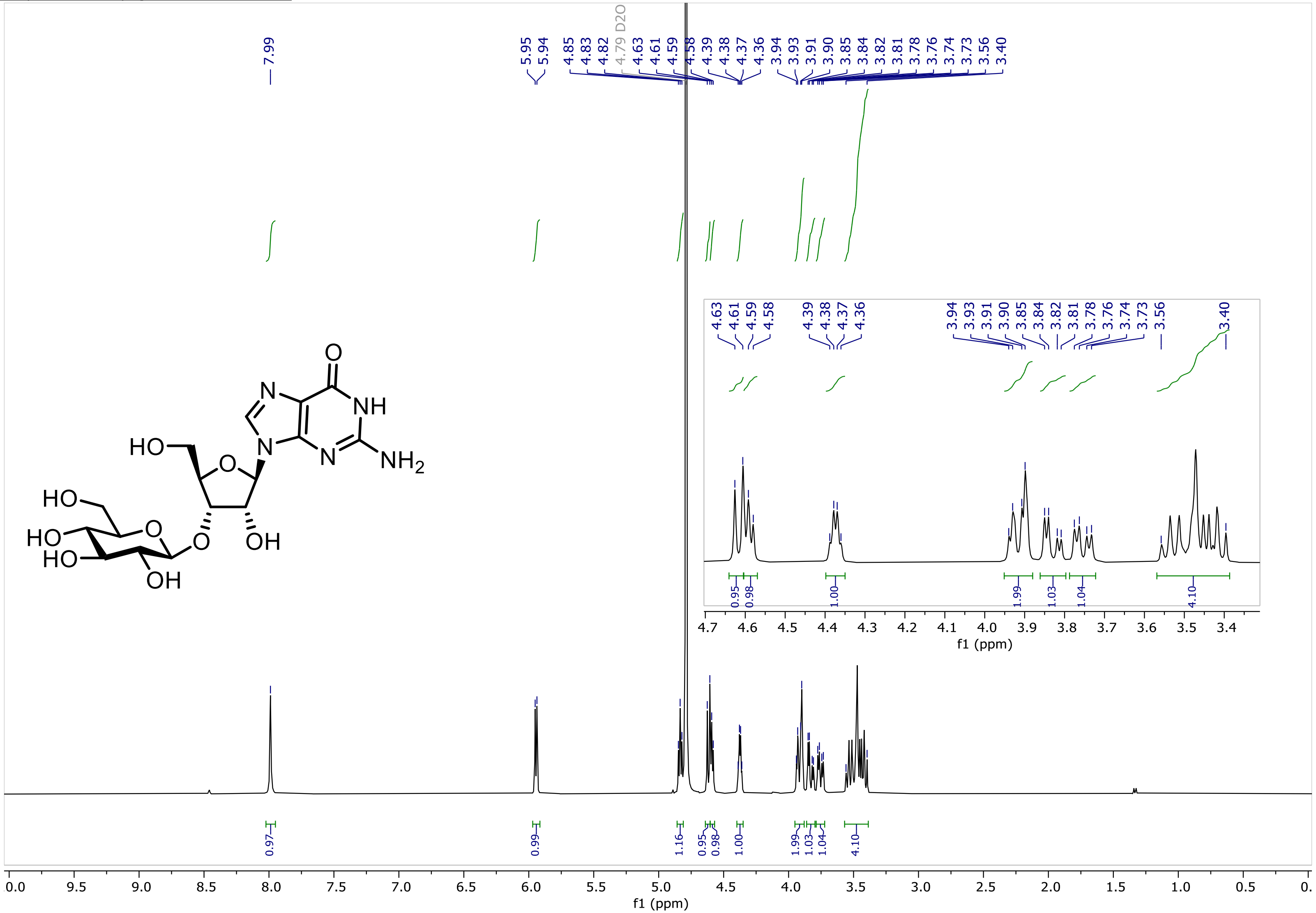

^{1}H NMR

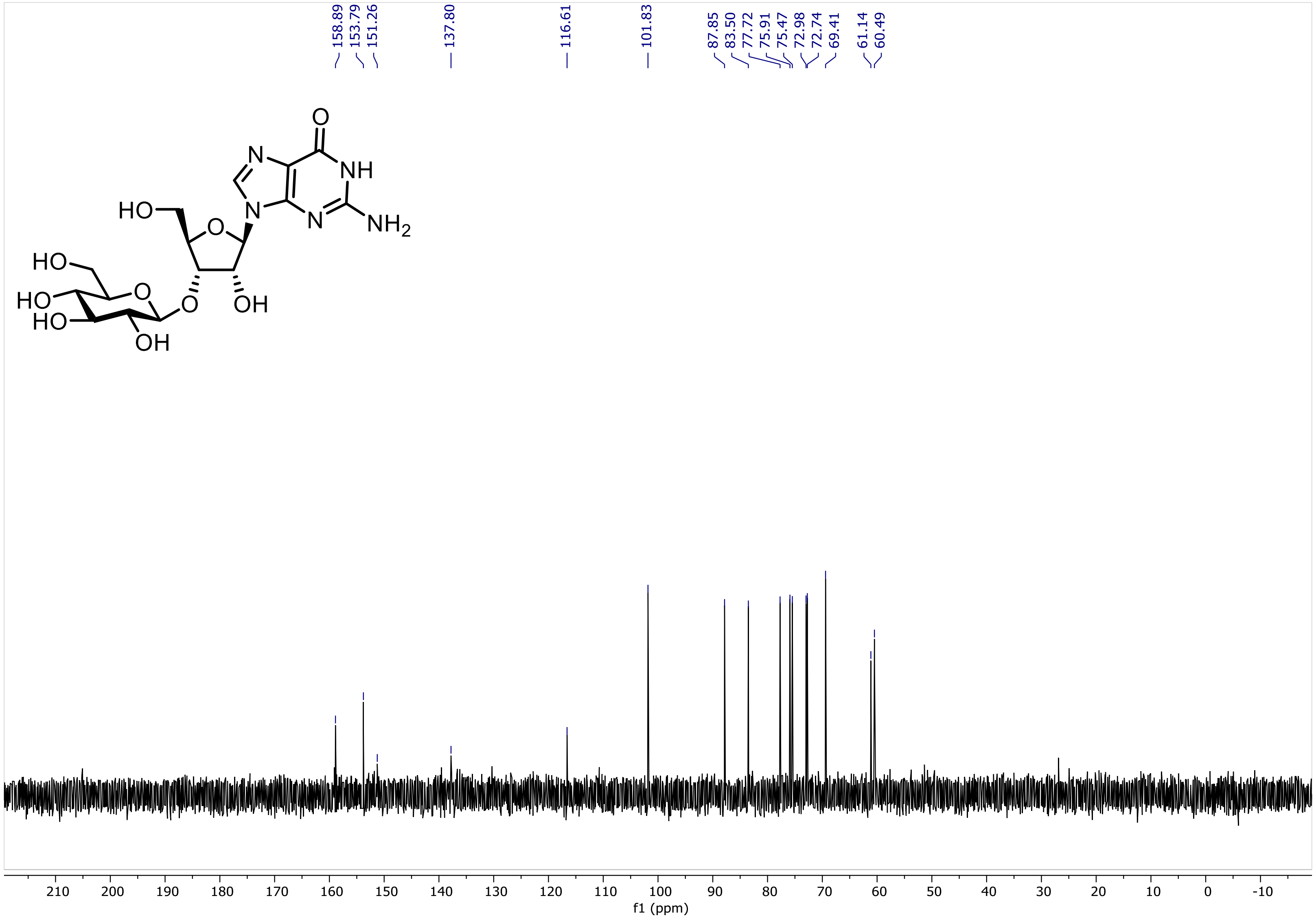

158.89
153.79
151.26
137.80
116.61
101.83
87.85
83.50
77.72
75.91
75.47
72.98
72.74
69.41
61.14
60.49
f1 (ppm)

^{13}C NMR

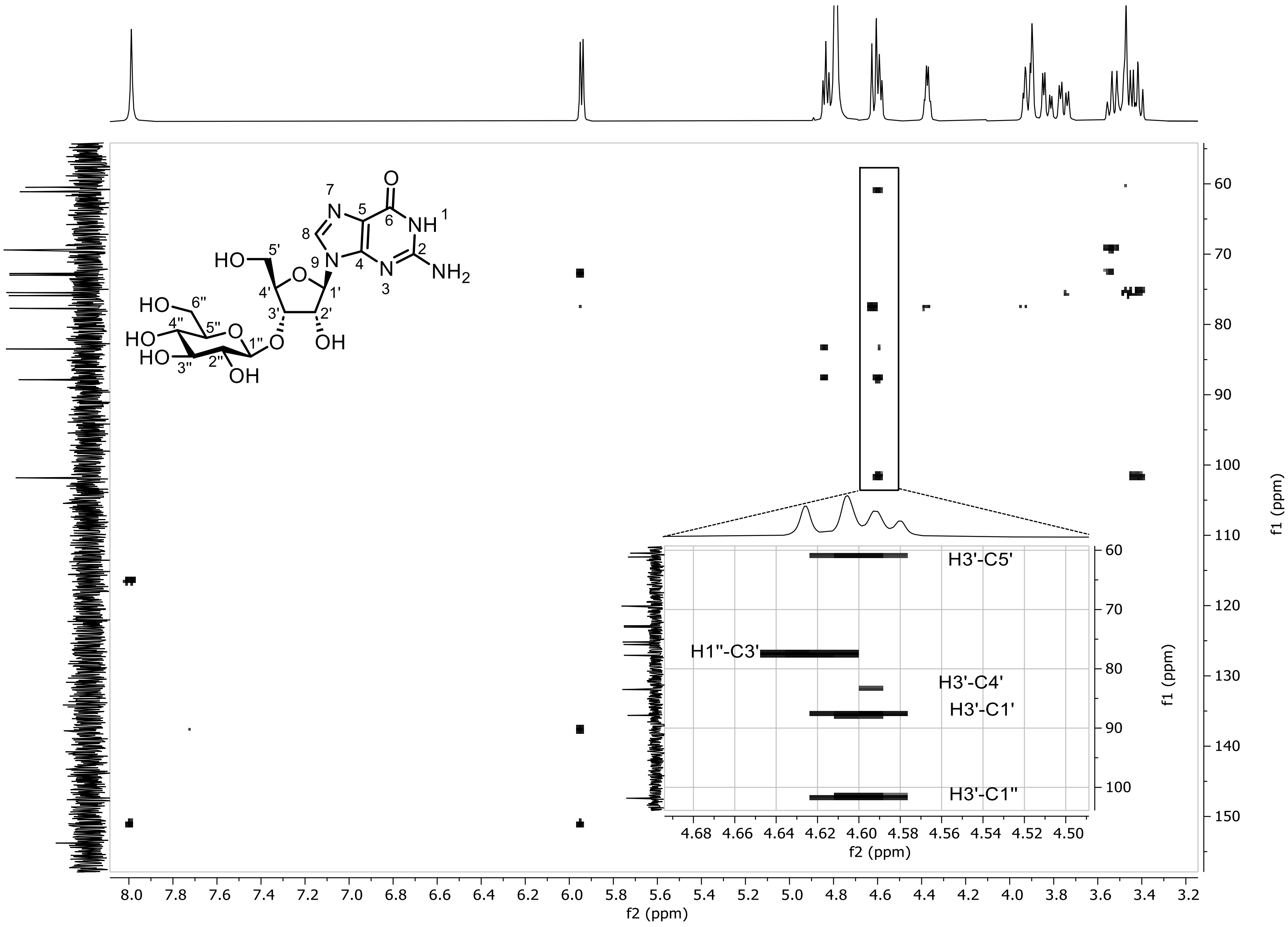

^{1}H-^{13}C HMBC

3’-O-β-Glucosylinosine (4a)

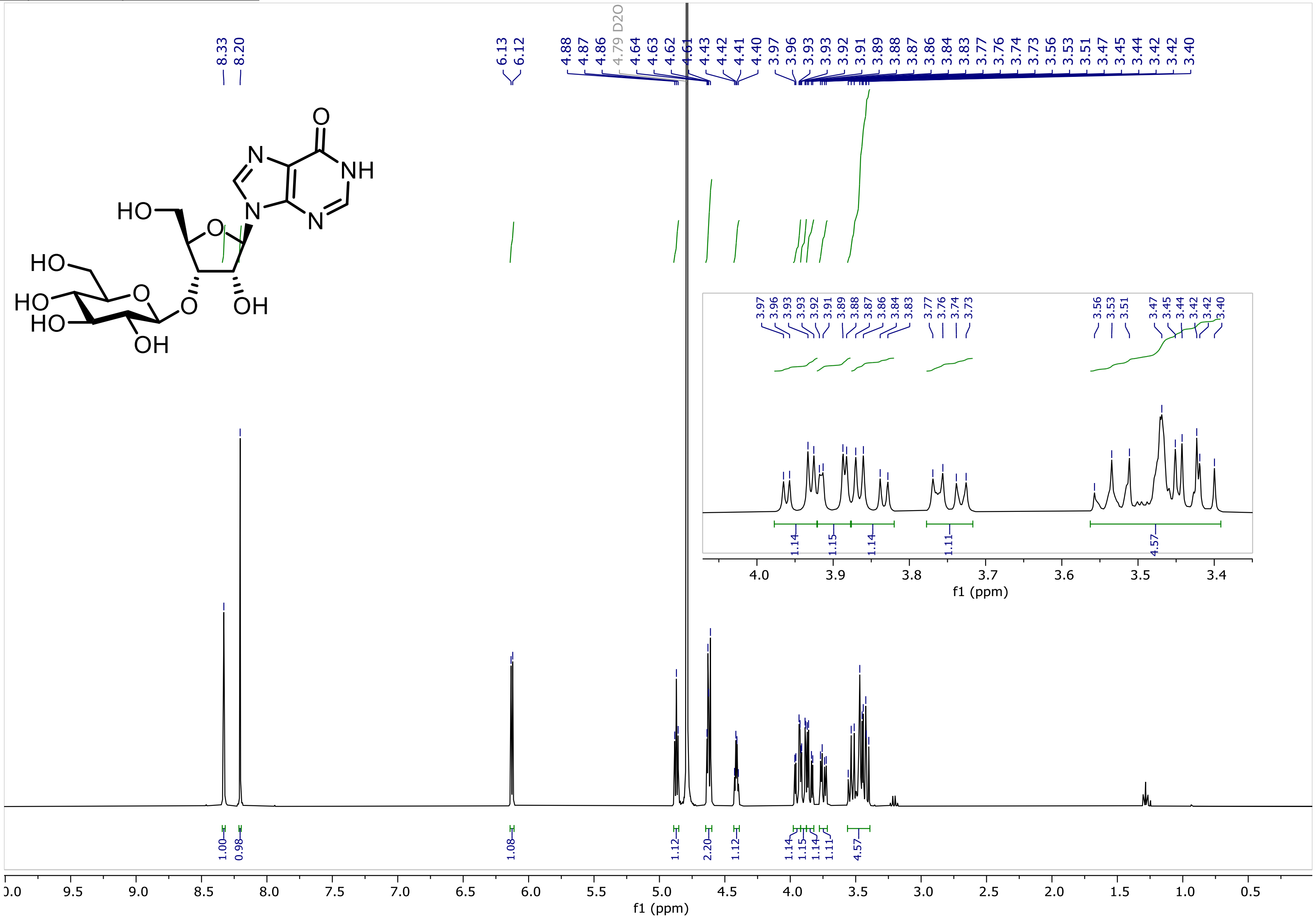

^{1}H NMR

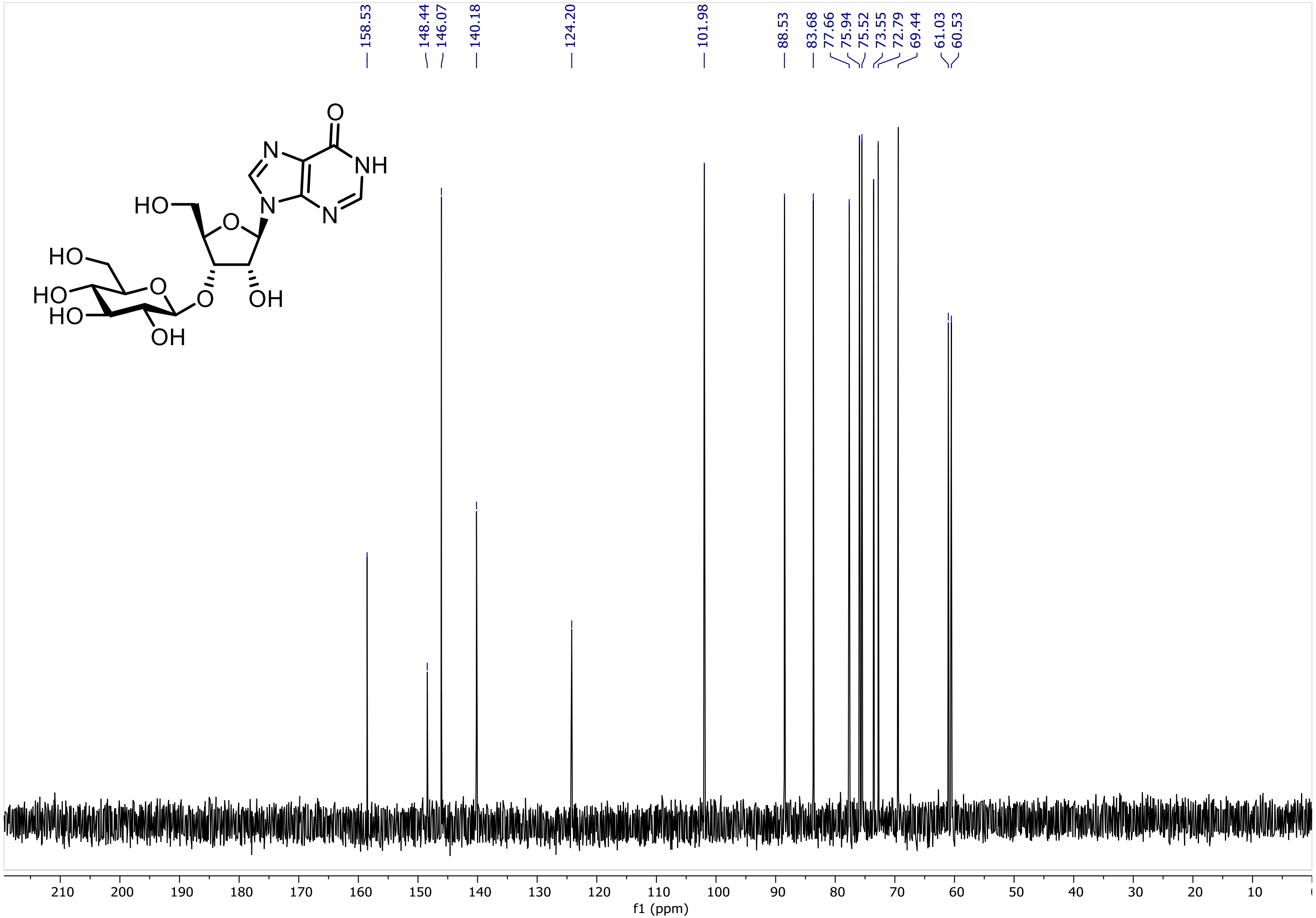
158.53
148.44
146.07
140.18
124.20
101.98
88.53
83.68
77.66
75.94
75.52
73.55
72.79
69.44
61.03
60.53
HO
HO
HO
HO
OH
OH
O
O
O
N
N
N
NH
O
210 200 190 180 170 160 150 140 130 120 110 100 90 80 70 60 50 40 30 20 10
f1 (ppm)

^{13}C NMR

9-(3′-O-Glucosyl-β-D-Arabinofuranosyl)adenine (5a)

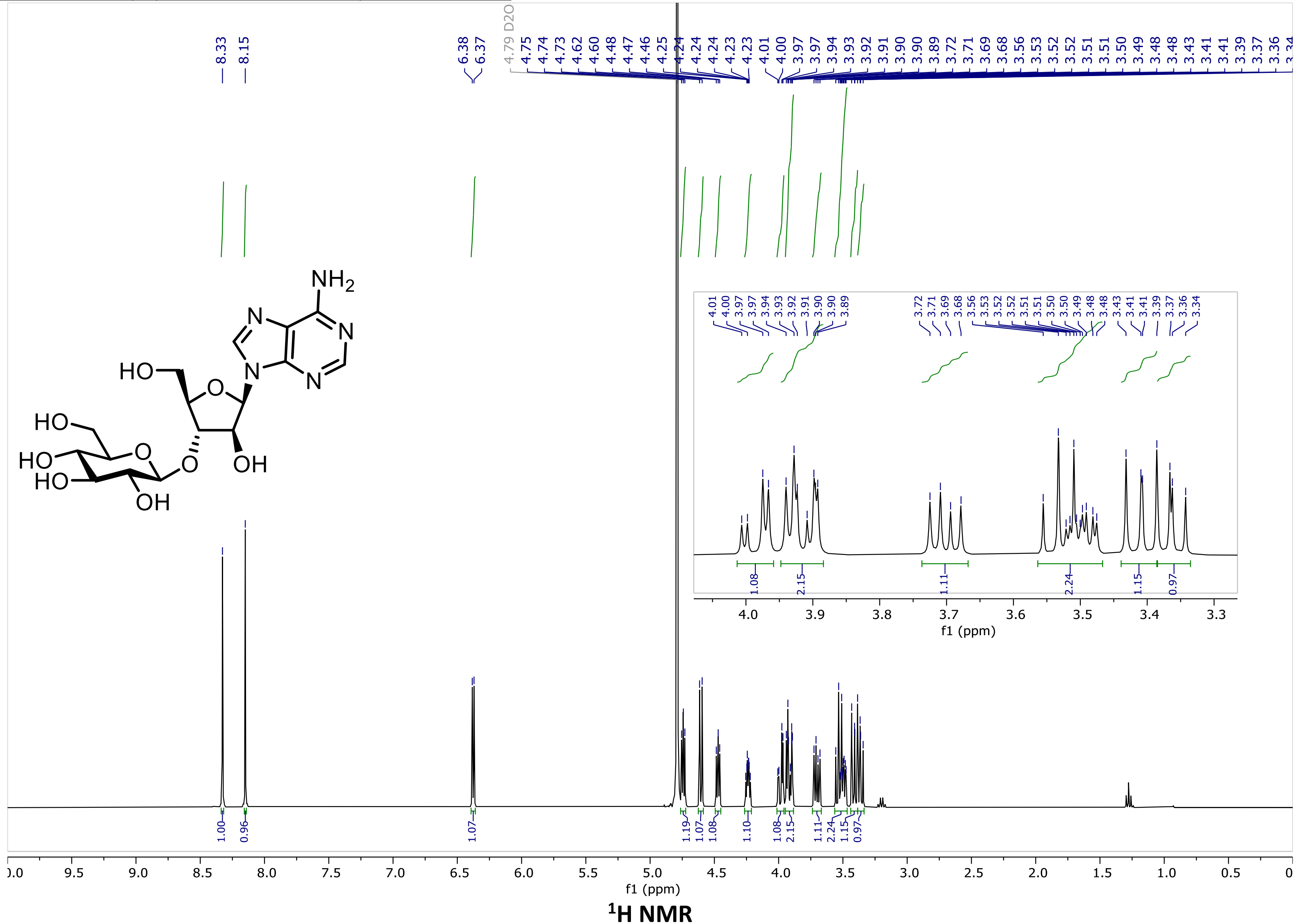

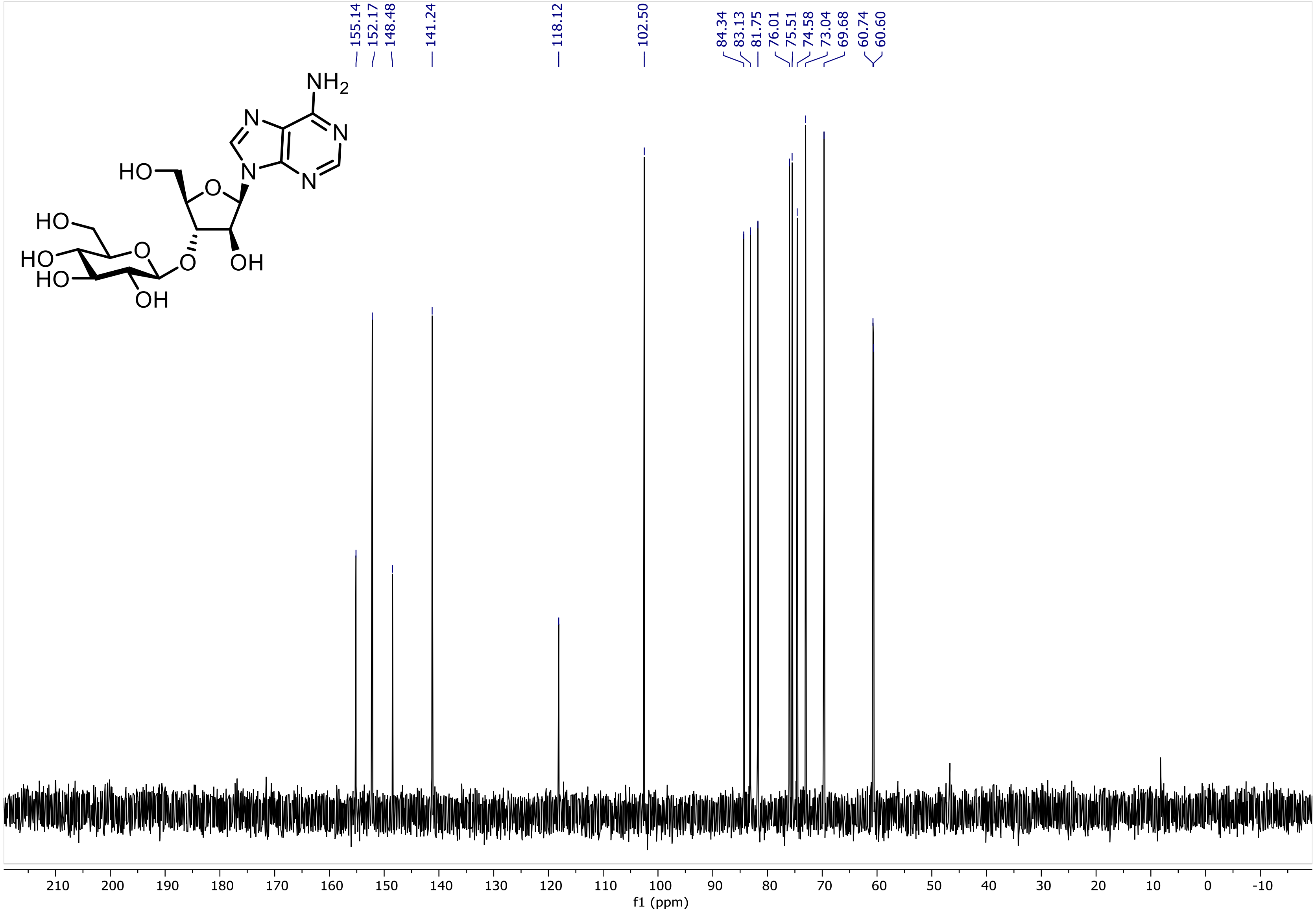
155.14
152.17
148.48
141.24
118.12
102.50
84.34
83.13
81.75
76.01
75.51
74.58
73.04
69.68
60.74
60.60
NH2
N
N
N
N
HO
O
HO
O
HO
HO
O
OH
OH
210
200
190
180
170
160
150
140
130
120
110
100
90
80
70
60
50
40
30
20
10
0
-10
f1 (ppm)

^{13}C NMR

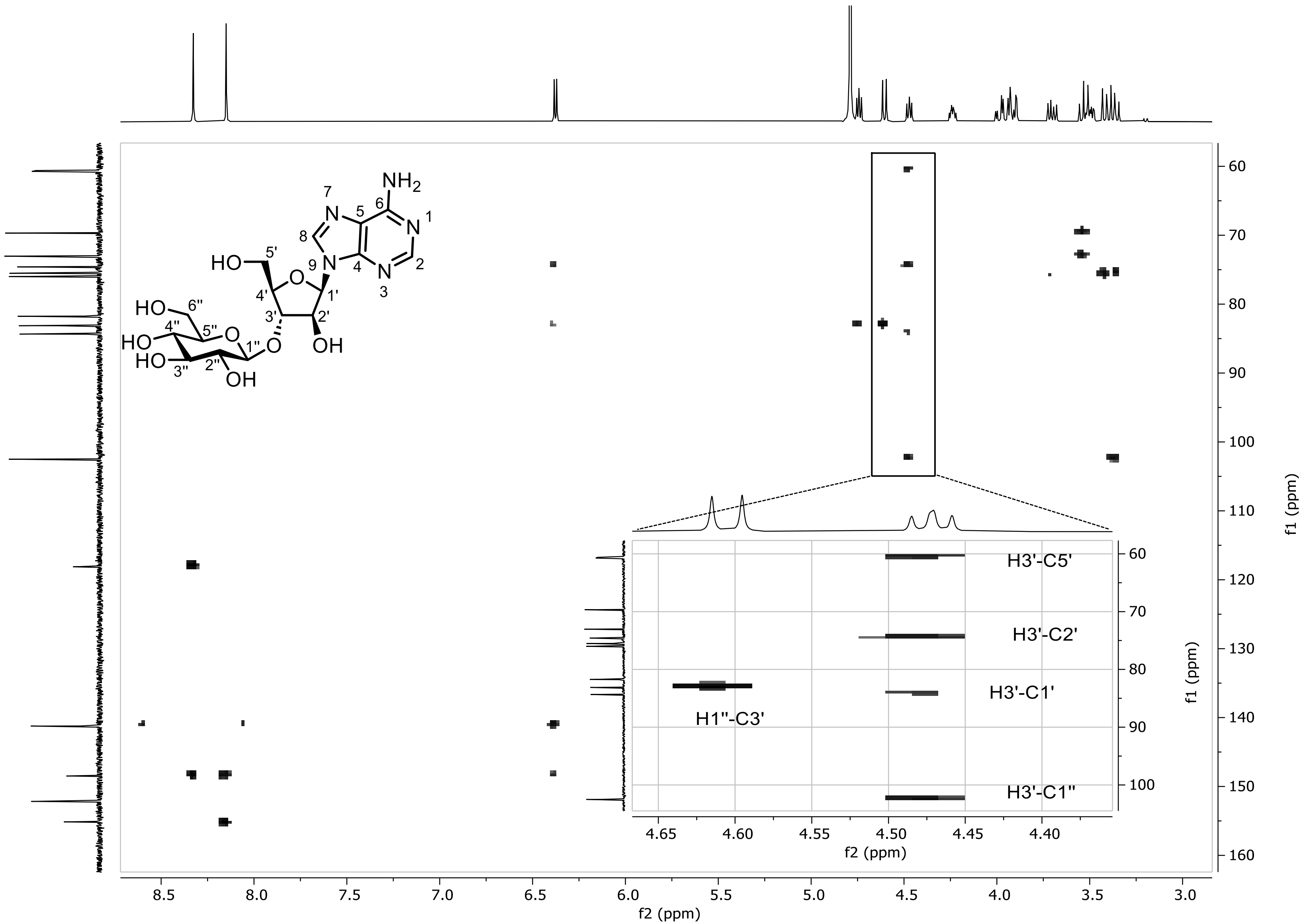

NH2
7
1
6
5
8
N
N
9
2
HO
5'
4
O
N
N
3
4'
1'
HO
6"
3'
2'
4"
5"
O
HO
1"
O
OH
HO
3"
2"
OH
H3'-C5'
H3'-C2'
H3'-C1'
H1"-C3'
H3'-C1"
f2 (ppm)
f1 (ppm)

^{1}H-^{13}C HMBC

3’-O-β-Glucosyluridine (11a)

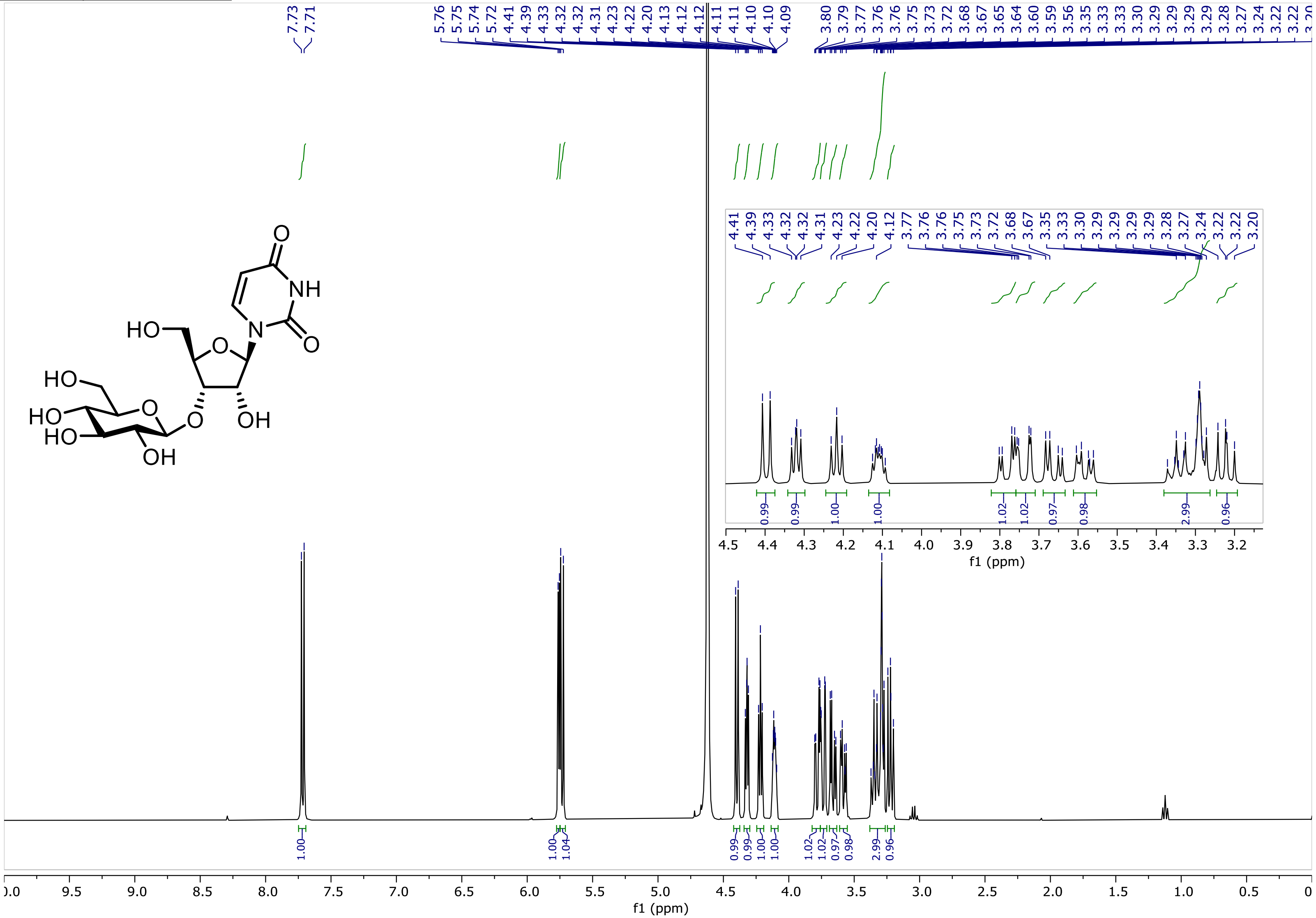

¹H NMR

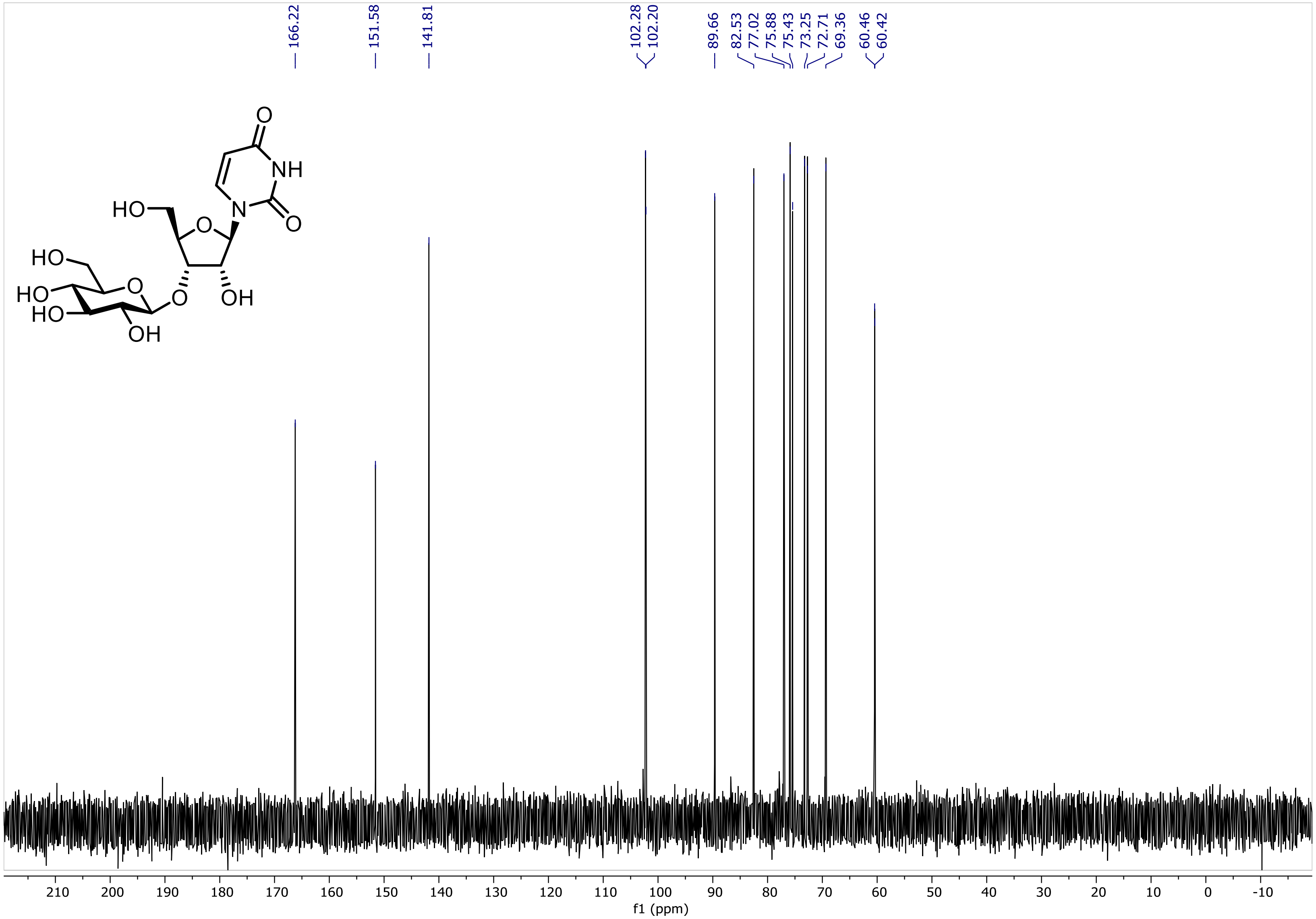
166.22
151.58
141.81
102.28
102.20
89.66
82.53
77.02
75.88
75.43
73.25
72.71
69.36
60.46
60.42
HO
HO
HO
HO
O
O
OH
O
N
NH
O
O
OH
210 200 190 180 170 160 150 140 130 120 110 100 90 80 70 60 50 40 30 20 10 0 -10
f1 (ppm)

^{13}C NMR

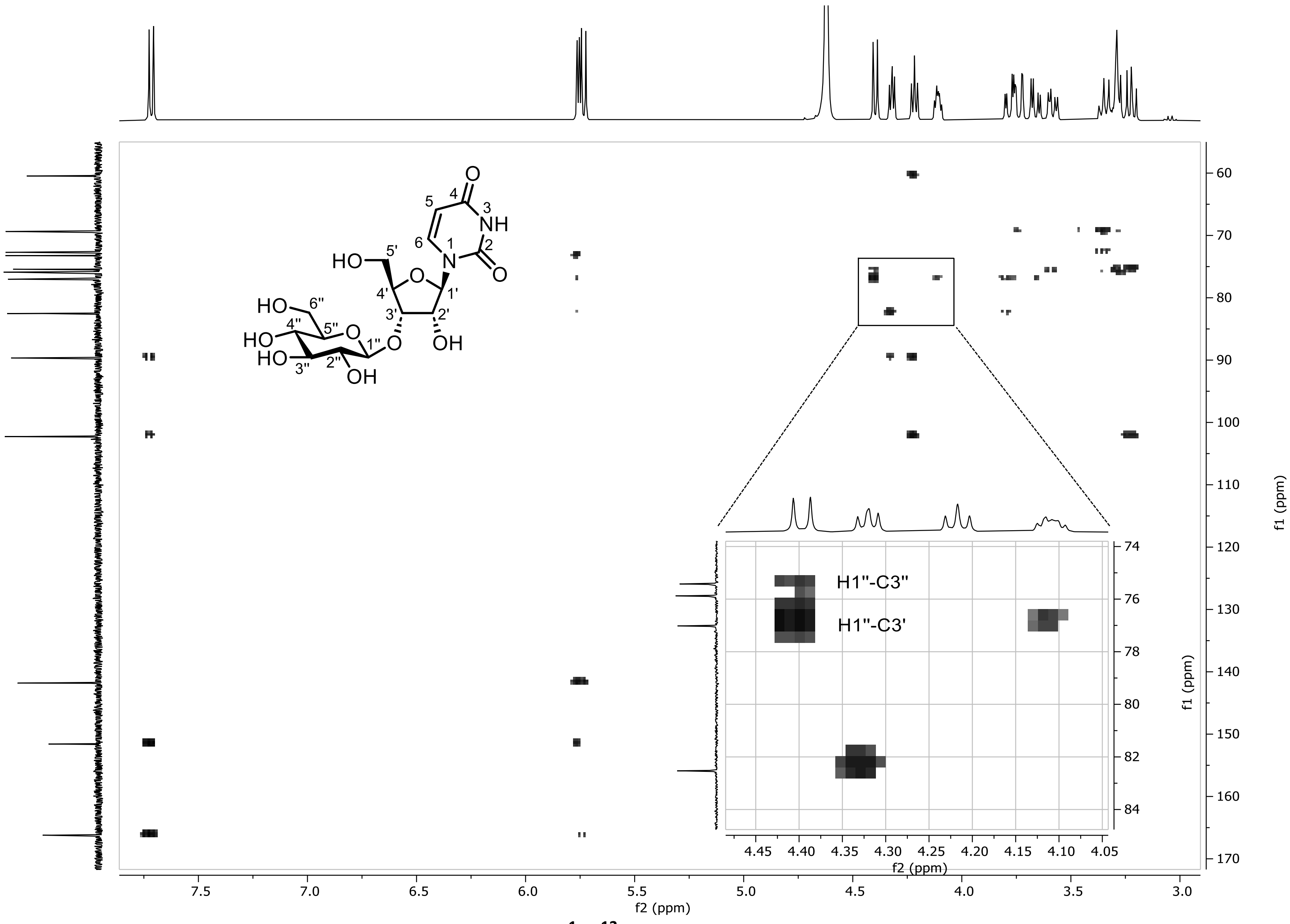

^{1}H-^{13}C HMBC

3’-O-β-Glucosylcytidine (12a)

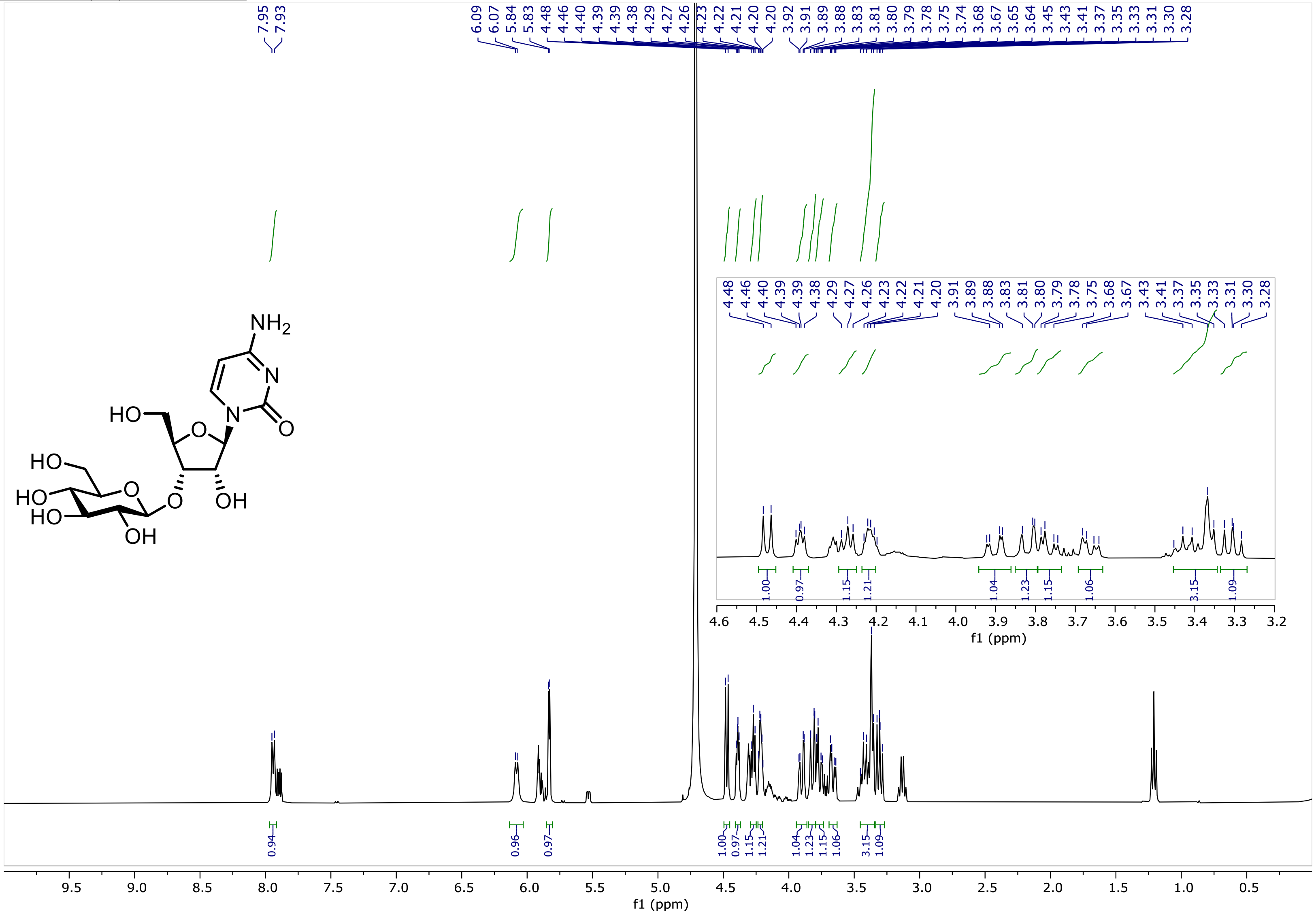

^{1}H NMR

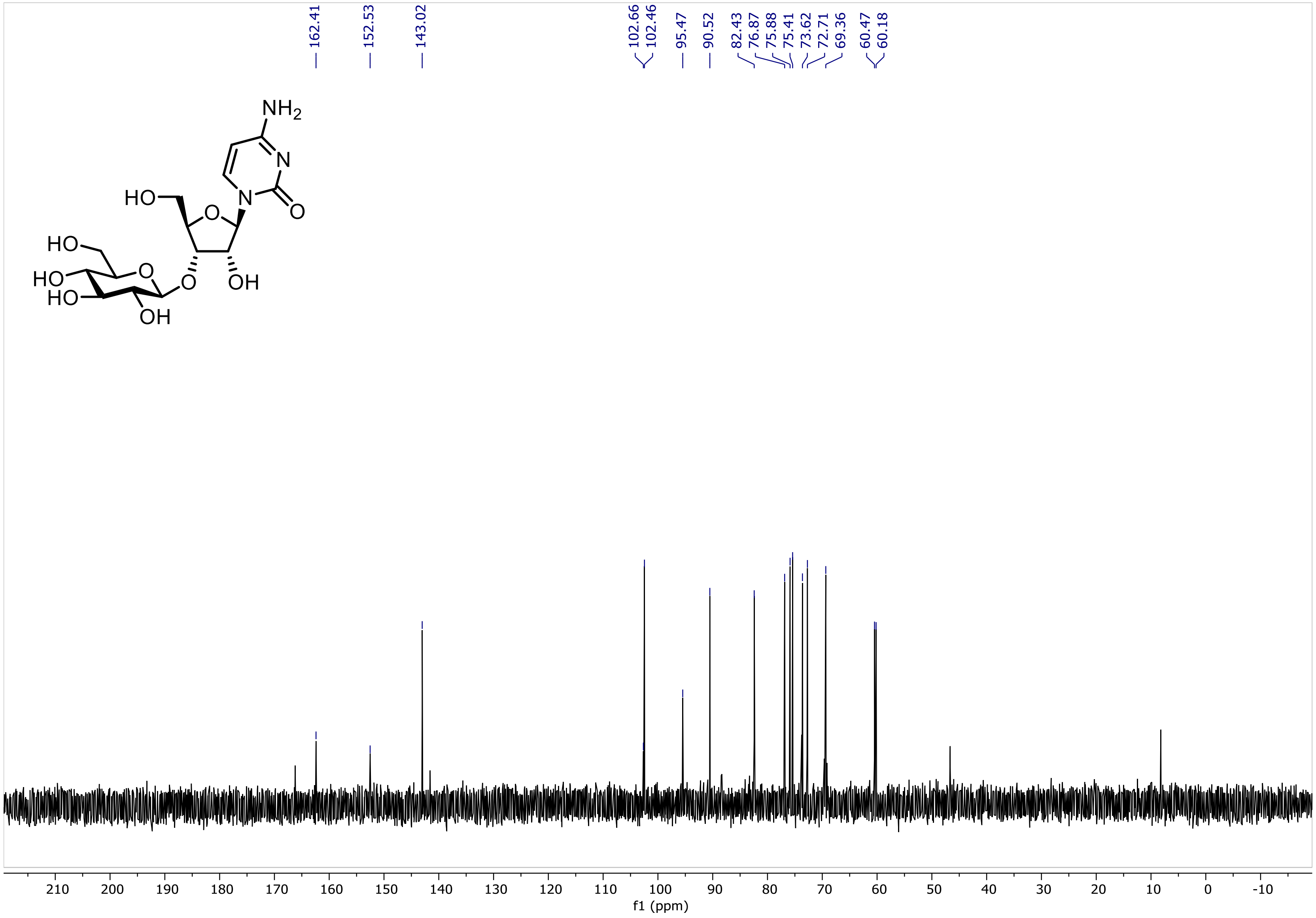

162.41
152.53
143.02
102.66
102.46
95.47
90.52
82.43
76.87
75.88
75.41
73.62
72.71
69.36
60.47
60.18
NH2
N
O
HO
OH
210 200 190 180 170 160 150 140 130 120 110 100 90 80 70 60 50 40 30 20 10 0 -10
f1 (ppm)

^{13}C NMR

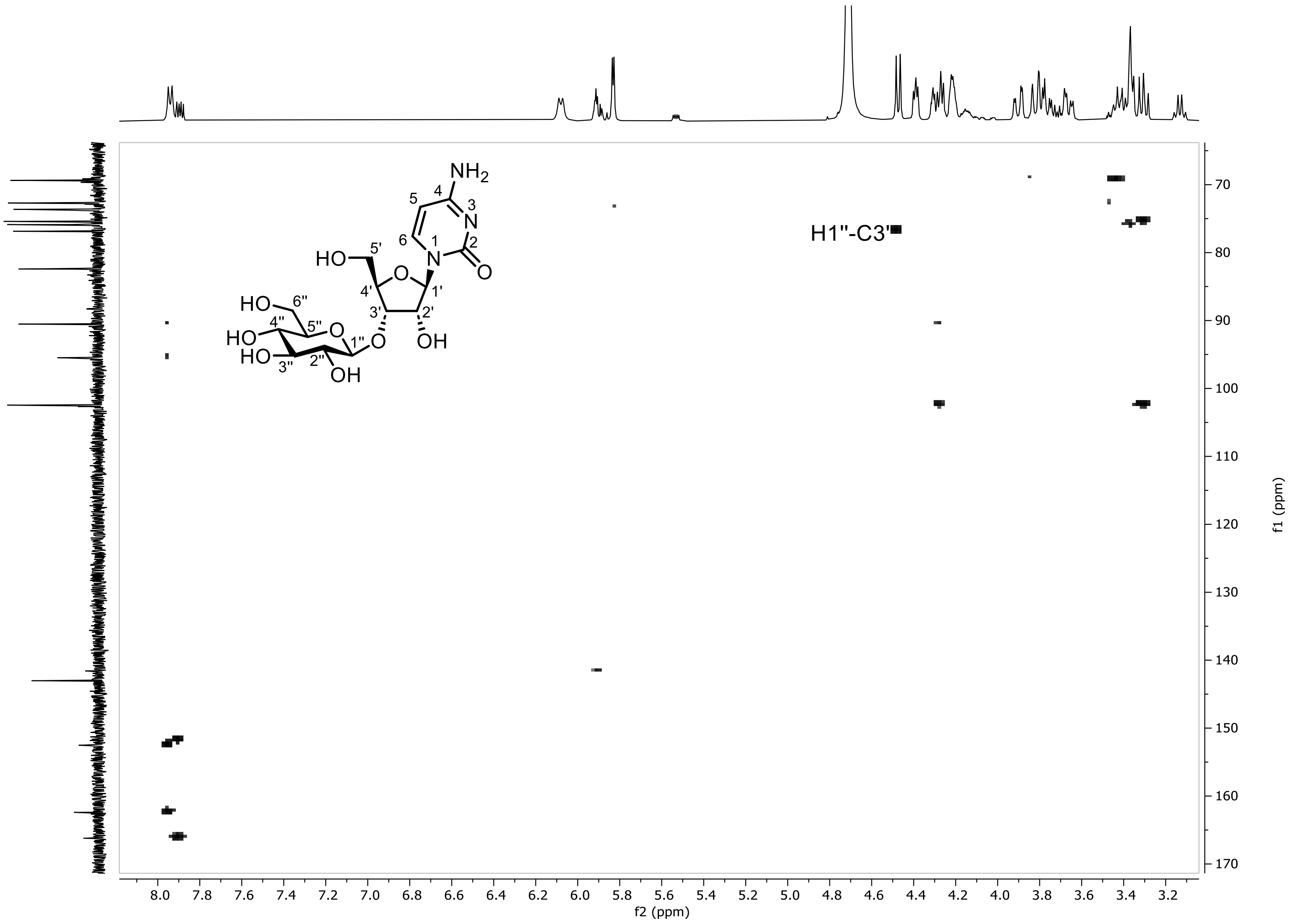
NH2
5
4
3
N
6
1
2
HO
5'
O
N
O
1'
4'
HO
6''
3'
2'
4''
5''
O
HO
O
1''
OH
HO
2''
3''
OH
H1''-C3'
f1 (ppm)
f2 (ppm)

^{1}H-^{13}C HMBC

N^6-Propargyladenosine (15)

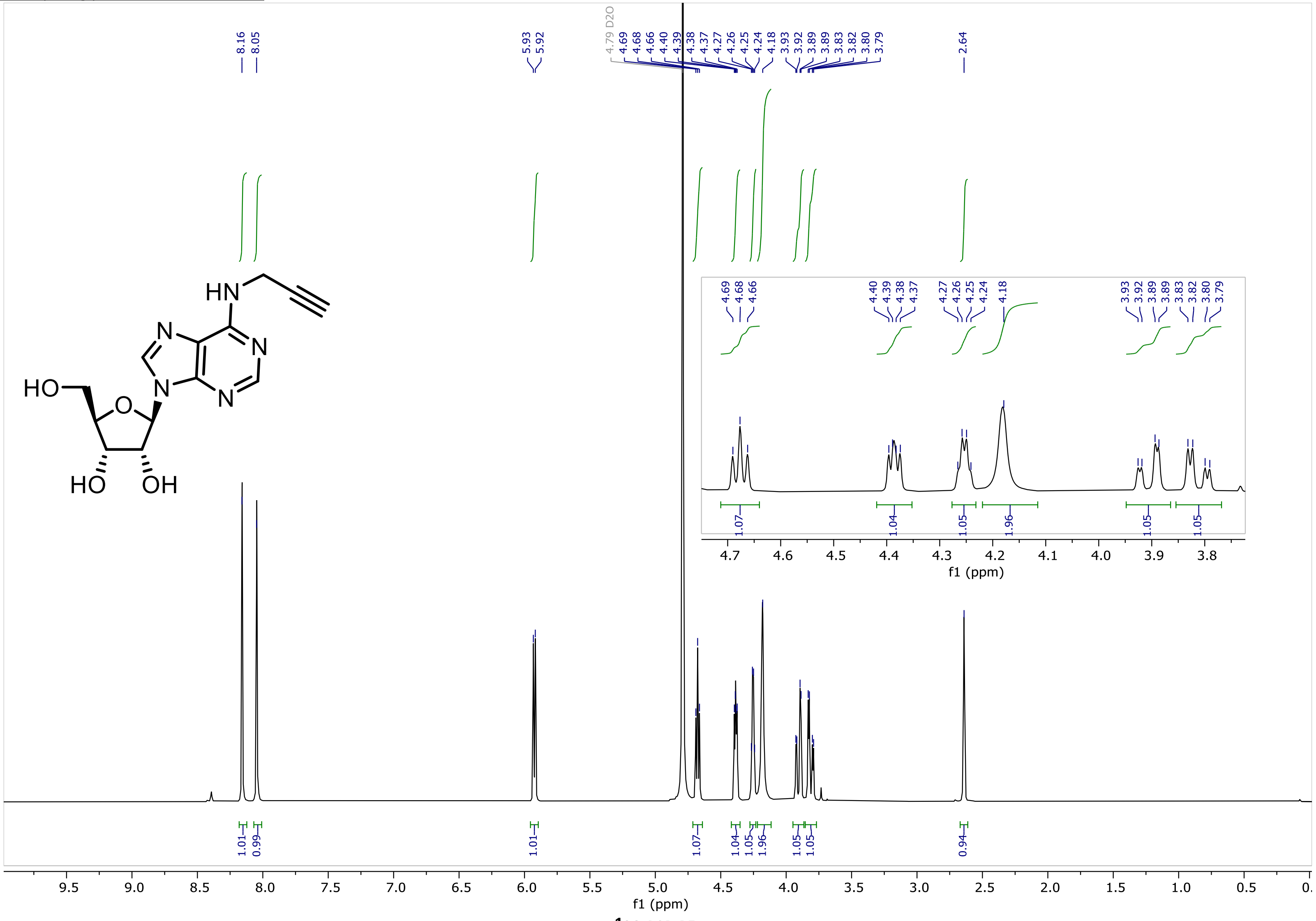

^{1}H NMR

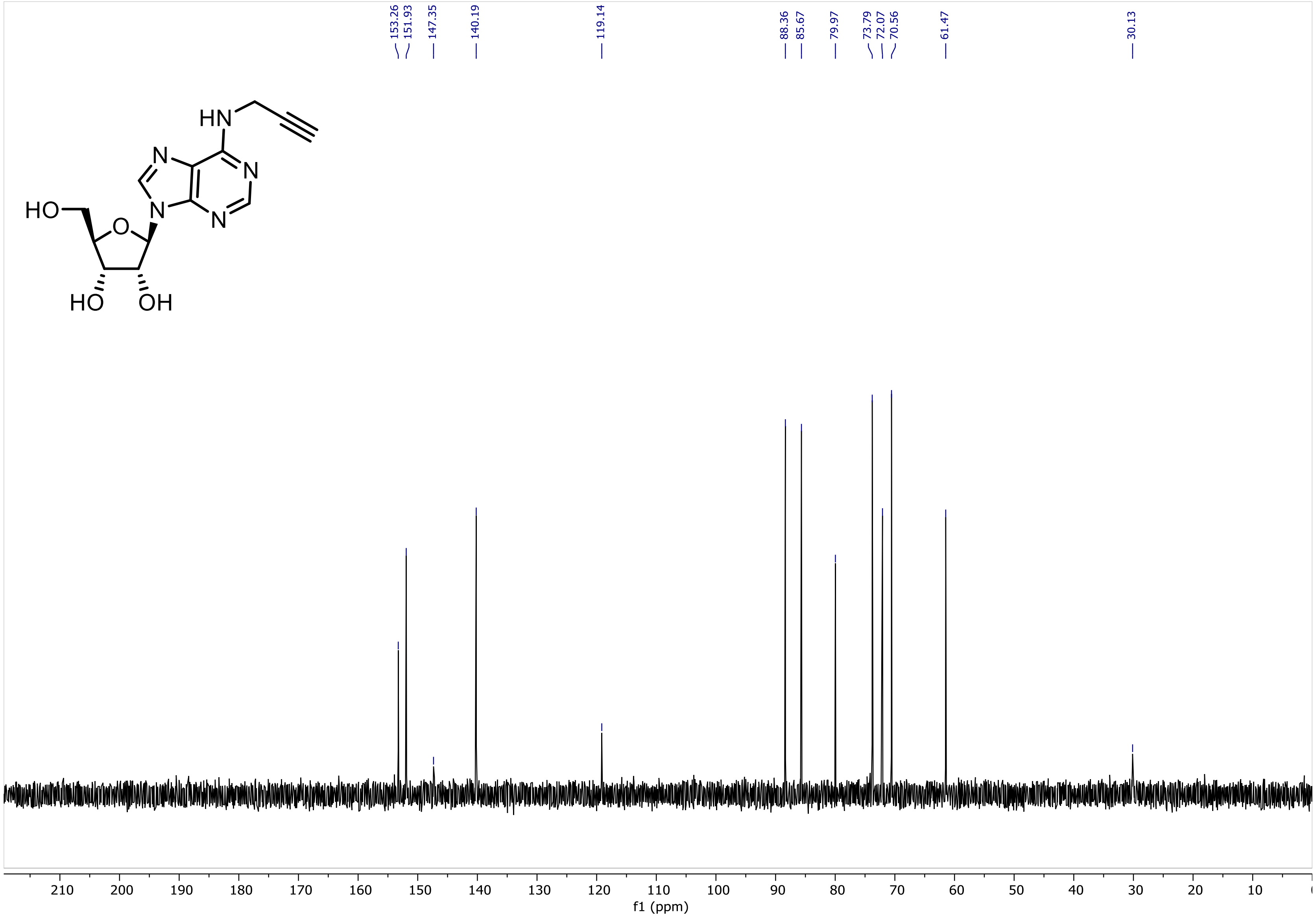
153.26
151.93
147.35
140.19
119.14
88.36
85.67
79.97
73.79
72.07
70.56
61.47
30.13
HN
N
N
N
N
HO
O
HO
OH
f1 (ppm)

^{13}C NMR

3’-*O*-β-Glucosyl-*N*^{6}-propargyladenosine (15a)

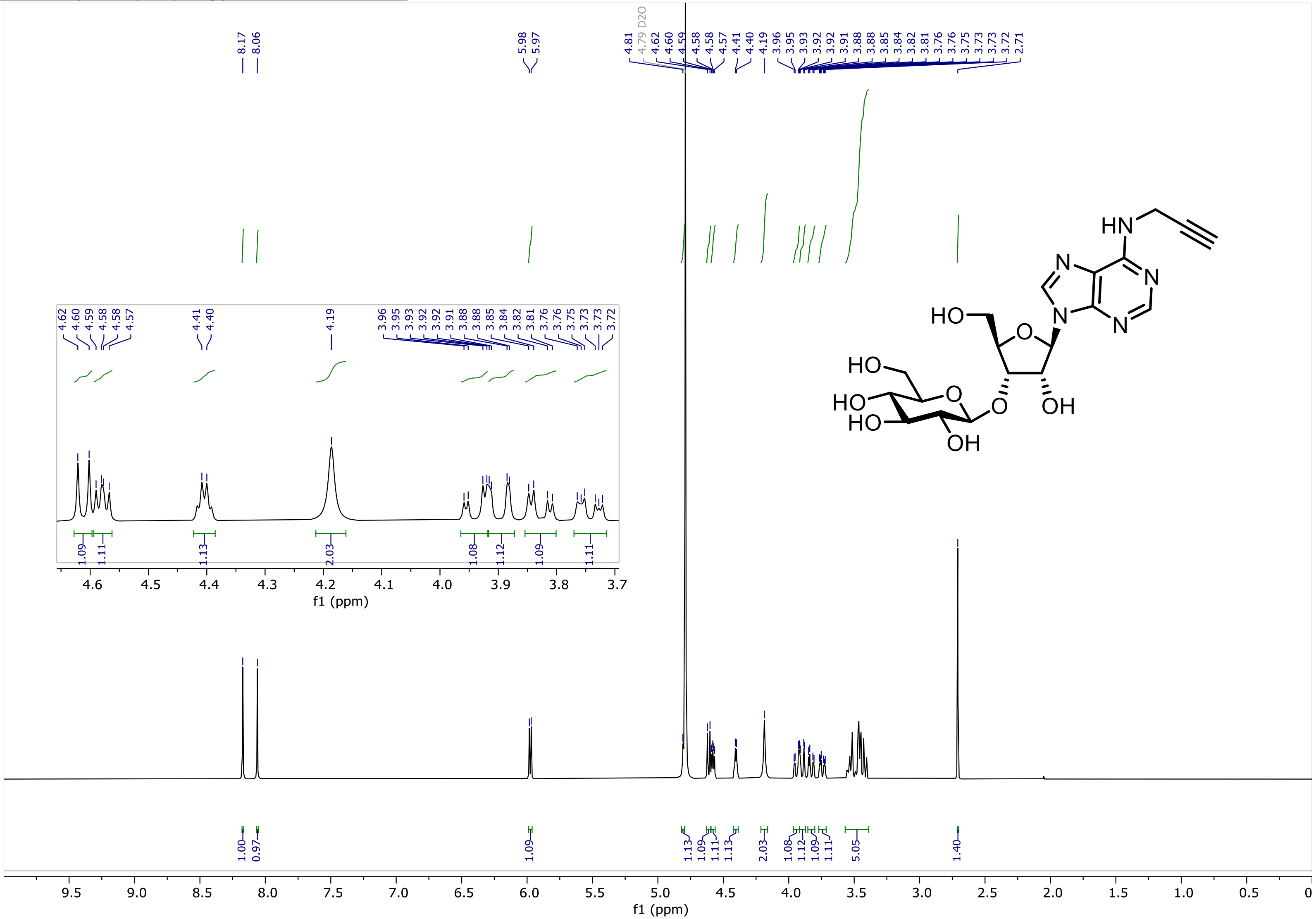

^{1}H NMR

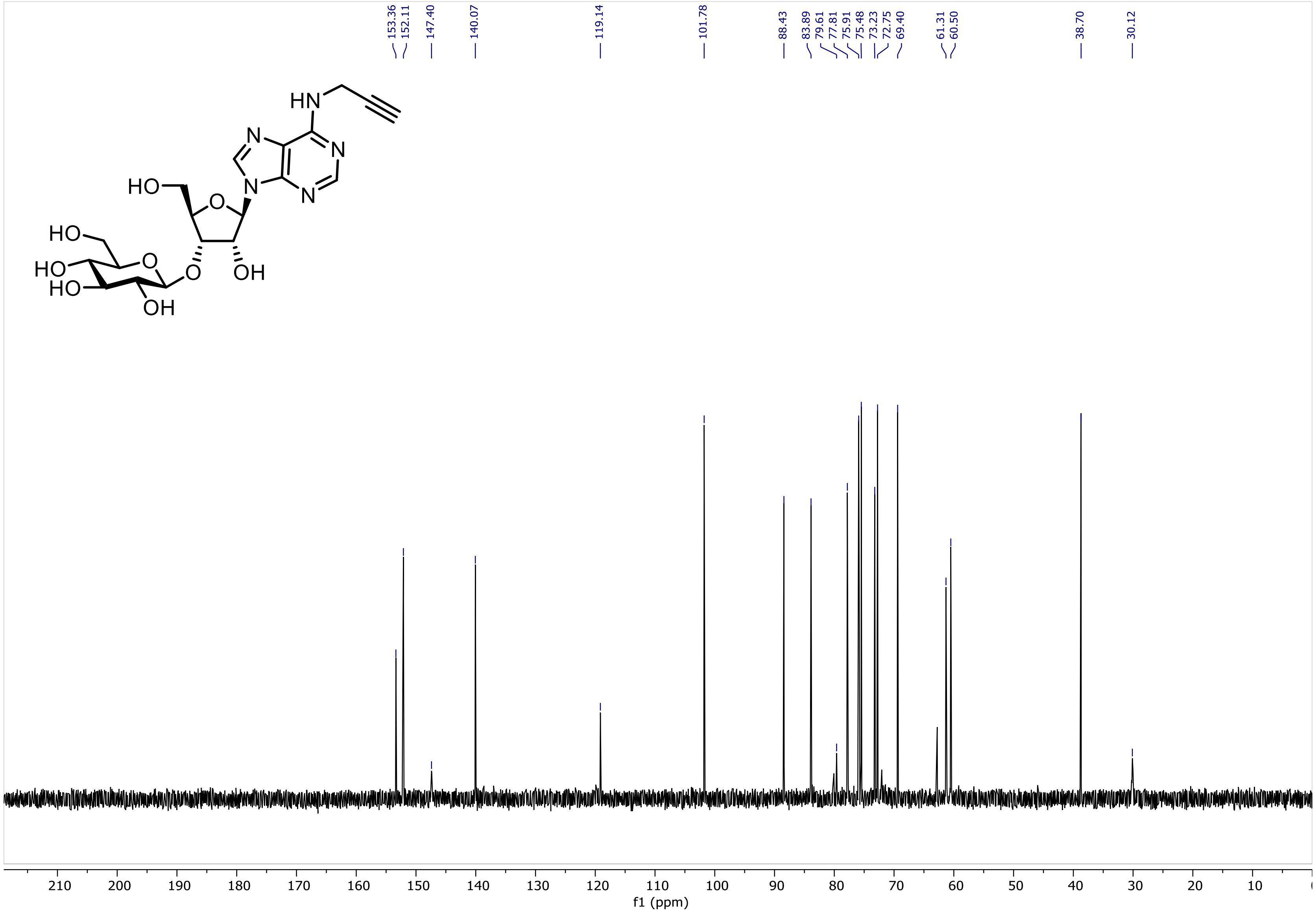
153.36
152.11
147.40
140.07
119.14
101.78
88.43
83.89
79.61
77.81
75.91
75.48
73.23
72.75
69.40
61.31
60.50
38.70
30.12
HN
N
N
N
N
HO
O
HO
HO
HO
O
O
OH
OH
210 200 190 180 170 160 150 140 130 120 110 100 90 80 70 60 50 40 30 20 10
f1 (ppm)

^{13}C NMR

3’-O-β-Glucosyl-6-O-methylguanosine (16a)

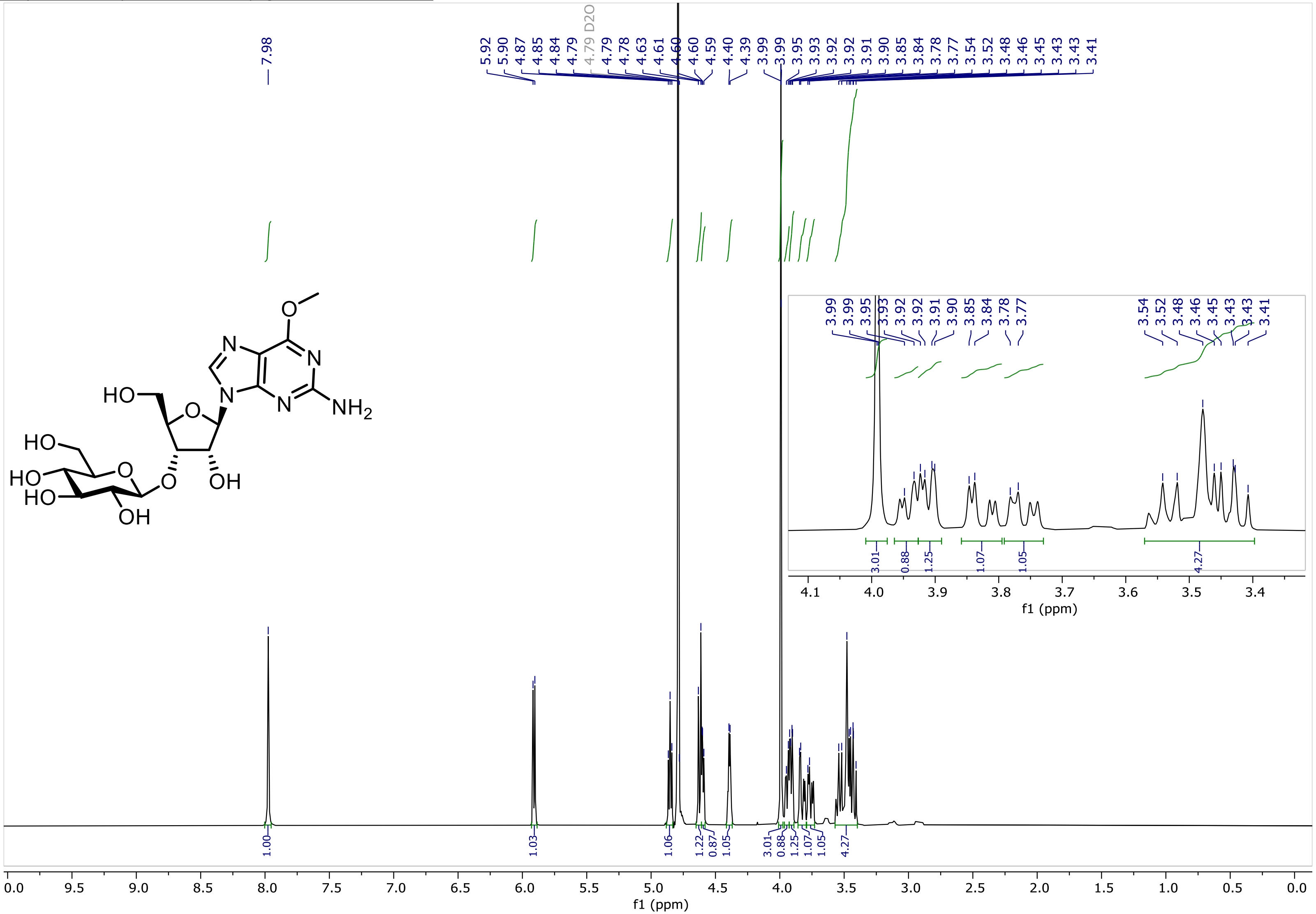

^{1}H NMR

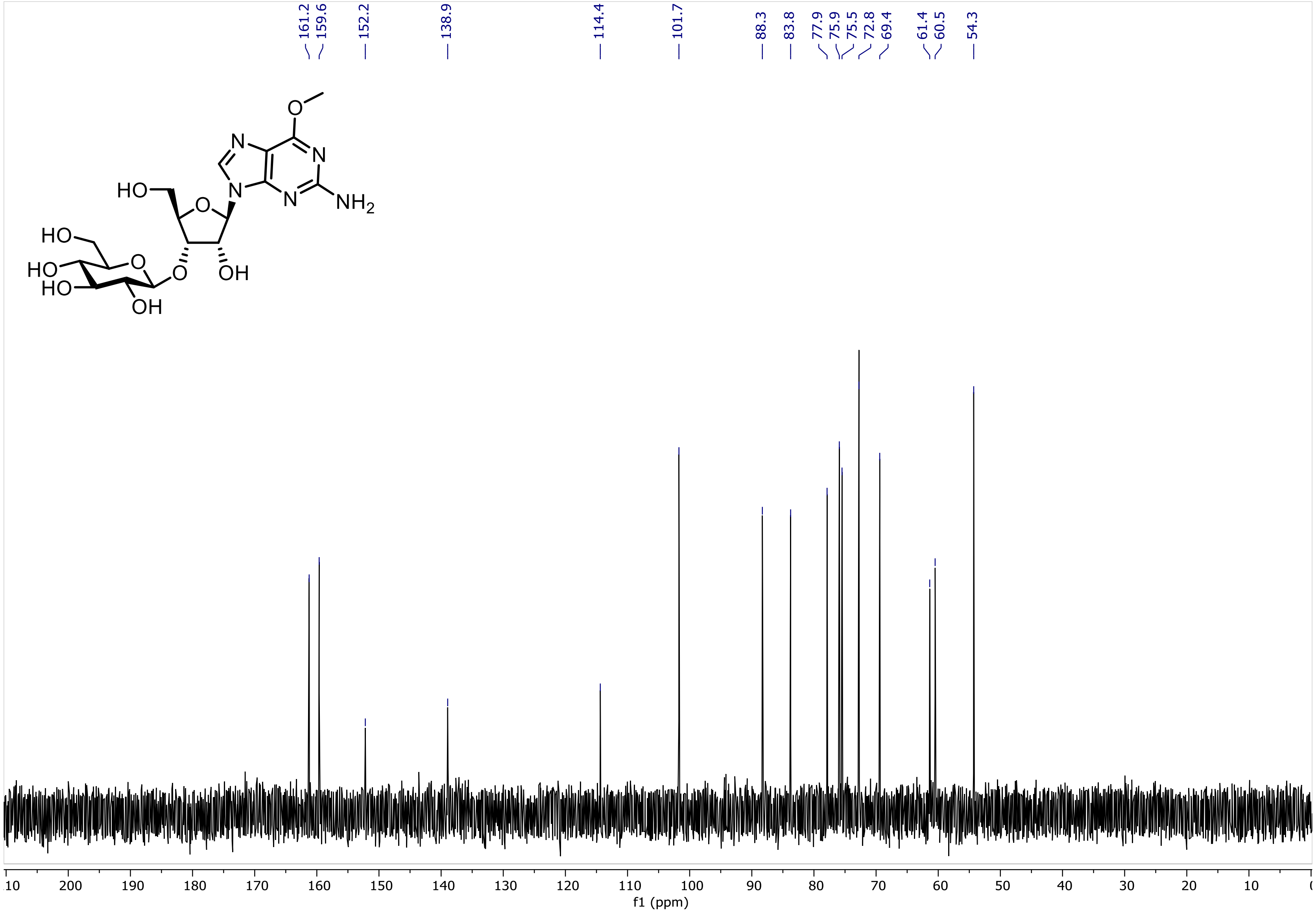
161.2
159.6
152.2
138.9
114.4
101.7
88.3
83.8
77.9
75.9
75.5
72.8
69.4
61.4
60.5
54.3
f1 (ppm)

^{13}C NMR

3’-O-β-Glucosylnelarabine (17a)

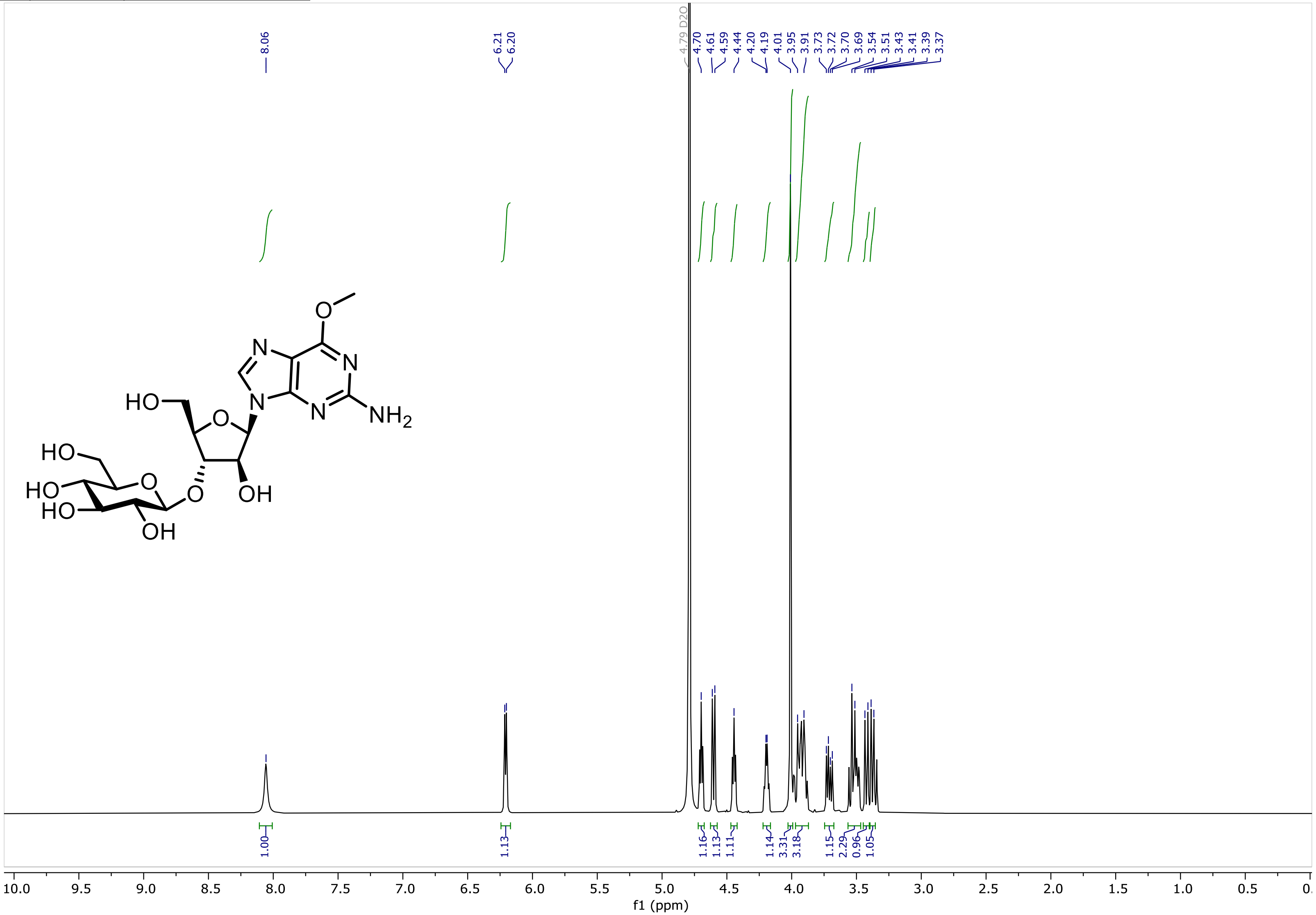

^{1}H NMR

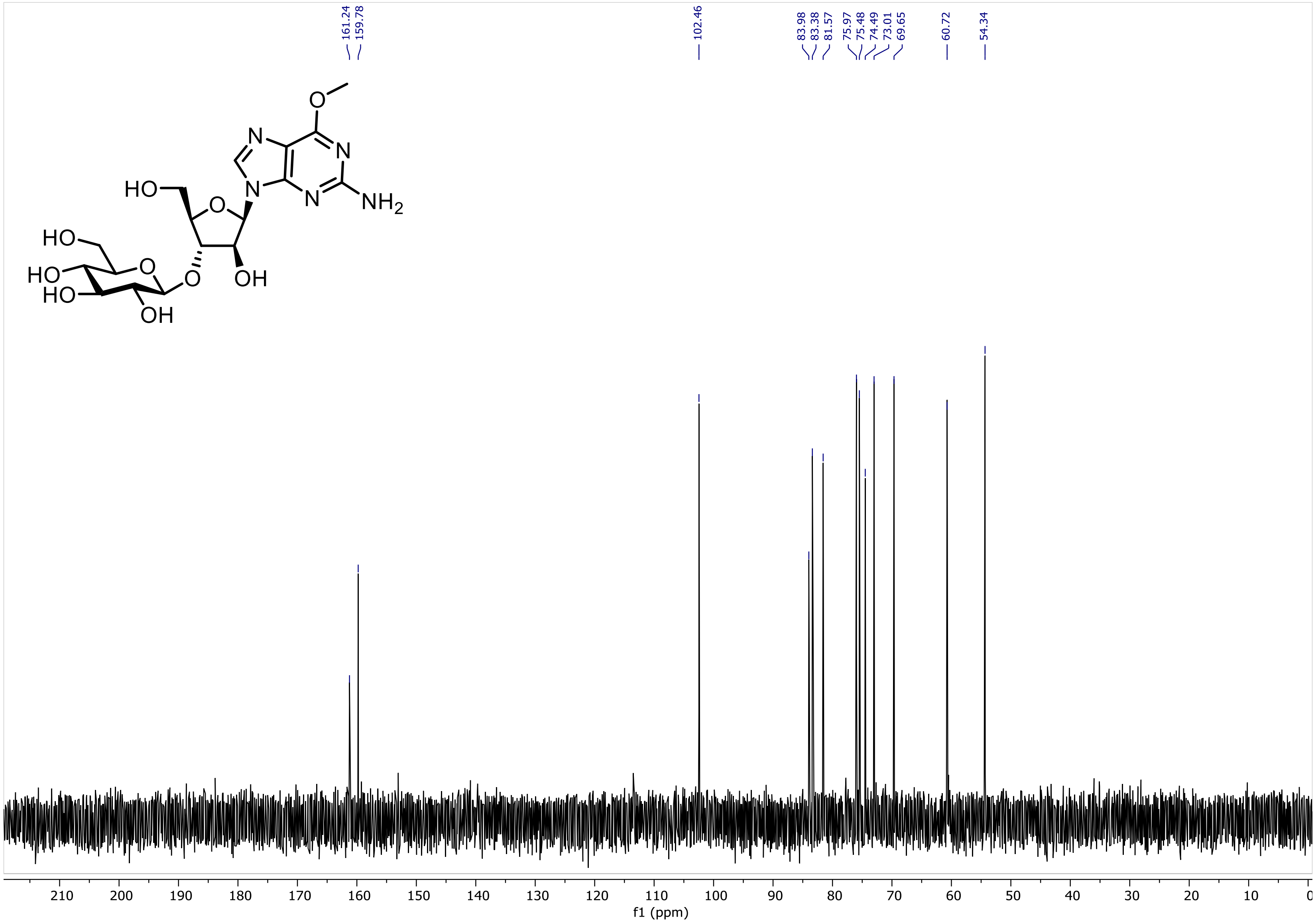
161.24
159.78
102.46
83.98
83.38
81.57
75.97
75.48
74.49
73.01
69.65
60.72
54.34
O
N
N
N
N
NH2
HO
O
HO
HO
HO
O
O
OH
OH
210
200
190
180
170
160
150
140
130
120
110
100
90
80
70
60
50
40
30
20
10
0
f1 (ppm)

^{13}C NMR

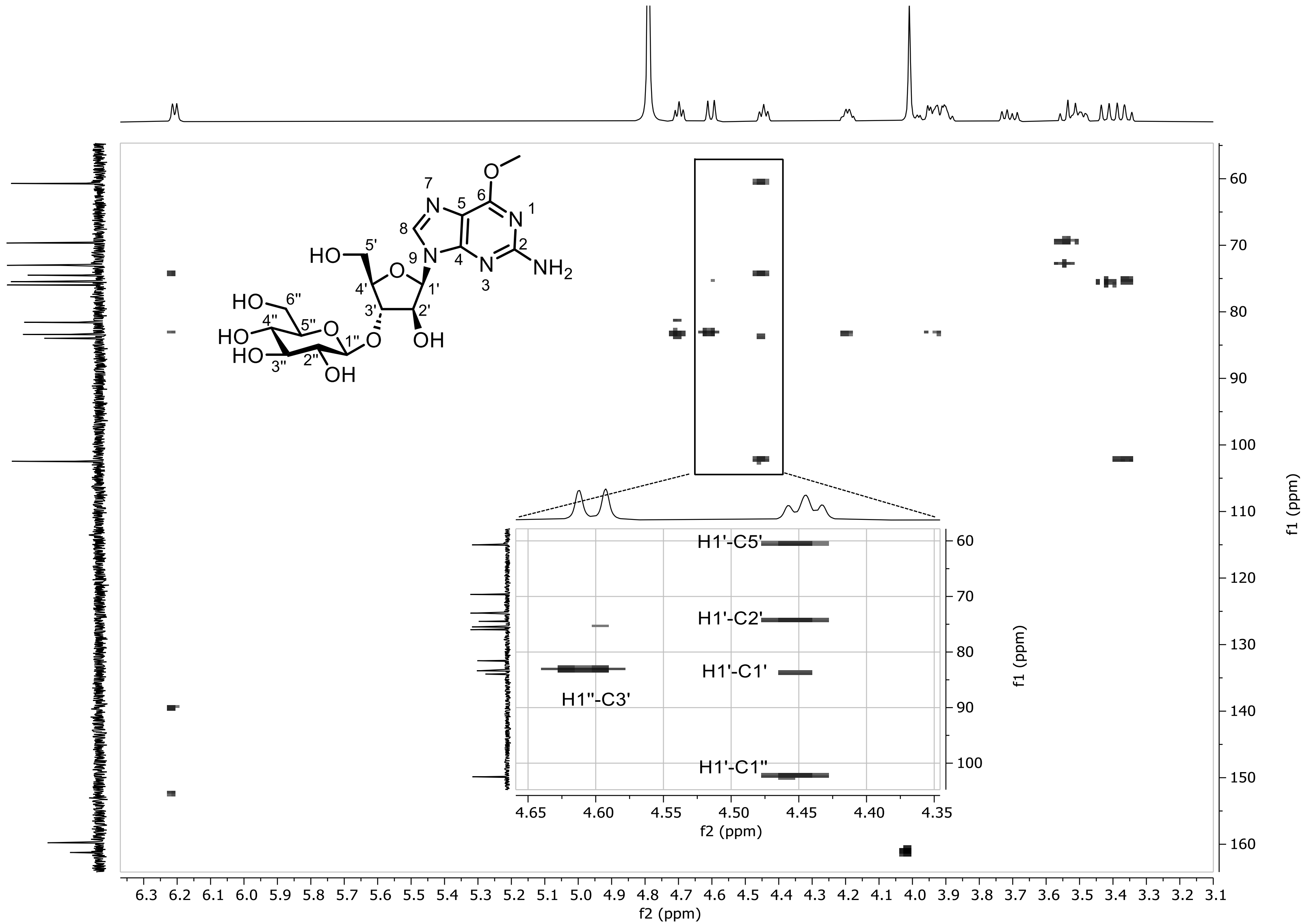

7
8
5
6
1
2
9
4
3
N
N
N
N
O
NH2
HO
5'
4'
1'
3'
2'
OH
HO
6"
4"
5"
1"
HO
HO
3"
2"
OH
H1'-C5'
H1'-C2'
H1'-C1'
H1"-C3'
H1'-C1"
f1 (ppm)
f2 (ppm)

^1H-^{13}C HMBC

3’-O-β-Glucosylfludarabine (18a)

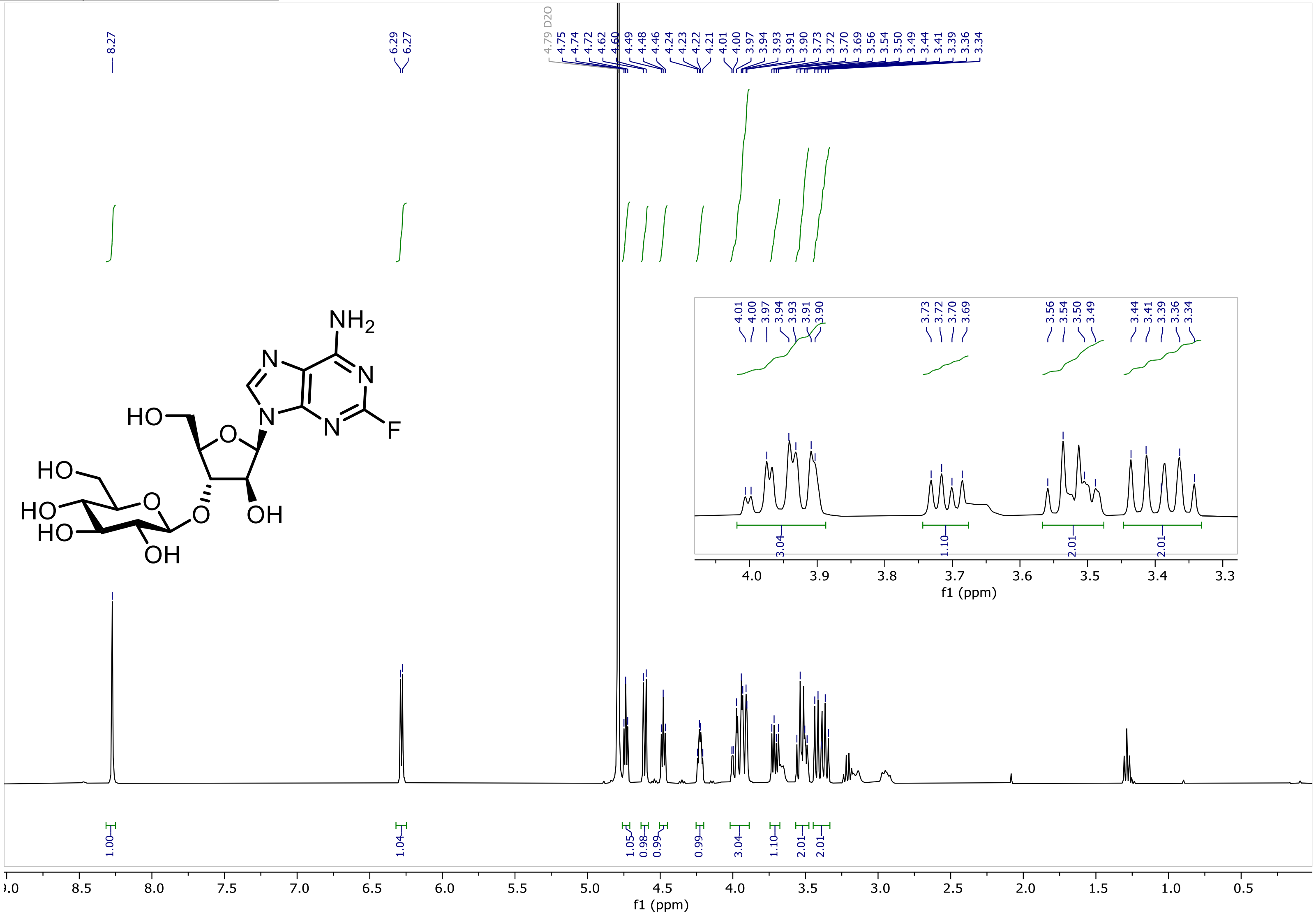

¹H NMR

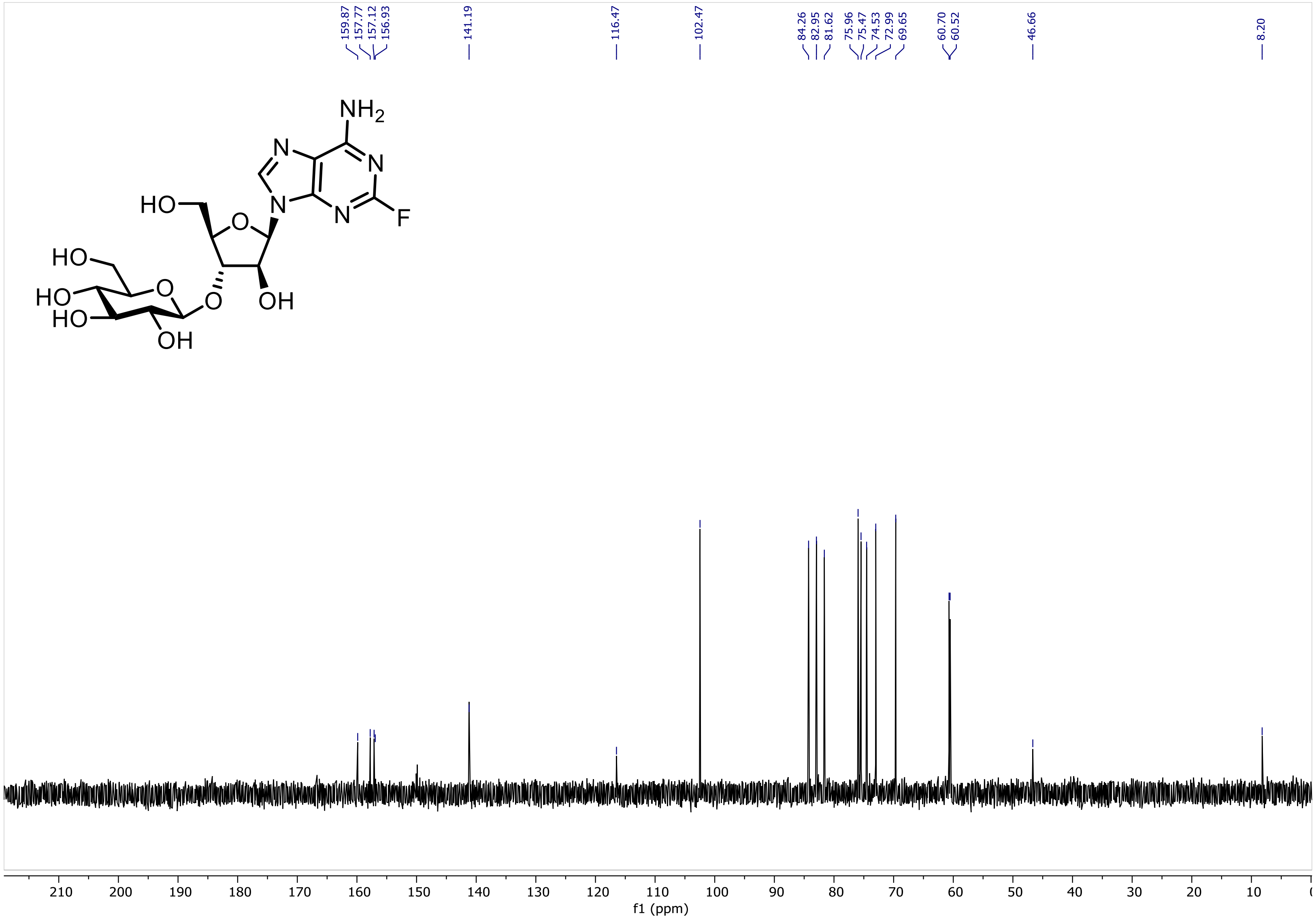
159.87
157.77
157.12
156.93
141.19
116.47
102.47
84.26
82.95
81.62
75.96
75.47
74.53
72.99
69.65
60.70
60.52
46.66
8.20
NH_2
N
N
N
N
F
HO
O
HO
HO
O
HO
O
OH
OH
210 200 190 180 170 160 150 140 130 120 110 100 90 80 70 60 50 40 30 20 10
f1 (ppm)

^{13}C NMR

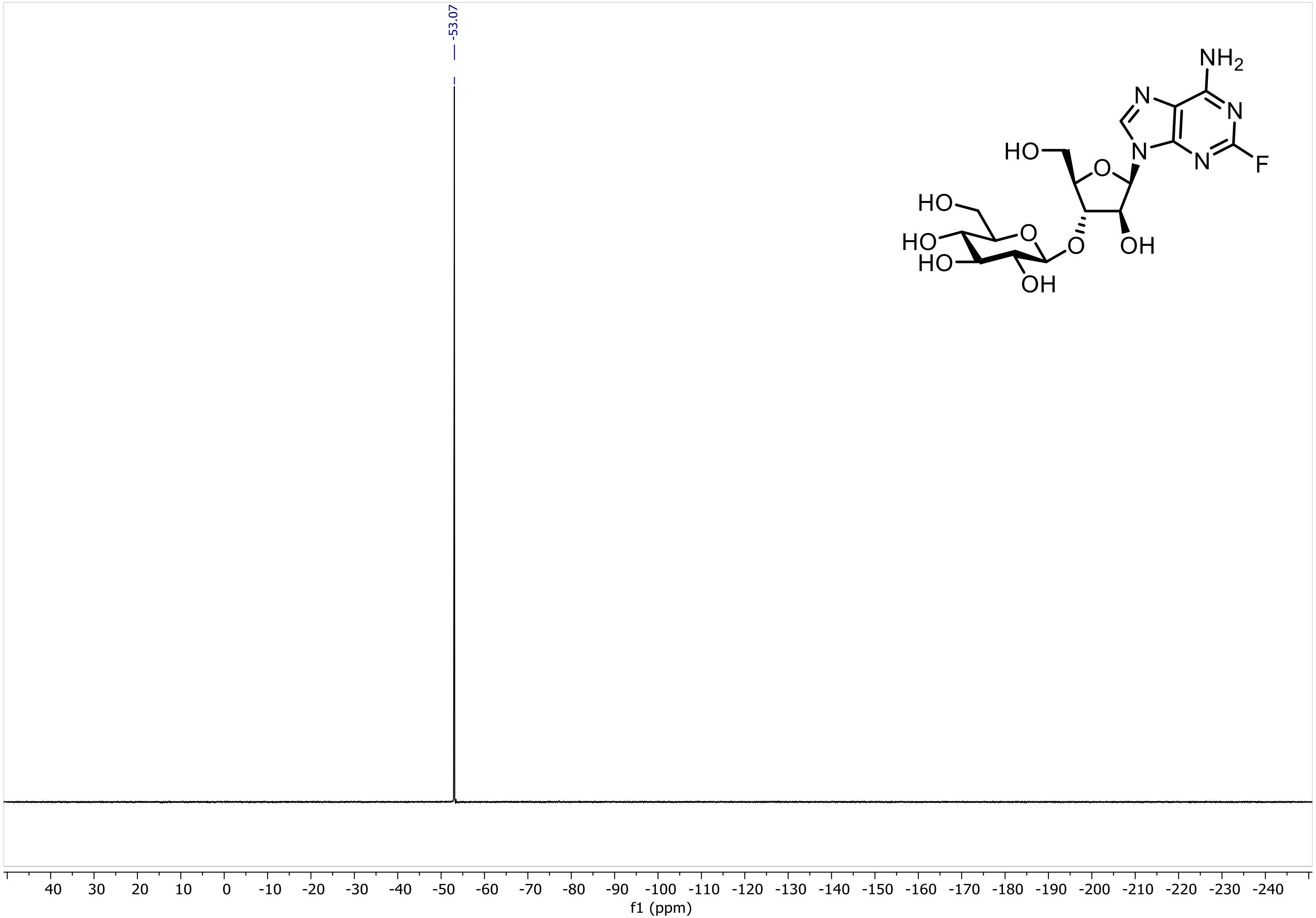
-53.07
NH2
N
N
N
N
F
HO
O
HO
HO
HO
O
O
OH
OH
40 30 20 10 0 -10 -20 -30 -40 -50 -60 -70 -80 -90 -100 -110 -120 -130 -140 -150 -160 -170 -180 -190 -200 -210 -220 -230 -240
f1 (ppm)

^{19}F NMR

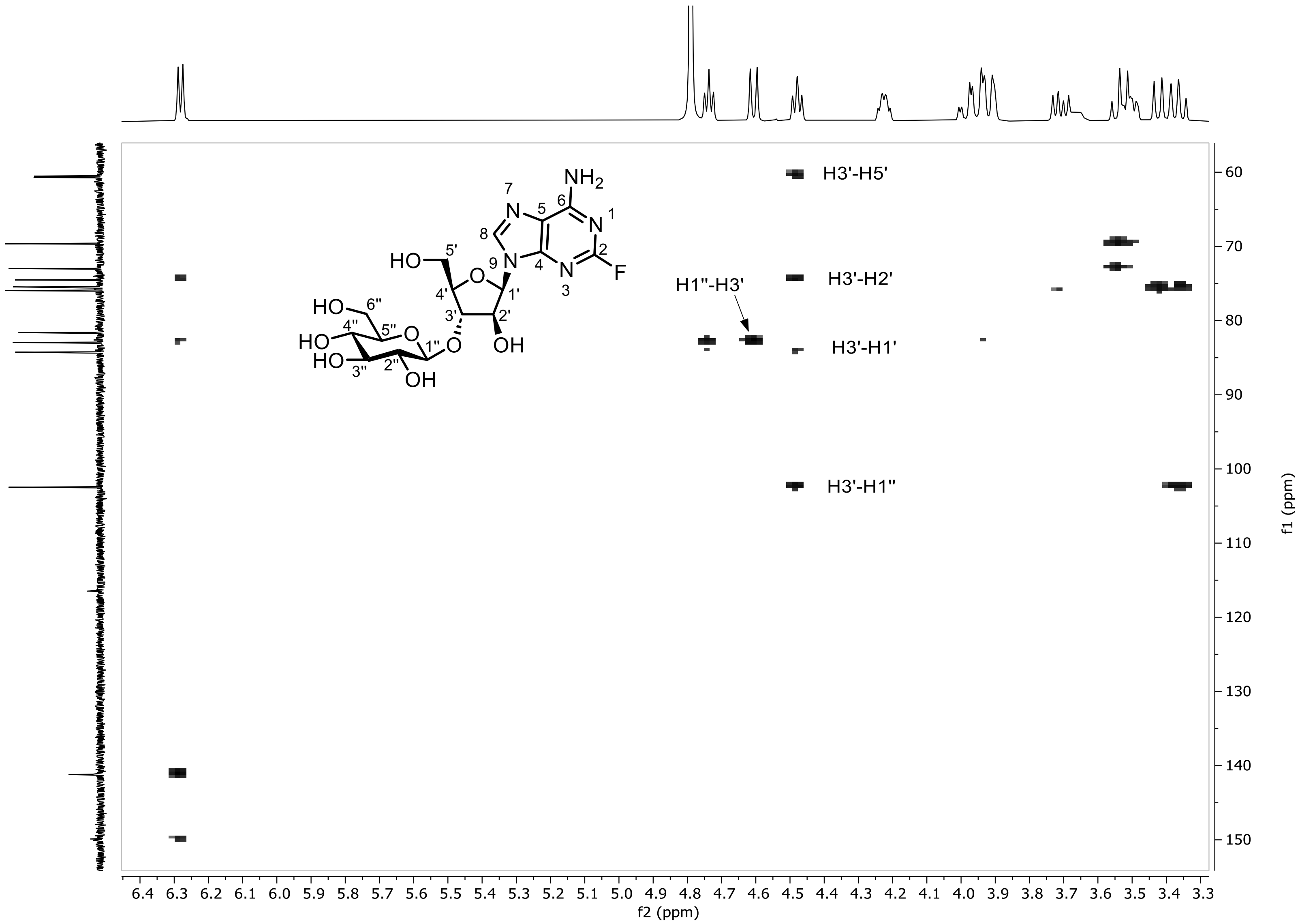

^{1}H-^{13}C HMBC

3’-O-β-Glucosylclofarabine (19a)

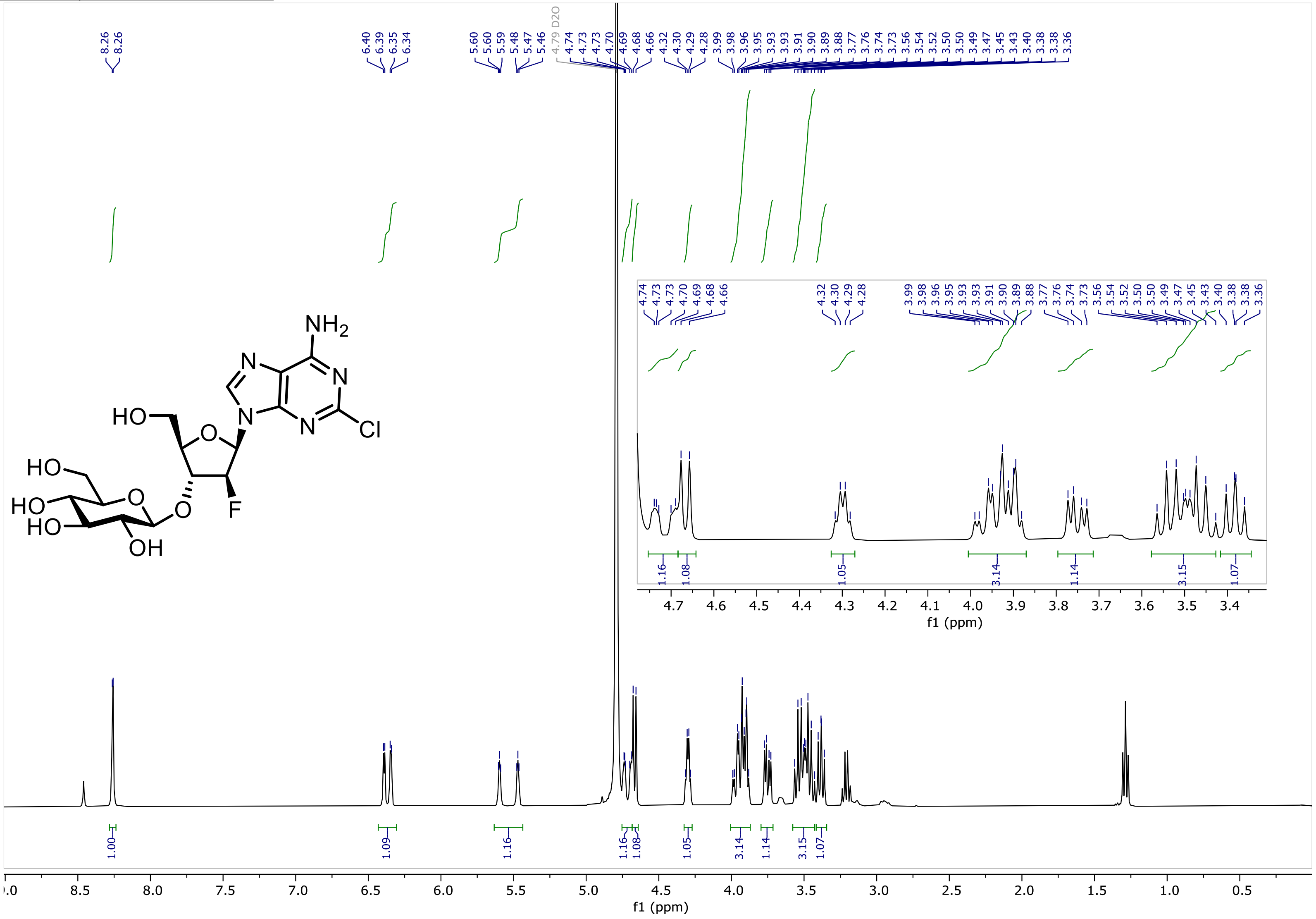

^{1}H NMR

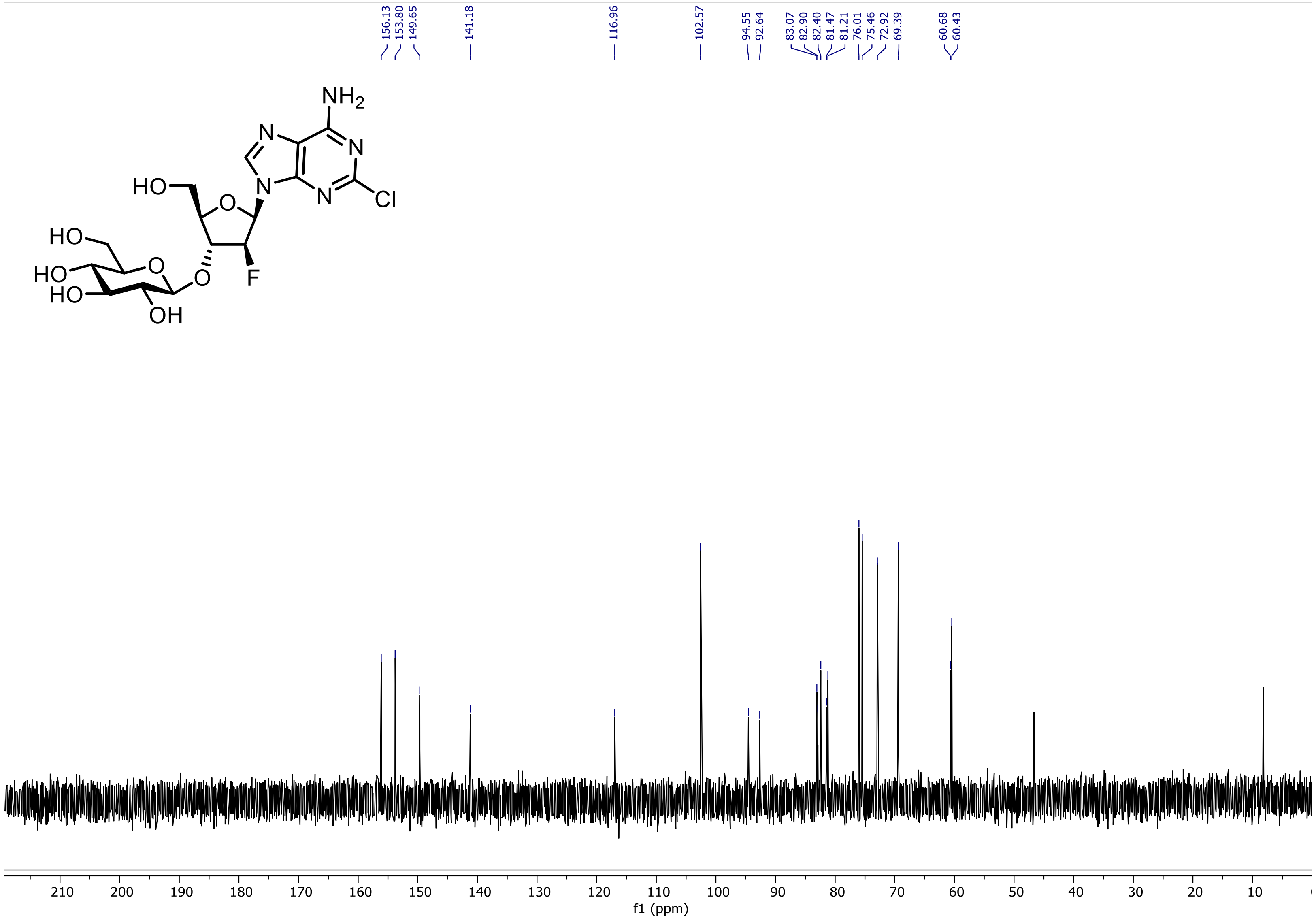
156.13
153.80
149.65
141.18
116.96
102.57
94.55
92.64
83.07
82.90
82.40
81.47
81.21
76.01
75.46
72.92
69.39
60.68
60.43
NH2
N
N
N
N
Cl
HO
O
HO
O
HO
HO
OH
O
F
210 200 190 180 170 160 150 140 130 120 110 100 90 80 70 60 50 40 30 20 10
f1 (ppm)

^{13}C NMR

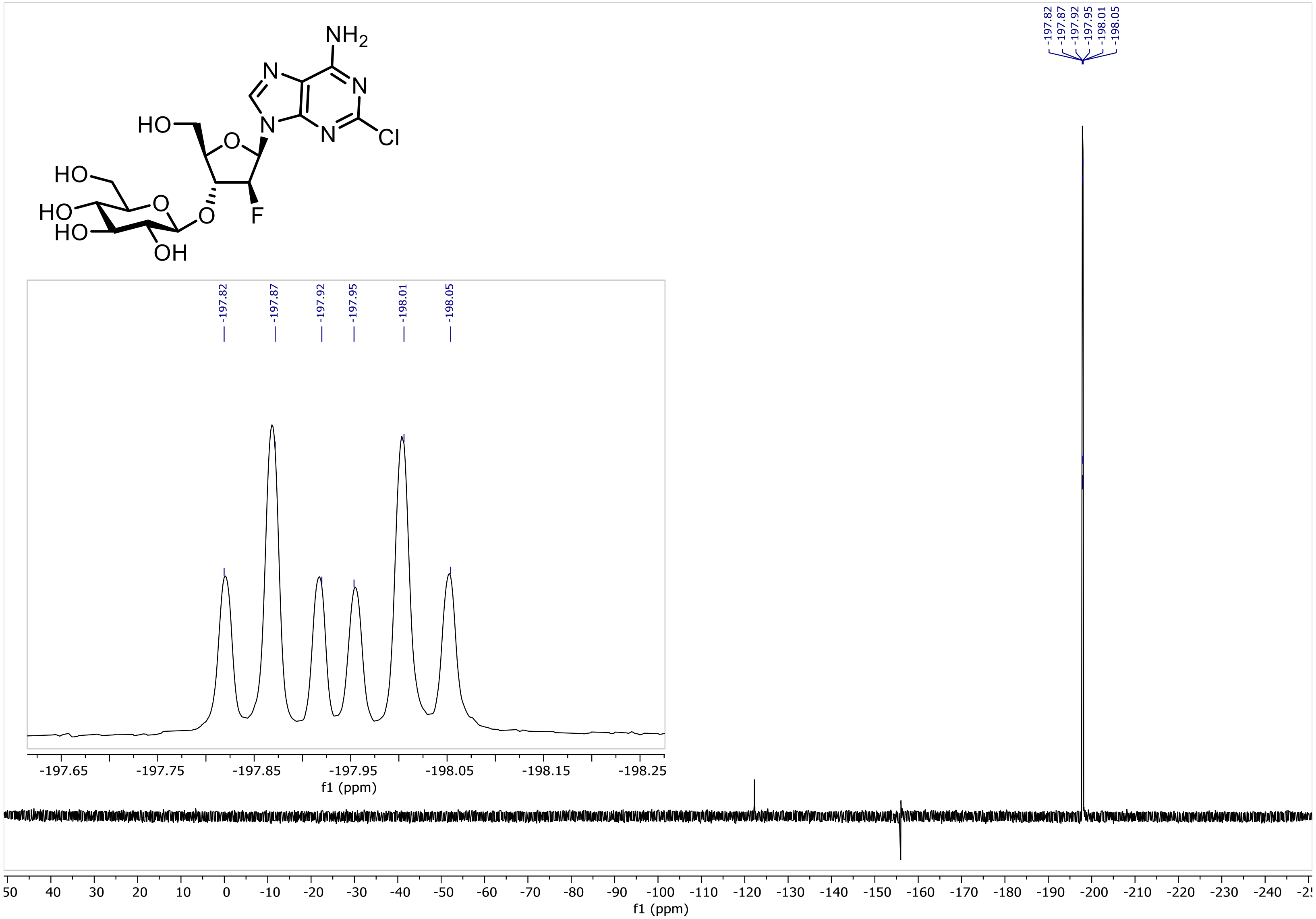

NH2
N
N
N
N
Cl
HO
O
HO
O
HO
HO
OH
O
F
-197.82
-197.87
-197.92
-197.95
-198.01
-198.05
-197.65 -197.75 -197.85 -197.95 -198.05 -198.15 -198.25
f1 (ppm)
50 40 30 20 10 0 -10 -20 -30 -40 -50 -60 -70 -80 -90 -100 -110 -120 -130 -140 -150 -160 -170 -180 -190 -200 -210 -220 -230 -240
f1 (ppm)

^{19}F NMR

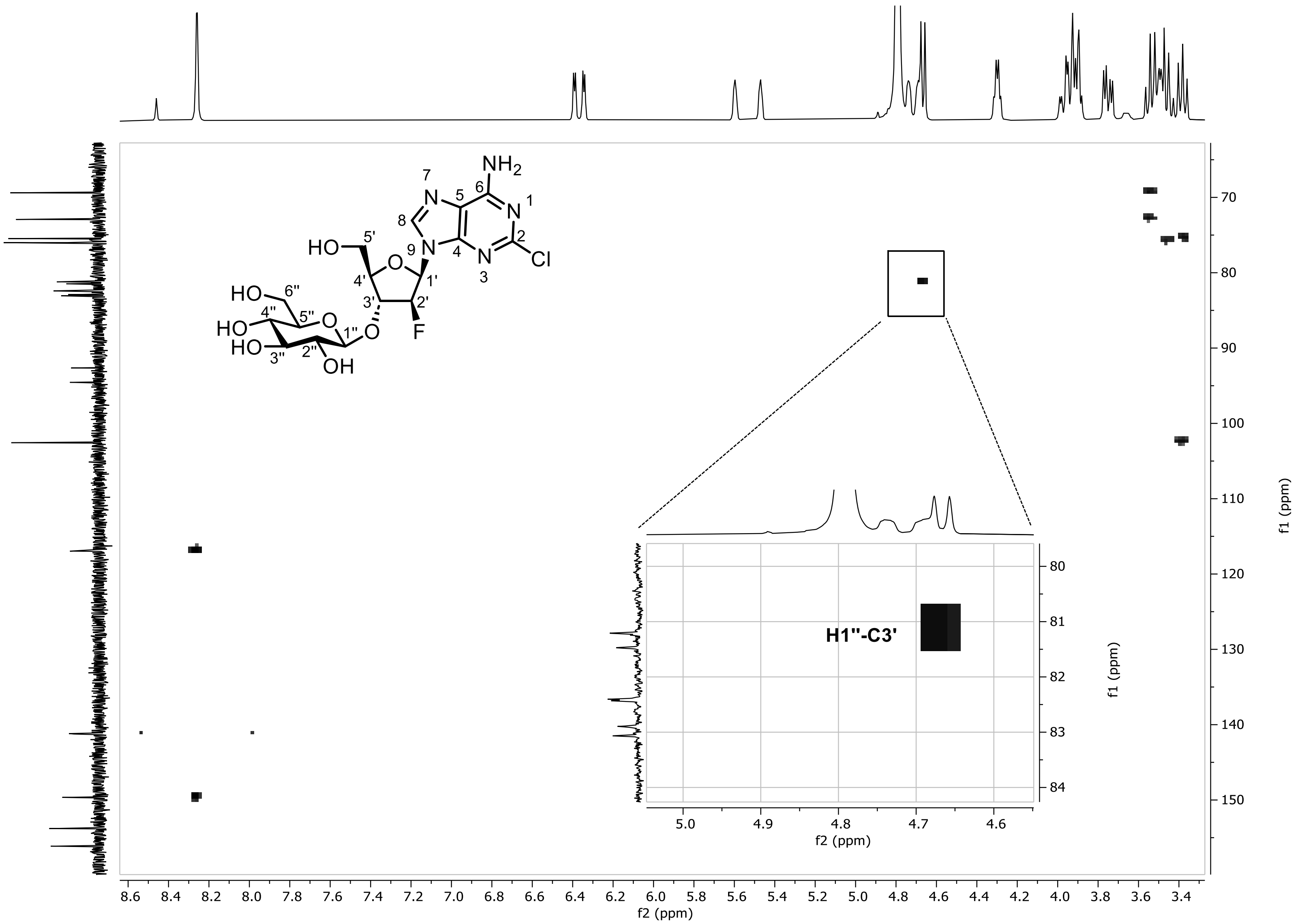
NH2
7
N
5
6
N
1
8
2
HO
5'
9
O
N
4
N
Cl
4'
1'
3
HO
6"
3'
2'
4"
5"
O
HO
1"
O
F
HO
3"
2"
OH
H1"-C3'
f1 (ppm)
f2 (ppm)

^1H-^{13}C HMBC
